# Tetravalent Cerium Alkyl and Benzyl Complexes

**DOI:** 10.1021/jacs.4c01964

**Published:** 2024-04-02

**Authors:** Haruko Tateyama, Andrew C. Boggiano, Can Liao, Kaitlyn S. Otte, Xiaosong Li, Henry S. La Pierre

**Affiliations:** † School of Chemistry and Biochemistry, Georgia Institute of Technology, Atlanta, Georgia 30332-0400, United States; ‡ Nuclear and Radiological Engineering and Medical Physics Program, School of Mechanical Engineering, Georgia Institute of Technology, Atlanta, Georgia 30332-0400, United States; § Department of Chemistry, University of Washington, Seattle, Washington 98195, United States; ∥ Physical Sciences Division, Pacific Northwest National Laboratory, Richland, Washington 99352, United States

## Abstract

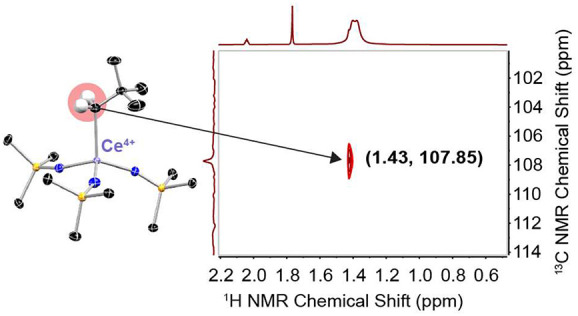

High-valent cerium complexes of alkyl and benzyl ligands are unprecedented due to the incompatibility of the typically highly oxidizing Ce^4+^ ion and the reducing alkyl or benzyl ligand. Herein we report the synthesis and isolation of the first tetravalent cerium alkyl and benzyl complexes supported by the tri-*tert*-butyl imidophosphorane ligand, [NP(^t^Bu)_3_]^1–^. The Ce^4+^ monoiodide complex, [Ce^4+^I(NP(*tert*-butyl)_3_)_3_] (**1-CeI**), serves as a precursor to the alkyl and benzyl complexes, [Ce^4+^(Npt)(NP(*tert*-butyl)_3_)_3_] (**2-CeNpt**) (Npt = neopentyl, CH_2_C(CH_3_)_3_) and [Ce^4+^(Bn)(NP(*tert*-butyl)_3_)_3_] (**2-CeBn**) (Bn = benzyl, CH_2_Ph). The bonding and structure of these complexes are characterized by single-crystal XRD, NMR and UV–vis–NIR spectroscopy, cyclic voltammetry, and DFT studies.

Organometallic chemistry of tetravalent cerium is notably divergent from that of its heavy Group 4 and actinide congeners (Zr, Hf, and Th).^1^ In contrast to organometallic complexes of these tetravalent ions, Ce^4+^ is generally unstable to reduction and the formation of trivalent complexes.^1−3^ As a result, the synthesis and characterization of unsupported alkyls, aryls, and alkylidenes at Ce^4+^ in the condensed phase have not been possible,^4^ despite efforts from several groups since the 1970s.^1^ Given the importance of cerium in catalysis^5−11^ and materials science,^12−14^ a number of inventive coordination chemistry solutions have been explored to stabilize a few representative examples through chelation and/or supporting heteroatoms in the reactive organometallic ligand.^15−20^

Ligand design has recently enabled the stabilization of unusual high-valent lanthanide ions in molecular complexes^21−24^ and facilitated the study of tetravalent terbium^25−30^ and praseodymium.^28,31−33^ We have developed a class of imidophosphorane ligands that stabilize high-valent f-element ions and shift redox couples significantly cathodically.^25,32,34−39^ Rather than modifying the organometallic fragment to enhance its oxidative stability, this approach takes advantage of the strong stabilization of Ce^4+^ by imidophosphorane ligands to afford hitherto unrealized organometallic coordination chemistry. All tetravalent imidophosphorane f-element complexes reported to date are homoleptic complexes,^25,26,34−36,39,40^ and a key innovation in this study is the preparation of a heteroleptic, monohalide complex, [Ce^4+^I(NP((*tert*-butyl)_3_)_3_] (**1-CeI**). This complex facilitates the isolation and characterization of a Ce^4+^-alkyl complex, [Ce^4+^(CH_2_C(CH_3_)_3_)(NP(*tert*-butyl)_3_)_3_], (**2-CeNpt**) (Npt = neopentyl, CH_2_C(CH_3_)_3_), with an unsupported Ce–C σ-bond and a Ce^4+^-η^2^-benzyl complex, [Ce^4+^(η^2^-CH_2_Ph)(NP(*tert*-butyl)_3_)_3_], (**2-CeBn**) (Bn = benzyl, CH_2_Ph). Notably, **2-CeNpt** is the first example of a Ce^4+^ alkyl complex with an unsupported, sp^3^-hybridized C in a C–Ce^4+^ σ-bond.

The critical precursor, **1-CeI**, is prepared by the addition of a diethyl ether (Et_2_O) solution of 2.5 equiv of HNP(^t^Bu)_3_ and 2.5 equiv of potassium benzyl (KBn) to a slurry of CeI_3_(THF)_4_ (THF = tetrahydrofuran) in Et_2_O. After 48 h, the reaction mixture is filtered and 1.05 equiv of AgI is added, producing **1-CeI** in 44–58% yield (based on HNP(^t^Bu)_3_). Ce^4+^ iodides are relatively uncommon due to the instability of the Ce^4+^ ion to reduction by iodide.^41−47^

Subjecting a Et_2_O solution of **1-CeI** to a Et_2_O solution of 1.05 equiv of KBn at −35 °C leads to the isolation of **2-CeBn** as deep green crystals (63% yield). The choice of solvent for the reaction and crystallization is critical to avoid reduction. Exposure to THF leads to decomposition as evidenced by bleaching of the solution and ^31^P{^1^H} NMR spectroscopy (Figure S12).^2^ The reaction and crystallization conditions for **2-CeBn** served as a methodological roadmap for the isolation of **2-CeNpt**. The reaction of **1-CeI** with 1.05 equiv of LiCH_2_C(CH_3_)_3_, in Et_2_O at −35 °C affords **2-CeNpt** as black crystals (25% yield).

Complexes **1-CeI** and **2-CeNpt** are four-coordinate (Figure 1B, C), and **2-CeBn** is five-coordinate with an η^2^-benzyl ligand (Figure 1D) as determined by single crystal X-ray diffraction (SC-XRD). Average Ce–N and N–P bond distances and Ce–N–P bond angles across the three complexes are consistent with those of previous Ce^4+^ tetrahomoleptic imidophosphorane complexes (Table S2). The Ce–I bond distance is 3.091(1) Å (Figure 1B), which is within the range of known Ce^4+^ iodide complexes (2.851–3.181 Å).^41−47^

**Figure 1:**
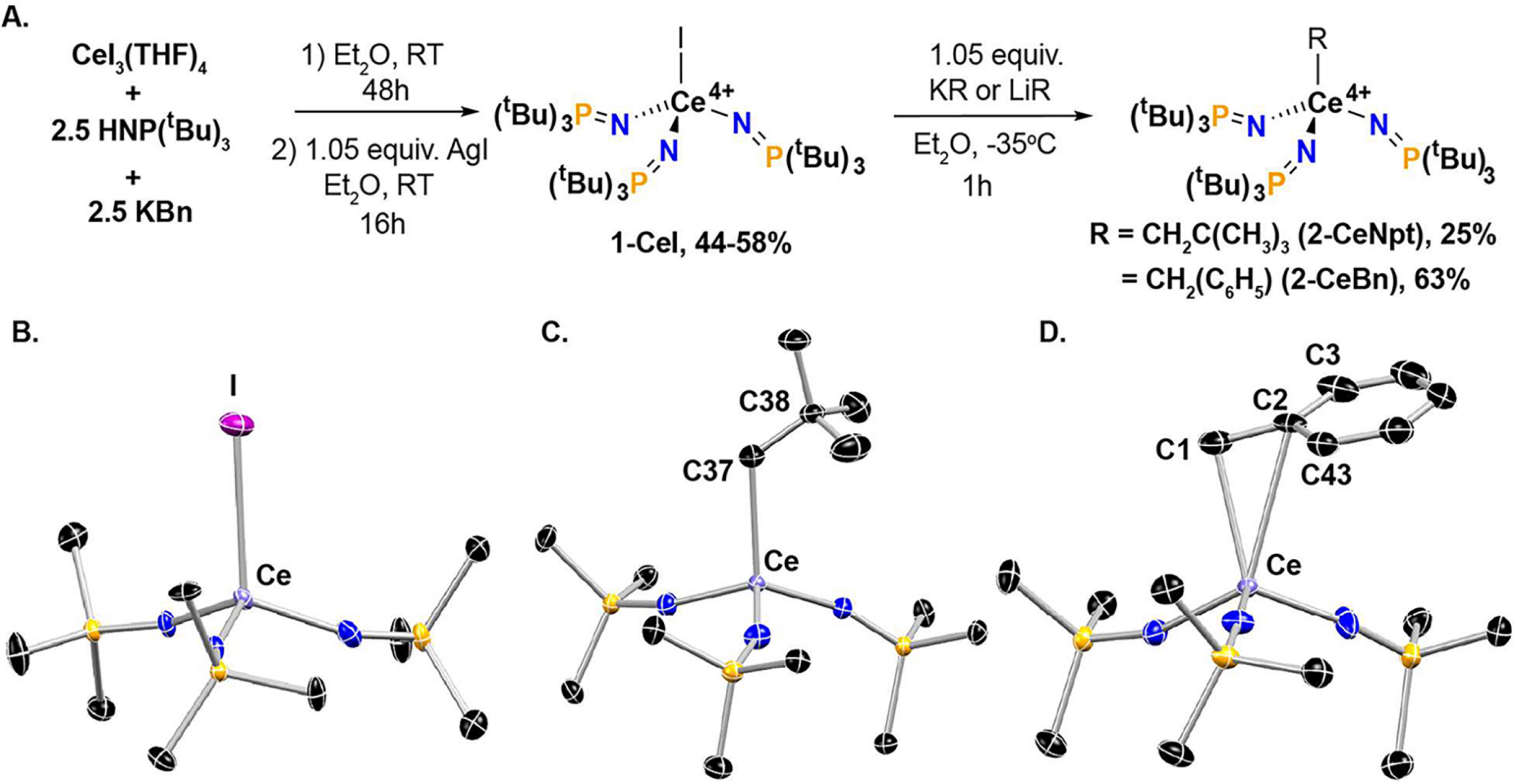
(A) Synthetic route to **1-CeI**, **2-CeNpt**, and **2-CeBn.** Truncated molecular structures determined by SC-XRD of (B) **1-CeI**, (C) **2-CeNpt**, and (D) **2-CeBn** with thermal ellipsoids (Ce: purple, C: black, P: orange, N: blue, I: magenta) shown at 50% probability. All hydrogens and imidophosphorane CH_3_ have been omitted for clarity.

The Ce–C distances in **2-CeBn** indicate η^2^-coordination of the benzyl ligand (Table S17 and Figure S37)^48^ with Ce^4+^–C bonds distances to the methylene carbon, C1, and the *ipso*-carbon, C2, at 2.562(2) and 2.948(2) Å, respectively. The Ce–C1–C2 angle (90.4°) is consistent with the η^2^ assignment. The Ce–C distance in **2-CeNpt** is 2.508(2) Å, and the Ce–C–C angle is 122.7(2)°. This Ce–C bond length falls within the range expected for Ce^4+^ (Figure S38).

Solution NMR spectra of **1-CeI**, **2-CeBn**, and **2-CeNpt** are consistent with the SC-XRD determined structures. The complexes **1-CeI** and **2-CeBn** exhibit remarkable thermal stability, showing no thermal decomposition up to 60 and 80 °C, respectively, as demonstrated by ^31^P{^1^H} NMR (Figures S19–S22). Crucially, the resonances of the benzyl and neopentyl methylene carbons can be assigned via ^1^H–^13^C HSQC and ^13^C DEPT-135 NMR spectroscopies (Figures 2, S11, and S16–18). The downfield shifts of these methylene resonances at 90.12 (**2-CeBn**) and 107.85 ppm (**2-CeNpt**), are dispositive for the direct bonding of the sp^2^- and sp^3^-hybridized carbon atoms to cerium. This shift is clear in light of the observed shifts for the methylene carbons in KBn (52.6 ppm)^49^ and LiNpt (35.8 ppm).^50^ Coordination to tetravalent 4d, 5d, or actinide metals (Zr, Hf, or Th) also leads to a downfield shift of this resonance (in ppm, Bn: Zr, 72.5; Hf, 82.3; Th, 86.85; Npt: Zr, 97.45; Hf, 104.97; Th, 117.96). The Bn series are [M(Bn)_4_].^48,51,52^ For Npt, no complete isostructural series is known, and the most downfield resonances are selected for comparison.^53−55^

**Figure 2:**
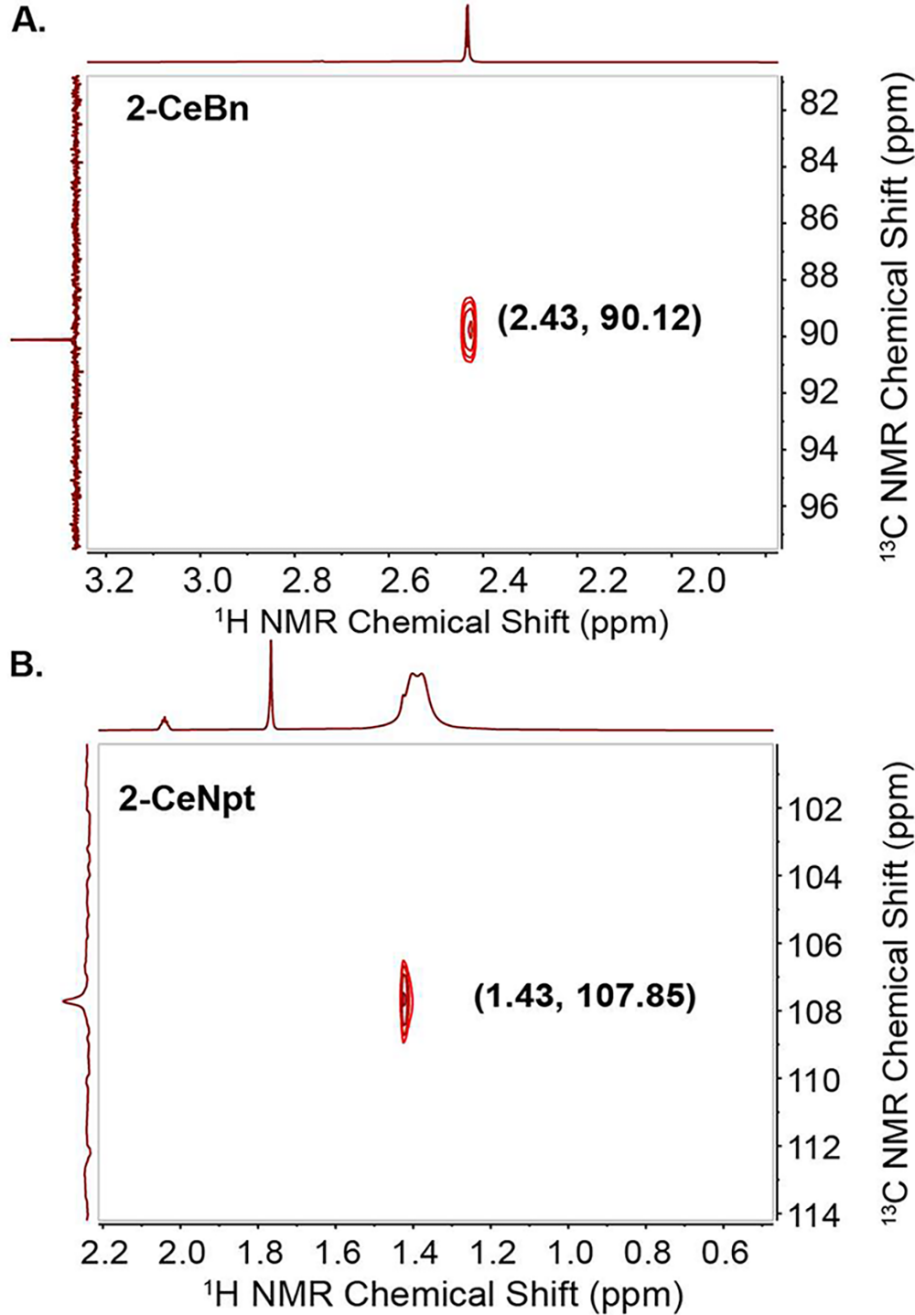
Partial ^1^H–^13^C HSQC spectra showing the C–H coupling of the Ce^4+^–CH_2_ proton to the metal-bonded ^13^C: (A) **2-CeBn**, where the methylene proton at 2.43 ppm correlates with the ^13^C resonance at 90.12 ppm (298 K), and (B) **2-CeNpt**, where the methylene proton at 1.43 ppm correlates with the ^13^C resonance at 107.85 ppm (238 K).

The downfield shift of the resonance of carbon atoms bound to heavy atoms is due to the SO-HALA (spin–orbit heavy-atom on the light atom) effect.^56^ The magnitude of the spin–orbit induced shift of the resonance depends on the hybridization of the bound carbon atom (degree of carbon s character in the bond), the percentage of the principal metal valence orbitals in the bond, and total percentage of metal contribution to the bond.^57−59^ Importantly, f-orbital contribution to the bond leads to a stronger SO-HALA effect than d-orbital contribution.^57,60,61^ Therefore, the relative downfield shift of the ^13^C NMR resonance of carbons directly bonded to heavy d- and f-block metals has been developed as a reporter of metal–ligand bond covalency in f-element organometallic complexes.^17,18,62,63^

The bonding in **2-CeBn** and **2-CeNpt** was examined by density functional theory (DFT), using the SC-XRD structure geometries with the B3LYP functional (see ESI for computational details and orbital figures).^64^ For both molecules, the 4f-dominant MOs (LUMO – LUMO+6) are largely metal-centered, typical of Ce^4+^ compounds.^65,66^ The HOMO for both molecules are Ce–C σ-bonding orbitals (Figure 3). Both the benzyl and neopentyl ligands are weakly bonded to Ce with bond orders much less than 1 (Table S9).^67^ Though the majority of the bonding between Ce and the benzyl ligand occurs at the benzylic carbon, some interaction can be seen between Ce and both C2 and C43 (Figure 3A). This interaction is demonstrated by the small but significant bond order and delocalization index between Ce and C43 as well as the slight tilt of the benzyl group toward Ce observed via SC-XRD.

**Figure 3:**
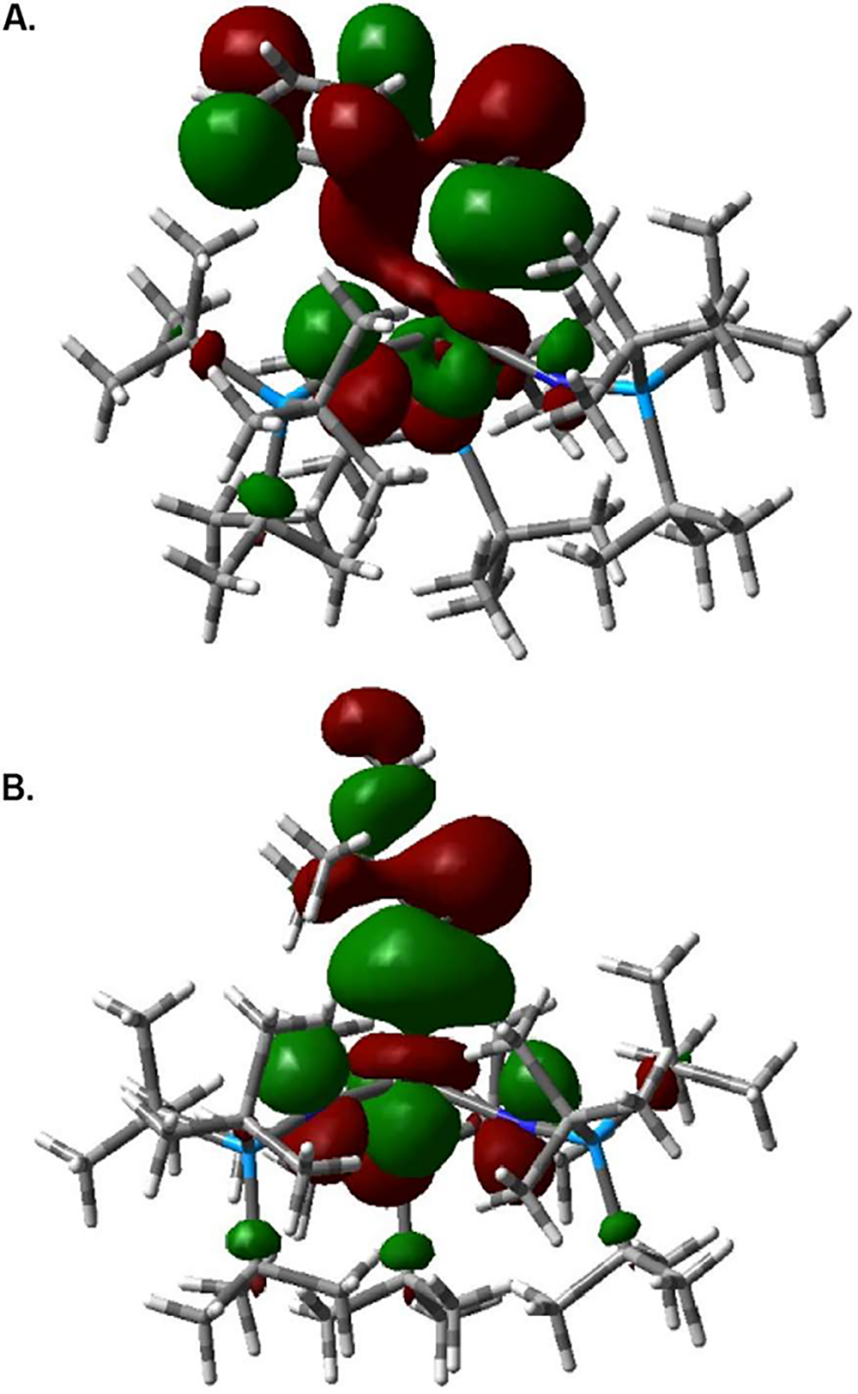
DFT/B3LYP HOMO of (A) **2-CeBn** and (B) **2-CeNpt** showing the interaction between Ce and the ligand plotted at an isovalue of 0.02.

The Ce contribution to the Ce–Npt bond is 9.3% Ce, of which 48.6% is 5d and 37.2% is 4f character (Table S9). This larger contribution of the 5d character to the bonding (relative to **2-CeBn**) leads to a less dramatic SO-HALA effect. Consequently, the resonance of the methylene carbon of **2-CeNpt** is intermediate between the resonances of the reference Hf and Th complexes. In contrast, in **2-CeBn**, the methylene carbon experiences a greater SO-HALA effect, and the resonance is further downfield than the reference Zr, Hf, and Th complexes. The Ce contribution to the Ce–Bn bond has a similar total metal character, 9.6%, but differs in its metal valence character (35.9% d and 55.9% f character, Table S9). The increased f-orbital participation in the bonding of **2-CeBn** rationalizes the comparatively larger SO-HALA effect. Chemical shifts computed for **2-CeBn** and **2-CeNpt** support this bonding model, and find a chemical shift for the methylene carbon of **2-CeBn** at 79 ppm and for **2-CeNpt** at 101 ppm. These results are qualitatively in-line with the relative accuracy of NMR chemical shift calculations of f-element complexes.^17,18,62,63^

The tetravalent oxidation state of cerium in **1-CeI**, **2-CeBn**, and **2-CeNpt** was further confirmed by electronic absorption spectroscopy. The UV–vis–NIR spectra of **1-CeI**, **2-CeBn**, and **2-CeNpt** all show high-energy features (λ_max_ = 395 nm, ε = 5300 M^–1^cm^–1^; λ_max_ = 326 nm, ε = 6000 M^–1^cm^–1^ and λ_max_ = 357 nm, ε = 5500 M^–1^cm^–1^; λ_max_ = 365 nm, ε = 8700 M^–1^cm^–1^, respectively; Figure 4A), which are ligand to metal charge transfer (LMCT) features consistent with other Ce^4+^ imidophosphorane complexes.^34,35^ Spectra of both **2-CeBn** and **2-CeNpt** also present a lower-energy feature (602 nm, ε = 600 M^–1^cm^–1^; 545 nm, 1500 M^–1^cm^–1^).

**Figure 4:**
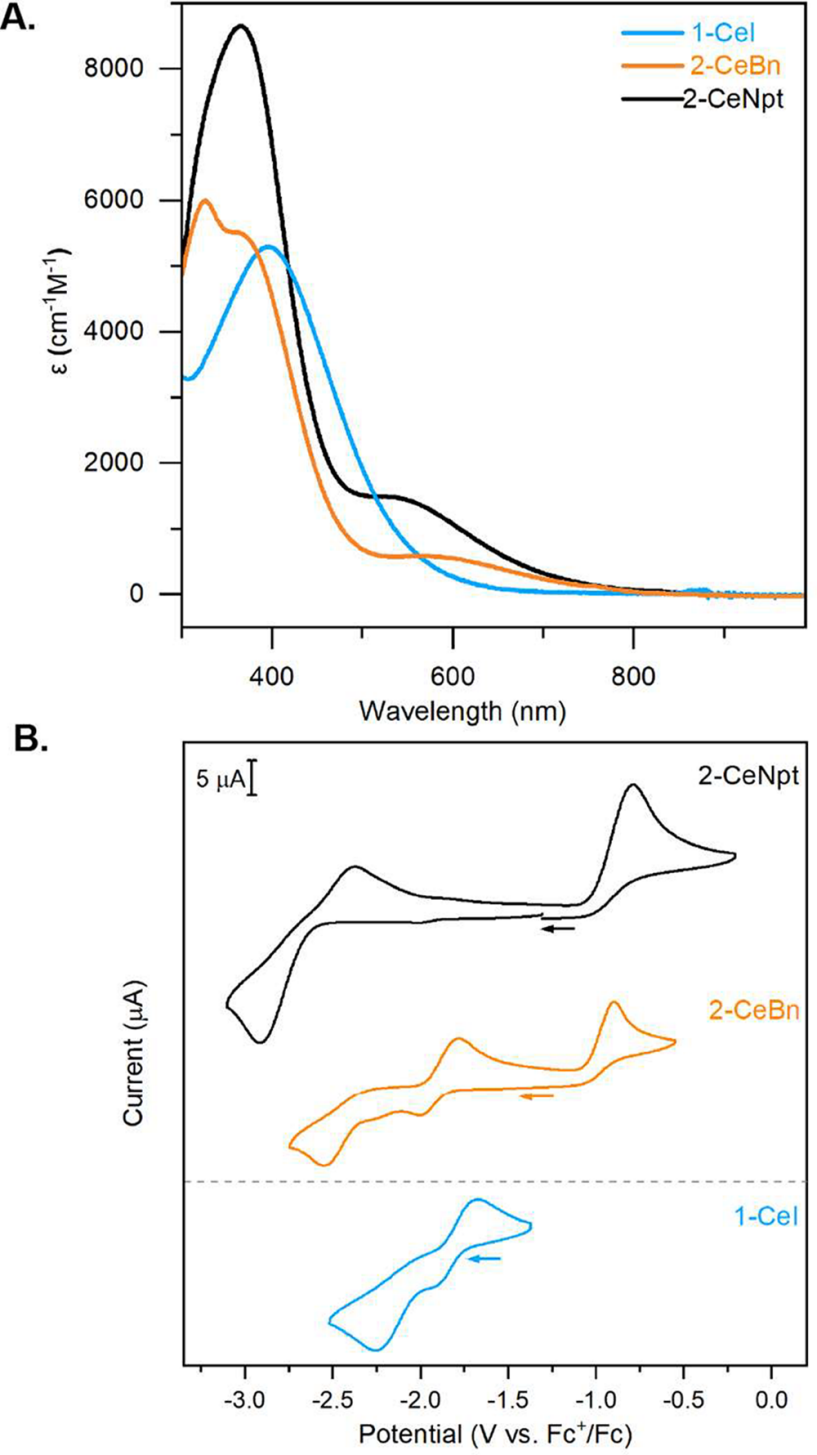
(A) Co-plotted molar absorptivity of **1-CeI** (140 μM in toluene), **2-CeBn** (140 μM in benzene), and **2-CeNpt** (140 μM in Et_2_O) at 298 K. (B) Cyclic voltammograms of **1-CeI** (3 mM in 0.1 M [^n^Bu_4_N][BPh_4_] in THF), **2-CeBn**, and **2-CeNpt** (3 mM in 0.2 M [^n^Bu_4_N][PF_6_] in PhF) at 298 K. All potentials are referenced against Fc^+^/Fc.

The UV–vis–NIR spectra of **2-CeNpt** and **2-CeBn** were simulated using linear response time-dependent DFT with perturbative SOC,^68^ though incorporating SOC did not significantly alter the spectra. The **2-CeBn** theoretical spectrum (Figure S36) exhibits a low-energy peak around 603 nm which originates from a linear combination of Ce–Bn σ (HOMO) → 4f (LUMO – LUMO+6) orbital excitations defining it as LMCT in character. The higher-energy peak arises from a linear combination of Ce–N π (HOMO–6 – HOMO–5) → 4f orbital excitations and is therefore an LMCT. The **2-CeNpt** theoretical spectrum (Figure S36) also agrees well with experiment. The two peaks in the **2-CeNpt** spectrum both originate from LMCT excited states. The excited state responsible for the low-energy peak arises from Ce–Npt σ (HOMO) → 4f_z^3^_ (LUMO) orbital excitation. The main LMCT excited states responsible for the high-energy peak arise from a linear combination of Ce–N π (HOMO–6 – HOMO–5) → 4f (LUMO – LUMO+6) orbital excitations.

The redox properties of these complexes were investigated through cyclic voltammetry (CV); all display significantly negative (<−2.0 V vs Fc^+^/Fc) reduction potentials (Figure 4B). Electrochemical reductions of **2-CeNpt** and **2-CeBn** were measured at E_pc_ = −2.92 V and E_pc_ = −2.55 V vs Fc^+^/Fc, respectively. The CVs of **2-CeNpt** and **2-CeBn** are similar to those of σ-aryl complexes reported by Schelter and co-workers,^14^ with two oxidative waves observed, assigned as a metal-centered and an alkyl/aryl-centered oxidation. The metal-centered oxidations (E_pa1_) of **2-CeNpt** and **2-CeBn** are observed at potentials of −2.38 V and −1.78 V vs Fc^+^/Fc, respectively. The more positive E_pa1_ value of **2-CeNpt** vs **2-CeBn** mirrors the trend observed for E_pc_ and further supports the increased stabilization of the tetravalent oxidation state in **2-CeNpt**. A second oxidative wave (E_pa2_) at more positive potentials is observed for **2-CeNpt** (E_pa2_ = −0.79 V vs Fc^+^/Fc) and **2-CeBn** (E_pa2_ = −0.90 V vs Fc^+^/Fc), consistent with irreversible oxidation of the anionic neopentyl or benzyl substituents, respectively.

In the CV of **1-CeI** (Figure 4B) in THF, no second oxidation is observed within the electrochemical window, which agrees with the higher oxidative stability of I^–^ vs Bn^–^ and Npt^–^. The lack of a second oxidative feature in the CV of **1-CeI** supports the assignment of E_pa2_ as the oxidation of the organometallic moieties in **2-CeNpt** and **2-CeBn**. **1-CeI** displays the most positive E_pc_ value at −2.25 V vs Fc^+^/Fc, suggesting the tetravalent oxidation state is least-stabilized by I^–^ compared to the organometallic ligands in **2-CeNpt** and **2-CeBn**. See ESI for further analysis of the CVs.

In summary, we report the isolation and characterization of Ce^4+^ neopentyl and benzyl complexes. The typically oxidizing Ce^4+^ ion is stabilized by strongly donating imidophosphorane ligands. This methodology presents an opportunity to greatly expand the organometallic chemistry of high-valent lanthanides.

## Supporting Information Available

Experimental details, NMR and UV–vis–NIR spectra, electrochemical analysis, computational details, and crystallographic data (PDF)
