# Supplementary material for: Tetravalent Cerium Alkyl and Benzyl Complexes
Source: J Am Chem Soc. 2024 Apr 2;146(15):10268–73. doi: 10.1021/jacs.4c01964 (PMC11027143; doi:10.1021/jacs.4c01964)
Supplement: Supplementary file 1 — ja4c01964_si_001.pdf [file ja4c01964_si_001.pdf]

Supporting Information for:

Tetravalent Cerium Alkyl and Benzyl Complexes

Haruko Tateyama†, Andrew C. Boggiano†, Can Liao§, Kaitlyn S. Otte†, Xiaosong Li§, and Henry S. La Pierre*†,‡,||

†School of Chemistry and Biochemistry, Georgia Institute of Technology, Atlanta, Georgia 30332-0400, United States

‡Nuclear and Radiological Engineering Program, Georgia Institute of Technology, Atlanta, Georgia 30332-0400, United States

§Department of Chemistry, University of Washington, Seattle, Washington 98195, United States

||Physical Sciences Division, Pacific Northwest National Laboratory, Richland, Washington 99352, United States

Table of Contents

Table of Contents 1

General Considerations 2

Synthetic Methods 3

NMR Spectroscopy 5

Thermal Stability Study by NMR 14

Electrochemistry 17

UV-vis-NIR Electronic Absorption Spectra 22

Crystallographic Analyses 24

Computational Details 36

Hapticity Assignment of 2-Bn 57

Comparison of Group 4 (Zr, Hf) and Th Metal-Carbon Distances in Neopentyl Complexes 58

References 59

General Considerations

Unless otherwise noted, all reagents were obtained from commercial suppliers. Manipulations and syntheses were conducted using Schlenk line techniques under Ar or in a N_2 filled glovebox (Vigor, <0.1ppm O_2/H_2O). All glassware were dried at a temperature of ca. 160°C for >8h. Celite and molecular sieves(3Å) were dried under vacuum at >250 °C for more than 24 hours. Pentane, hexanes, diethyl ether, and toluene were purged with UHP Ar gas (Airgas) and dried through the columns in a commercial solvent purification system (JC Meyer Solvent Systems), and stored over 3 Å molecular sieves in the glovebox. C_6D_6 was dried over 3 Å molecular sieves and vacuum transferred prior to use. Hexamethyldisiloxane (HMDSO) was dried over 3 Å molecular sieves, then distilled and stored over 3 Å molecular sieves. $(CH_3)_3CCH_2Br$ was degassed and dried over 3 Å molecular sieves prior to use. Fluorobenzene (PhF) was dried over CaH_2 at reflux under Ar, then distilled and stored over 3 Å molecular sieves. P^tBu_3 (>99%) was purchased from STREM and used as received. $CeI_3(THF)_4$,[1] $HNP(^tBu)_3$,[2,3] and benzyl potassium (KBn)[4] were prepared by previously reported procedures. Neopentyl lithium was prepared as previously reported,[5] using neopentyl bromide in place of neopentyl chloride. Electrochemical analysis: $[^nBu_4N][PF_6]$ (Oakwood) was recrystallized three times from absolute ethanol then dried under vacuum at 100 °C prior to use. $[^nBu_4N][BPh_4]$ was prepared as previously reported[6] and dried under vacuum at 85 °C for 24 h prior to use. Ferrocene and decamethylferrocene were sublimed before use. Electrochemical data were measured using a Pine WaveDriver 20 Bipotentiostat/Galvanostat. Measurements were performed in a glovebox under an atmosphere of N_2 with a glassy carbon working electrode (3 mm diameter), a bare Ag wire reference electrode in a fritted capillary filled with the corresponding electrolyte solution, and a platinum wire counter electrode at ambient temperature (~25 °C, 298 K). The fritted capillary was stored in electrolyte solution when not in use, and the inner solution was replaced with fresh electrolyte solution prior to use. The glassy carbon and Ag wire electrodes were polished before use. Electrolyte solutions were prepared in THF (**1-CeI**), and PhF (**2-CeBn** and **2-CeNpt**) since **2-CeBn** is shown to decompose in THF (Fig. S12). Measurements were made in positive feedback iR compensation mode (~500 Ω for 0.1 M $[^nBu_4N][BPh_4]$ in THF, ~800 Ω for 0.2 M $[^nBu_4N][PF_6]$ in PhF). Voltammograms were referenced by adding a small amount of ferrocene (0.2 M $[^nBu_4N][PF_6]$ in PhF) or decamethylferrocene (0.1 M $[^nBu_4N][BPh_4]$ in THF) at the end of each experiment. All potentials are reported vs. Fc^+/Fc, using the conversion of decamethylferrocene $E^{\circ\prime}$ = -0.5 V vs. Fc/Fc+ for 0.1 M $[^nBu_4N][BPh_4]$ in THF.[7]

NMR spectra were obtained on a Bruker Avance III 400 MHz or 500MHz spectrometer at 298 K, for **1-CeI** and **2-CeBn**. NMR spectra of **2-CeNpt** were obtained at 238 K. ^1H NMR spectra and $^{13}C\{^1H\}$ spectra are referenced to the residual ^1H resonances of the deuterated solvent unless otherwise noted. $^{31}P\{^1H\}$ NMR spectra are referenced using an absolute reference to H_3PO_4. Peak position is reported, followed by peak multiplicity, integration value, and assignment where applicable. Abbreviations for the peak multiplicity are as follows: s (singlet); d (doublet); t (triplet); m (multiplet). Infrared (IR) spectroscopy was conducted on a Bruker ALPHA FTIR Spectrometer from 400 to 4000cm^{-1}, using an ATR attachment inside of a N_2 glovebox. The intensities of the peaks are reported using the following abbreviations: vw (very weak); medium (medium); s (strong); vs (very strong); br (broad). UV-vis NIR spectroscopy was conducted on a Hitachi UH4150 UV-vis-NIR scanning spectrophotometer from 1000 nm to 300 nm. Characterization was performed in small-volume screw cap quartz cuvettes (Starna Scientific) with a 1cm path length. Elemental analyses on C, H, N were performed at the University of Berkeley Microanalytical Facility (Berkeley, CA), where V_2O_5 catalyst was added for the analysis. SC-XRD was performed at Georgia Institute of Technology X-ray Crystallography Facility on a Bruker D8 Venture diffractometer.

Synthetic Methods

HNP^{t}Bu$_3$. This synthesis is a two-step, one-pot reaction adapted from literature procedures.[2,3] Inside of a glovebox, P^{t}Bu$_3$ (4.62 g, 23 mmol, 1 equiv.) was dissolved in 60 mL of toluene and transferred to a Schlenk flask equipped with a PTFE stir bar. TMSN$_3$ (6 mL, 59 mmol, 2.6 equiv.) was added to the reaction vessel, generating small amounts of a fine white precipitate. The flask was cycled on to a Schlenk line, then brought to reflux and stirred for 6 h. The volatiles were removed *in vacuo* to yield a colorless white solid. Methanol (20 mL, 490 mmol, 21 equiv.) and 1 drop of concentrated H_2SO_4 were added to the flask, then the reaction was stirred at 50°C for 4 d. The volatiles were removed *in vacuo* to give the crude product as a waxy yellow solid that was isolated under ambient atmosphere. The crude product was sublimed at 30 °C (10 mtorr) to yield the title compound as an air-stable, colorless crystalline solid (4.64 g, 93%). The spectroscopic data match the literature values. ^{31}P{^{1}H} NMR (202 MHz, C_6D_6) δ 56.11, which is consistent with the previous report.[3] ^{1}H NMR (500 MHz, C_6D_6) δ 1.22 (d, J = 11.9 Hz, 27H), -0.25 (s, 1H). ^{1}H NMR (400 MHz, C_4D_8O) δ 1.34 (d, *J* = 11.8 Hz, 27H), -0.56 (s, 1H). ^{31}P{^{1}H} NMR (162 MHz, C_4D_8O) δ 55.49.

[Ce^{4+}I(NP(^{t}Bu)$_3$)$_3$], 1-CeI. Inside of a glovebox, HNP(^{t}Bu)$_3$ (272 mg, 1.25 mmol) was dissolved in 2 mL Et_2O in a 20 mL vial. In another vial, potassium benzyl (163 mg, 1.25 mmol) was massed and 2 mL Et_2O was added, and the vial was charged with a glass stir bar. To this slurry, the solution of HNP(^{t}Bu)$_3$ was added, and the reaction mixture was stirred for 10 mins, yielding a white precipitate. In another vial, 2 mL Et_2O was added to CeI$_3$(THF)$_4$ (405 mg, 0.50 mmol) and a glass stir bar. The slurry containing HNP(^{t}Bu)$_3$ and KBn was then added the slurry of CeI$_3$(THF)$_4$, and the residual solids were transferred with a total of 4 mL Et_2O. The combined reaction mixture was stirred in dark, to avoid interference from light, for 48 hours. A bright yellow slurry formed. The reaction mixture was filtered through a 15 mL fine frit packed with Celite, followed by washing of the Celite cake with 15 mL Et_2O. The yellow solution was concentrated *in vacuo* to 8 mL. To this solution, AgI (117 mg, 0.50 mmol) was added as a solid, with the remaining solid transferred using a total of 2 mL Et_2O. Upon addition of AgI, an immediate color change from yellow to brown was observed. The brown mixture was stirred in the dark for 16 h. The mixture was then filtered through a 15 mL fine porosity frit packed with Celite and yielded a red filtrate solution. The filter cake was washed with 20 mL of Et_2O, followed by 10 mL toluene at which point the filtrate was colorless. The combined filtrate was reduced to a solid residue *in vacuo*, and the resulting crude red powder (342 mg) was washed with 3 mL of cold pentane. Upon removal of volatiles *in vacuo*, **1-CeI** was obtained as an orange-red solid (164 mg, 44 %). SC-XRD quality crystals were obtained from a concentrated toluene solution stored at -35 °C for 16 h. ^{1}H NMR (500 MHz, THF) δ 1.46 (d, J = 12.2 Hz, 81H). ^{13}C{^{1}H} NMR (126 MHz, C_4D_8O) δ 42.29, 30.38. ^{31}P{^{1}H} NMR (203 MHz, C_4D_8O) δ 14.89. IR: 2993(w), 2954(m), 2895(m), 2193(w), 2152(w), 1472(m), 1388(m), 1354(m), 1182(m), 1150(m), 1108(m), 1039(s), 1010(s), 961(m), 936(m), 804(m), 619(s), 495(m), 433(w). Elemental analysis % found(calculated) for: C 46.98(47.20), H 8.70(8.91), N 4.59(4.41).

[Ce^{4+}(NP(^{t}Bu)$_3$)$_3$CH$_2$(C$_6$H$_5$)], 2-CeBn. Inside of a glovebox, 3 mL Et_2O was added to a 20 mL scintillation vial charged with **1-CeI** (144 mg, 0.16 mmol) and a glass stir bar. **1-CeI** was not fully dissolved. In another 4 mL vial, potassium benzyl (22 mg, 0.17 mmol) was slurried in 1 mL Et_2O. Both vials were cooled in the cold well (using liquid nitrogen). The potassium benzyl slurry was added to the **1-CeI** slurry via pipette. The color of the reaction mixture immediately changed from red to dark green. The reaction mixture was stirred for 1 h inside of the cold well, where the temperature was maintained at approximately between -30 to -60 °C. Subsequent cold filtration through glass pipette packed with celite and glass fiber yielded a dark green solution. The solution was slowly warmed up to room temperature, followed by removal of volatiles *in vacuo*, leaving a dark green powder. The resulting dark green powder was triturated with pentane (3 x 1 mL), followed by extraction in 6 mL pentane. The pentane solution was filtered through pipette filter packed with Celite, and concentrated *in vacuo*. The concentrated pentane solution in a 4 mL vial was placed in a 20 mL vial with 5mL toluene in the outer vial (in order to induce pentane evaporation) and the vial placed in -35 °C freezer. Overnight, dark green crystals formed. The dark green crystals were decanted and dried *in vacuo*

to yield a dark green powder (88 mg, 63 %). Recrystallization from HMDSO at -35 °C yielded SC-XRD quality crystals. ^{1}H NMR (500 MHz, C_6D_6) δ 7.35 (s, 2H), 7.22 (d, J = 6.8 Hz, 2H), 6.49 – 6.42 (m, 1H), 2.43 (s, 2H), 1.41 (d, J = 12.1 Hz, 81H). $^{13}C\{^{1}H\}$ NMR (126 MHz, C_6D_6) δ 152.72, 123.75, 117.14, 90.12, 41.78, 41.43, 30.29. $^{31}P\{^{1}H\}$ NMR (202 MHz, C_6D_6) δ 14.26. IR: 2993.34(w), 2956.40(m), 2867.17(m), 1588.11(w), 1472.08(w), 1444.26(m), 1385.57(m), 1355.91(m), 1296.41(w), 1186.28(m), 1141.96, 1112.40(m), 1042.32(s), 950.01(m), 932.76(m), 862.99(m), 803.59(w), 756.82(m), 710.97(m), 690.88(m), 613.59(s), 525.39(m), 493.56(m), 431.40(m). Elemental analysis % found(calculated) for: C 58.88(58.67), H 10.23(10.08), N 4.67 (4.77).

Synthesis of [Ce^{4+}($NP(^{t}Bu)_3)_3CH_2C(CH_3)_3$] • 0.3 [$O(Si(CH_3)_3)_2$], 2-CeNpt. Inside of the glovebox, **1-CeI** (219 mg, 0.24 mmol) was slurried in 3 mL Et_2O in a 20 mL scintillation vial charged with a glass stir bar. In another 20 mL scintillation vial, neopentyllithium (20 mg, 0.25 mmol) was dissolved in 1mL Et_2O. Both vials were cooled in the cold well (using liquid nitrogen). The neopentyllithium solution was added to the **1-CeI** solution via pipette and the solution colored turned black. The reaction mixture was stirred in the cold well for 1 hr, maintaining the temperature of approximately between -30 and -60 °C. The reaction mixture was then filtered through a glass pipette filter packed with celite, pre-cooled in the cold well prior to use. The filtrate was warmed to room temperature gradually and volatiles were then removed *in vacuo* to yield a black, sticky residue. The residue was dissolved in 3 mL of HMDSO, filtered through a glass pipette filter packed with celite, concentrated *in vacuo*, and then cooled to -35 °C. After 16 h, colorless solids formed and the solution was decanted. The decantate was filtered, concentrated *in vacuo*, and cooled for another 16 h to yield **2-CeNpt** as black crystals (50.9 mg, 25 %) upon drying *in vacuo*. The bulk product contains 0.3 equivalents of HMDSO (after 1 h *in vacuo*), as determined from NMR and EA. ^{1}H NMR spectrum shows a peak corresponding to HMDSO which integrates to 6 protons indicating that there are 0.3 equivalents of HMDSO relative to **2-CeNpt** in bulk sample. SC-XRD quality crystals were obtained directly from approx. half a scale reaction (starting with **1-CeI**, 0.11mmol) in 1 mL HMDSO with 10 drops of pentane, filtered through a glass pipette filter packed with celite, further concentrated *in vacuo* to ~0.8 mL and cooled at -35 °C for 16 h. ^{1}H NMR (400 MHz,C_7D_8) δ 1.77 (s, 9H), 1.40 (t, J = 10.4 Hz, 83H). Peak at 1.40 consists of a singlet at 1.43(2H) and a doublet 1.40-1.37(81H). $^{13}C\{^{1}H\}$ NMR (101 MHz, C_7D_8) δ 107.85, 41.67, 41.22, 37.88, 36.81, 30.27. $^{31}P\{^{1}H\}$ NMR (162 MHz, C_7D_8) δ 13.78. IR: 2996.37(w), 2953.75(w), 2886.34(m), 2333.02(w), 1471.69(w), 1448.75(m), 1386.22(w), 1352.16(m), 1252.22(w), 1186.30(w), 1150.01(w), 1102.53(m), 1052.17(s), 1016.67(s), 961.51(m), 840.65(m), 803.16(m), 782.64(m), 755.70(w), 613.84(m), 494.88(w). Elemental analysis % found(calculated) for C 55.41 (56.48), H 10.27 (10.80), N 4.53 (4.60).

NMR Spectroscopy

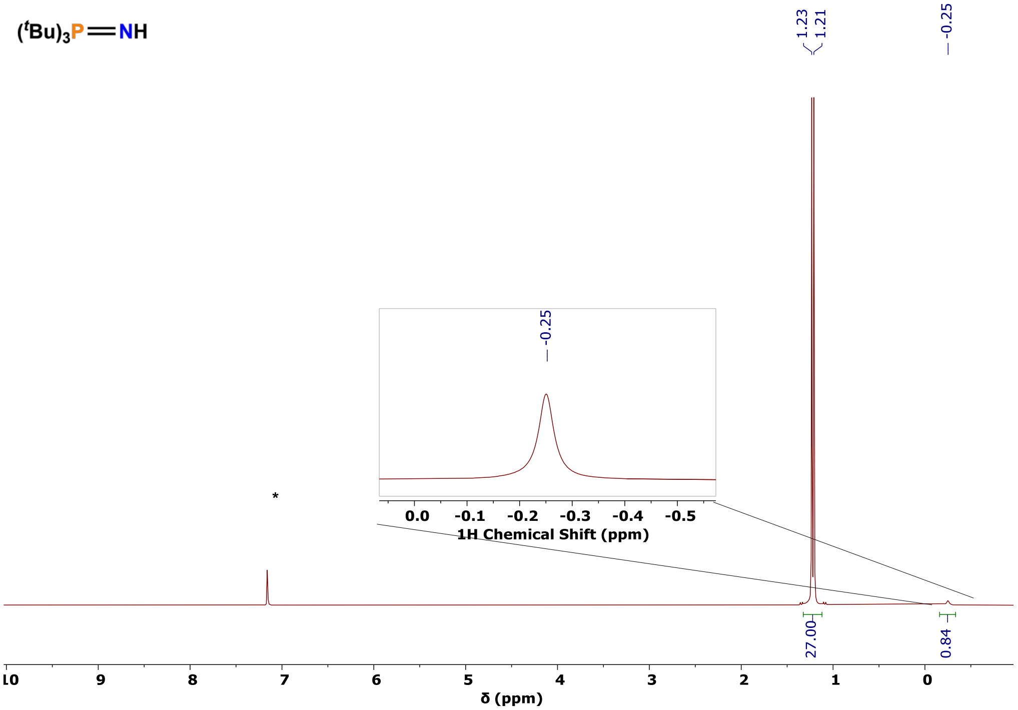

Figure S1. ^{1}H NMR of HNP(^{t}Bu)$_3$ in C_6D_6. Residual solvent (C_6D_5H) is denoted as *.

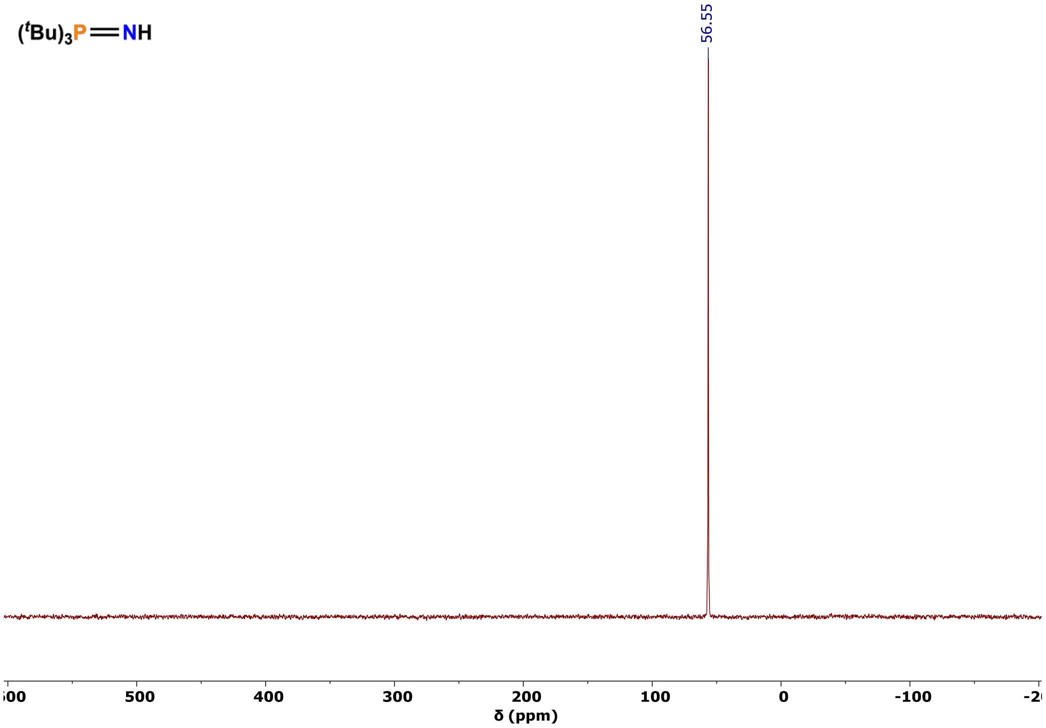

Figure S2. ^{31}P{^{1}H} NMR of HNP(^{t}Bu)$_3$ in C_6D_6.

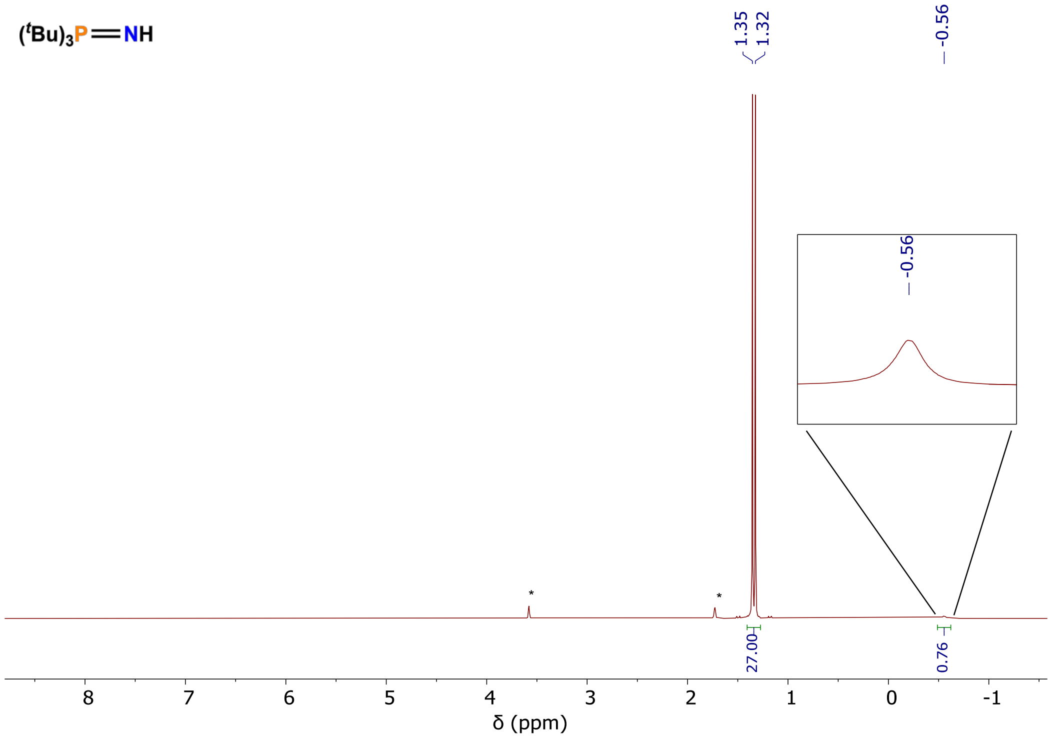

Figure S3. ${}^{1}H$ NMR of HNP(^{t}Bu)$_3$ in C_4D_8O. Residual solvent (C_4D_7HO) is denoted as *.

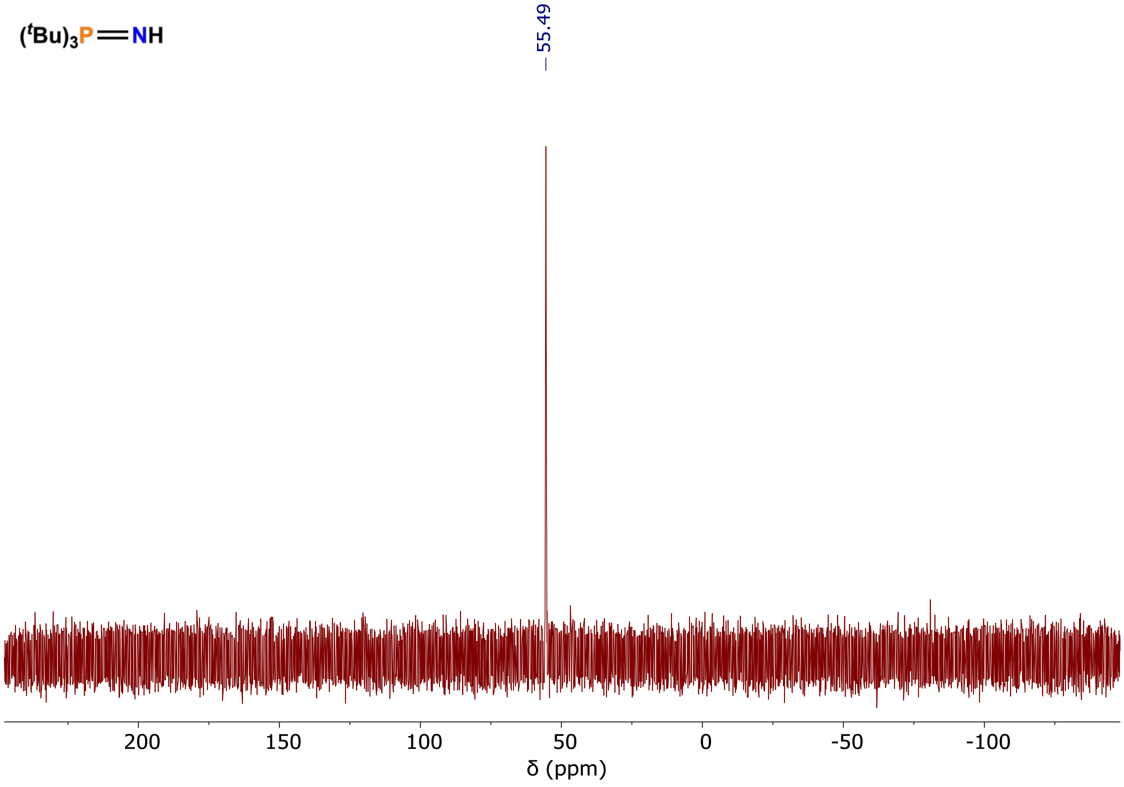

Figure S4. ${}^{31}P\{{}^{1}H\}$ NMR of HNP(^{t}Bu)$_3$ in C_4D_8O.

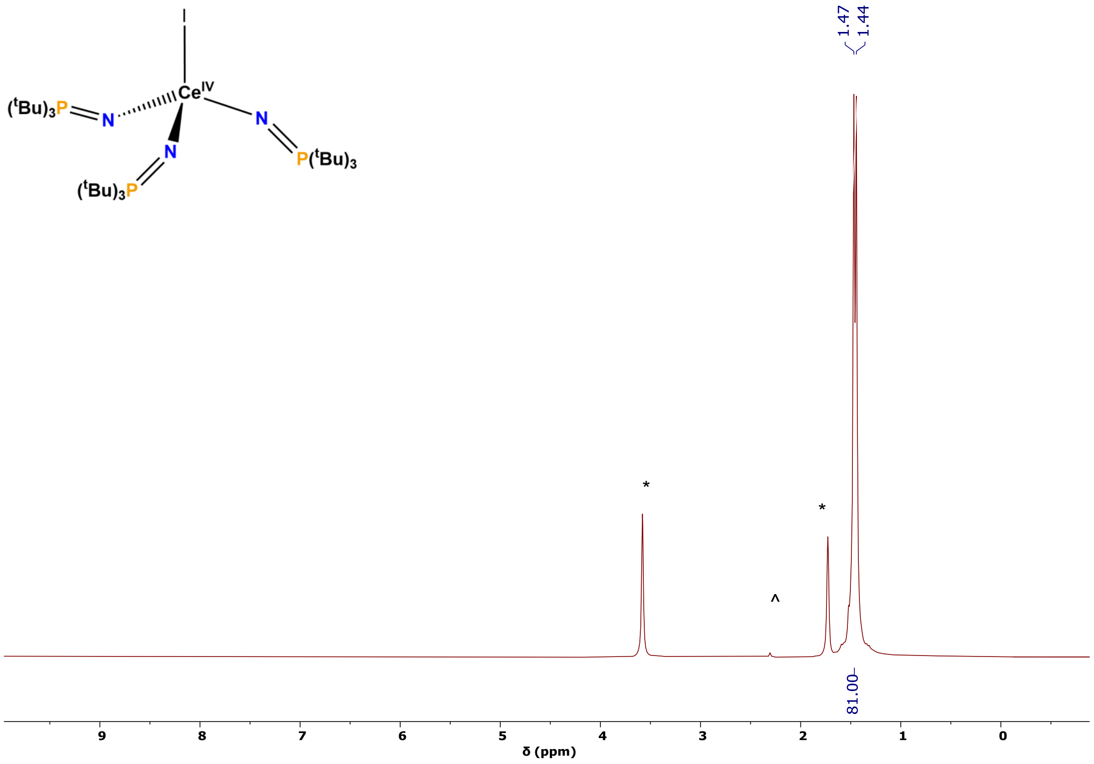

Figure S5. ^{1}H NMR of **1-CeI** in C_4D_8O. Residual solvent (C_4D_7HO) is denoted as *, residual toluene is shown as ^.

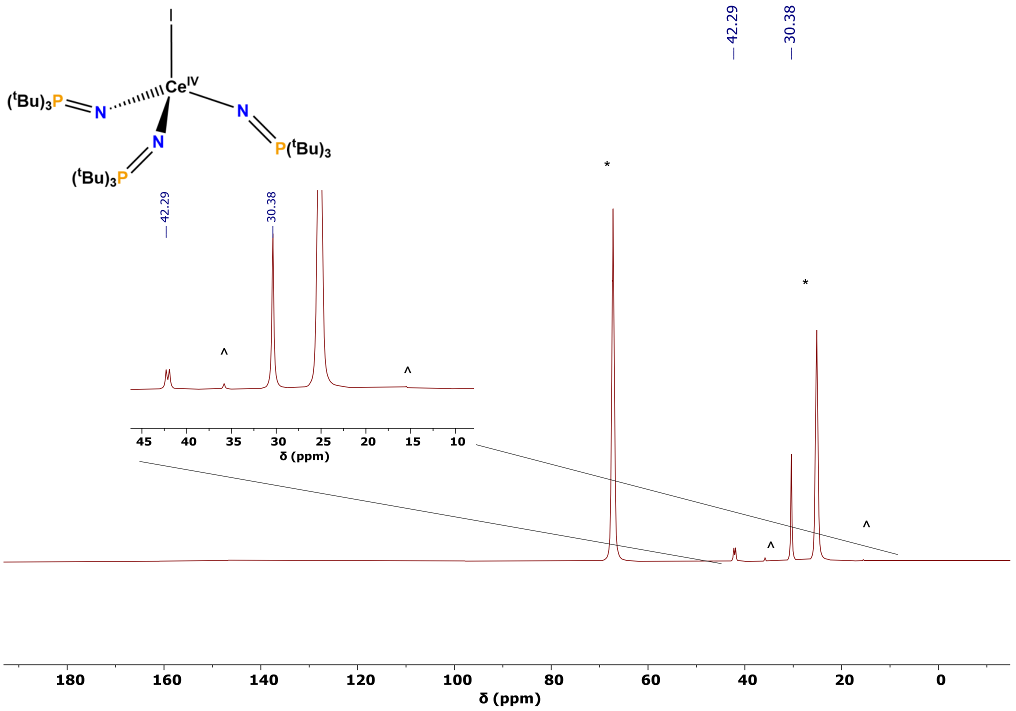

Figure S6. $^{13}C\{^{1}H\}$ NMR of **1-CeI** in C_4D_8O. Residual solvent (C_4D_7HO) is denoted as *, residual pentane is shown as ^.

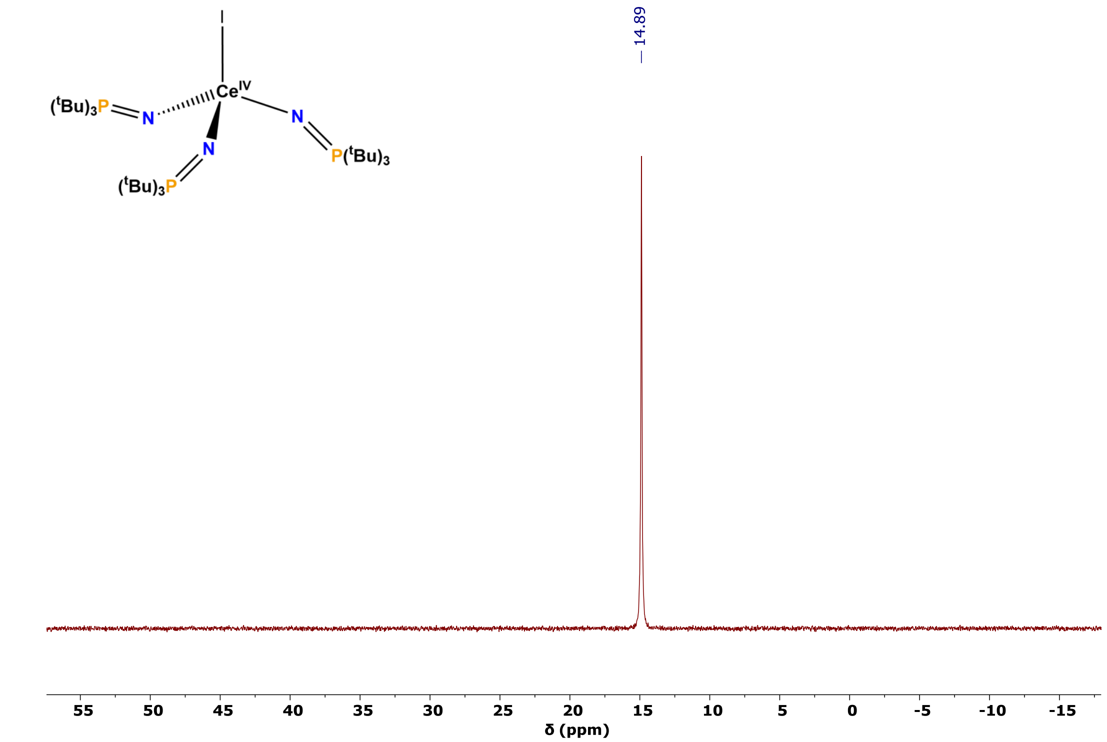

Figure S7. $^{31}P\{^{1}H\}$ NMR of **1-CeI** in C_4H_8O.

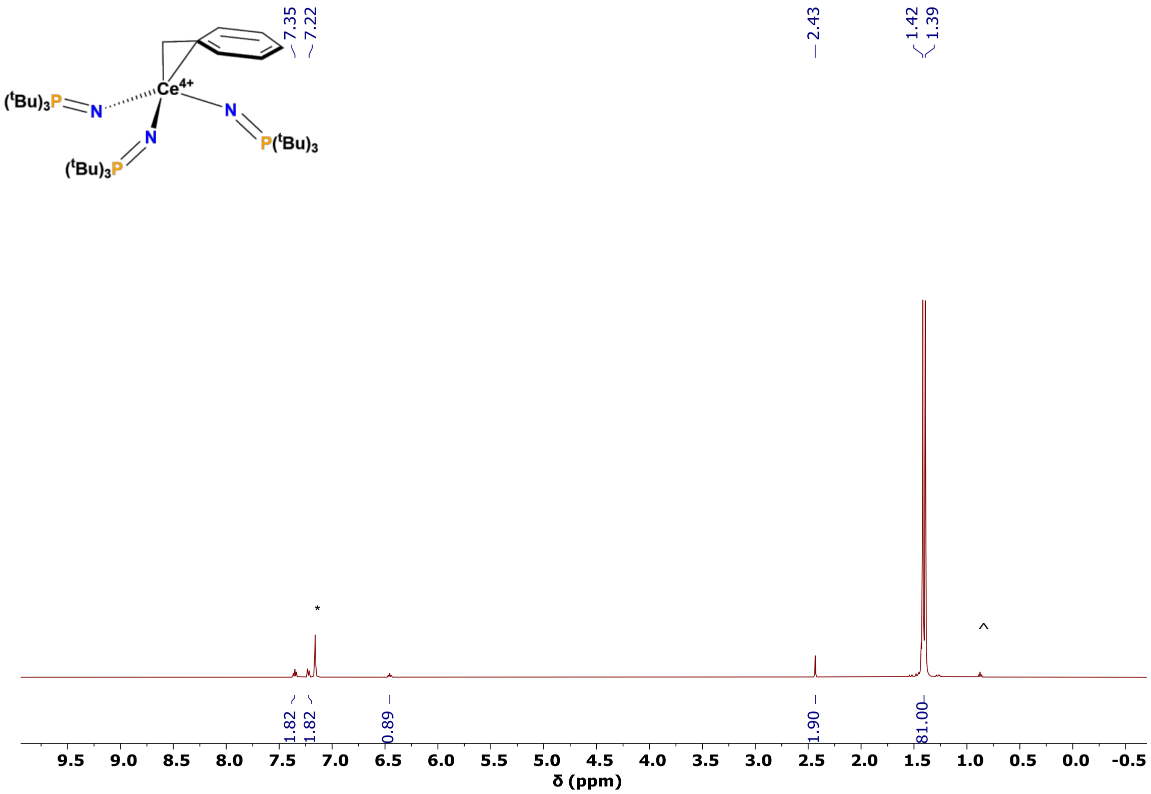

Figure S8. ^{1}H NMR of **2-CeBn** in C_6D_6. Residual solvent (C_6D_5H) is denoted as *, residual pentane is shown as ^.

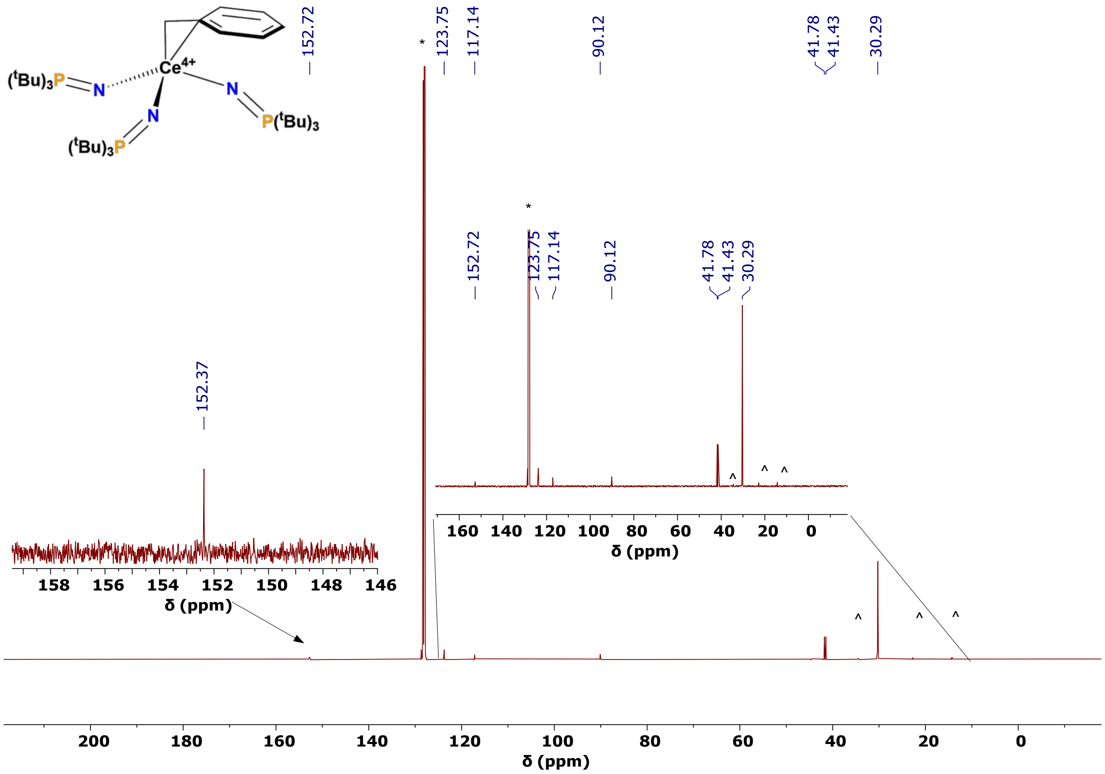

Figure S9. $^{13}C\{^{1}H\}$ NMR of **2-CeBn** in C_6D_6. Residual solvent (C_6D_5H) is denoted as *, residual pentane is shown as ^.

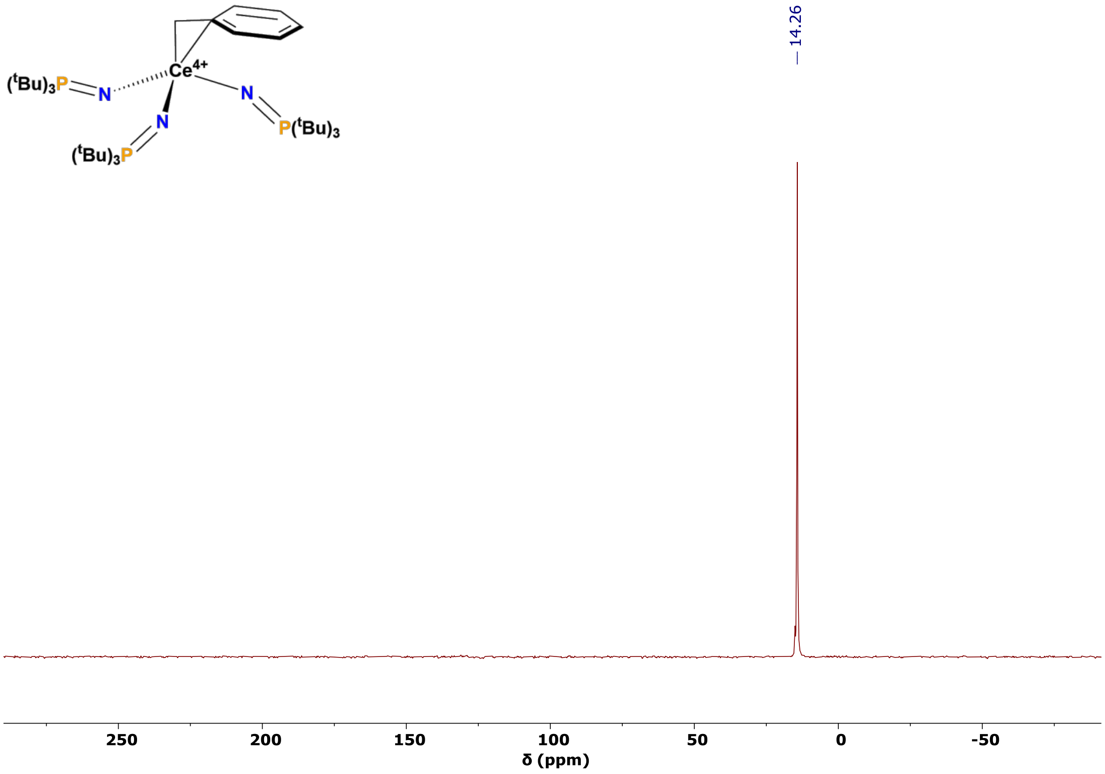

Figure S10. $^{31}P\{^{1}H\}$ NMR of **2-CeBn** in C_6D_6.

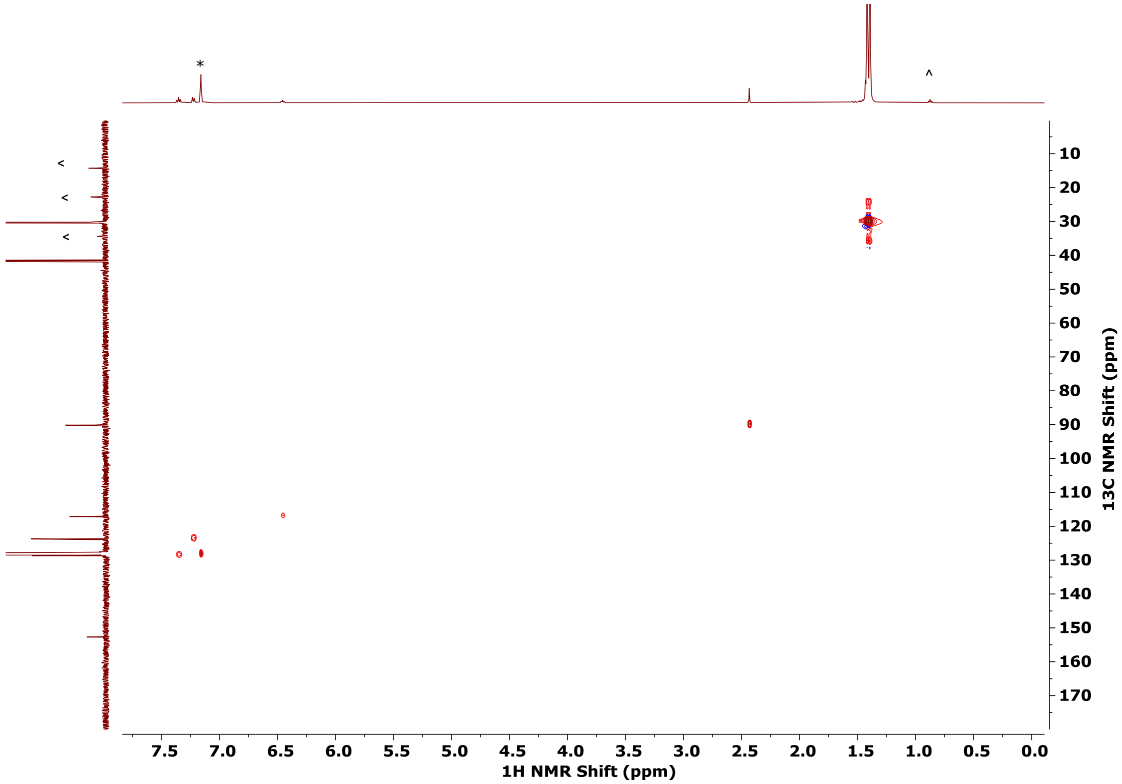

Figure S11. ^{1}H-^{13}C HSQC NMR of **2-CeBn** in C_6D_6.

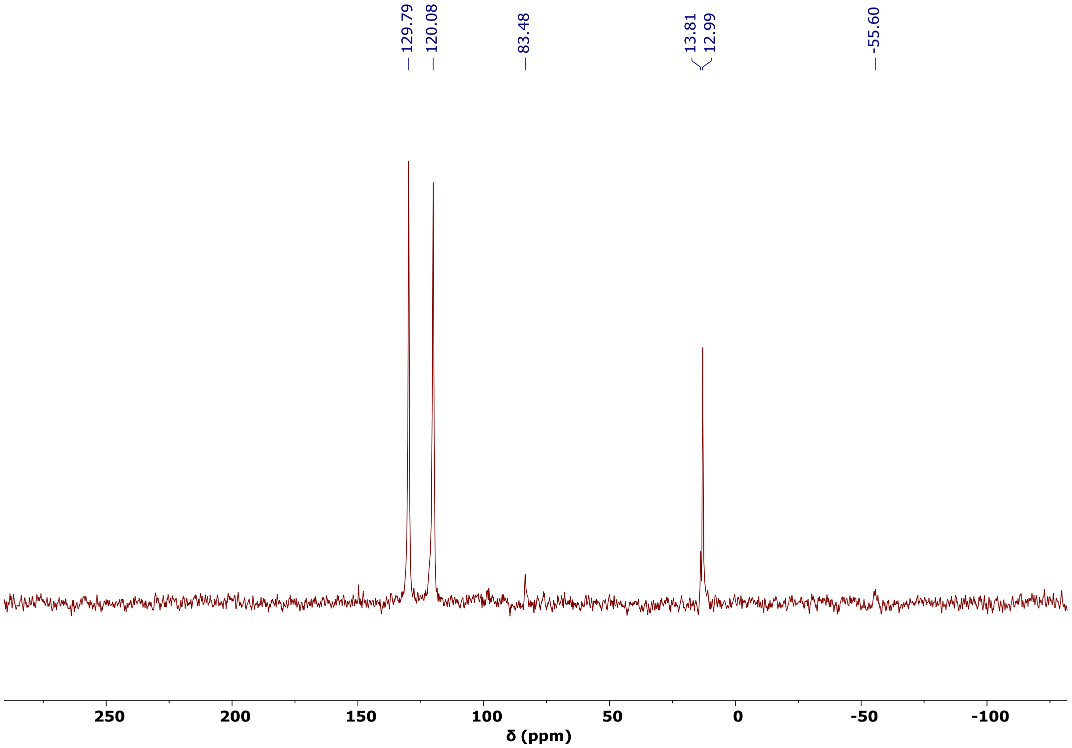

Figure S12. $^{31}P\{^{1}H\}$ NMR of **2-CeBn** synthesis when THF is used in place of Et_2O. The reaction immediately turns to dark green, however within 10 mins the reaction color bleaches to yellow. The mixture was dried *in vacuo* and the redissolved in C_6D_6 for comparison, showing that it incurs multiple products that do not match with **2-CeBn.**

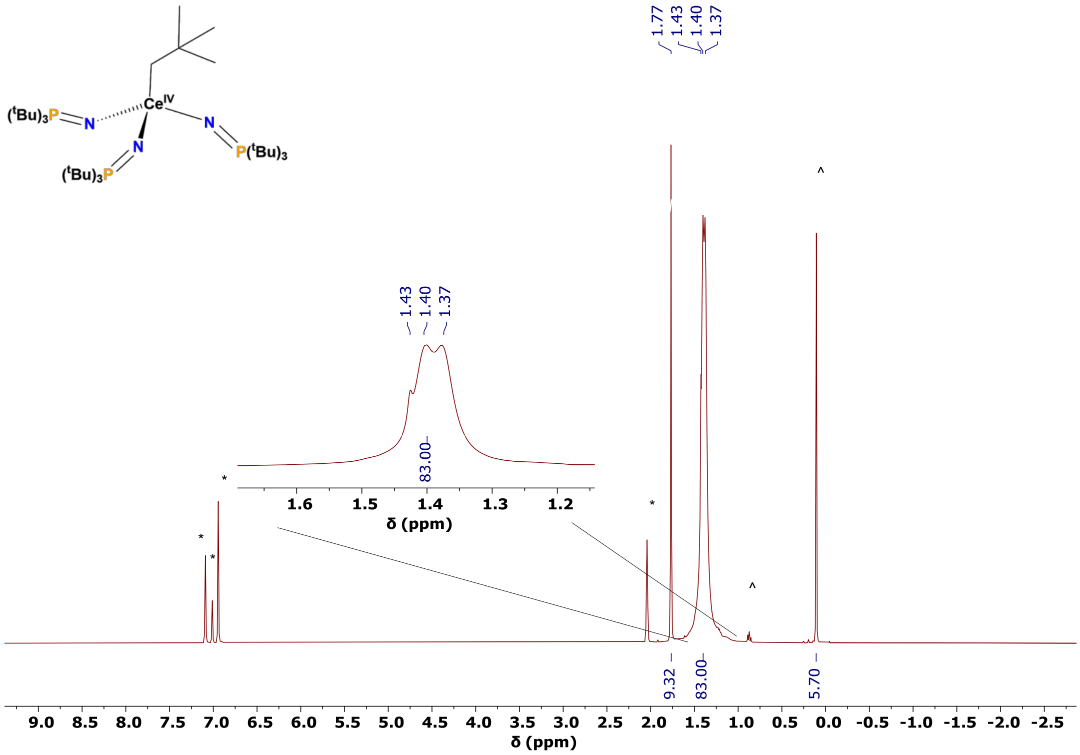

Figure S13. ^{1}H NMR of **2-CeNpt** in C_7D_8 at 238 K. Residual solvent (C_7D_7H) is denoted as *, HMDSO is shown as #, and residual pentane is shown as ^. Peak at 1.40 consists of a singlet at 1.43 (2H) and a doublet 1.40-1.37 (81H); this assignment is further confirmed by ^{1}H-^{13}C HSQC NMR.

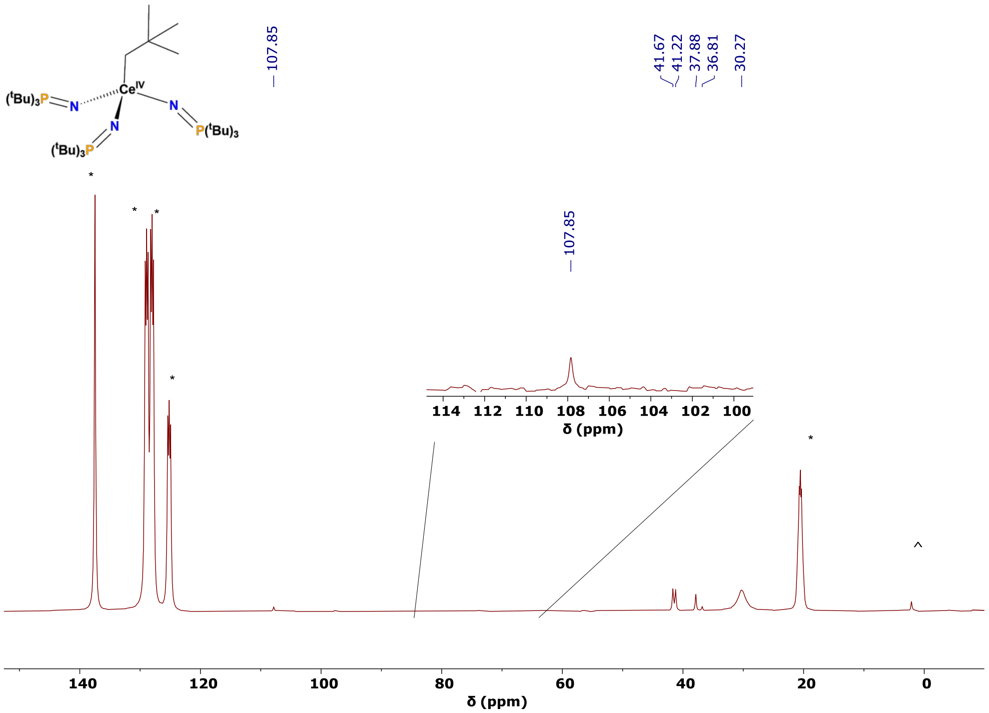

Figure S14. ^{13}C{^{1}H} NMR of **2-CeNpt** in C_7D_8 at 238 K. Residual solvent (C_7D_7H) is denoted as *, HMDSO is shown as ^.

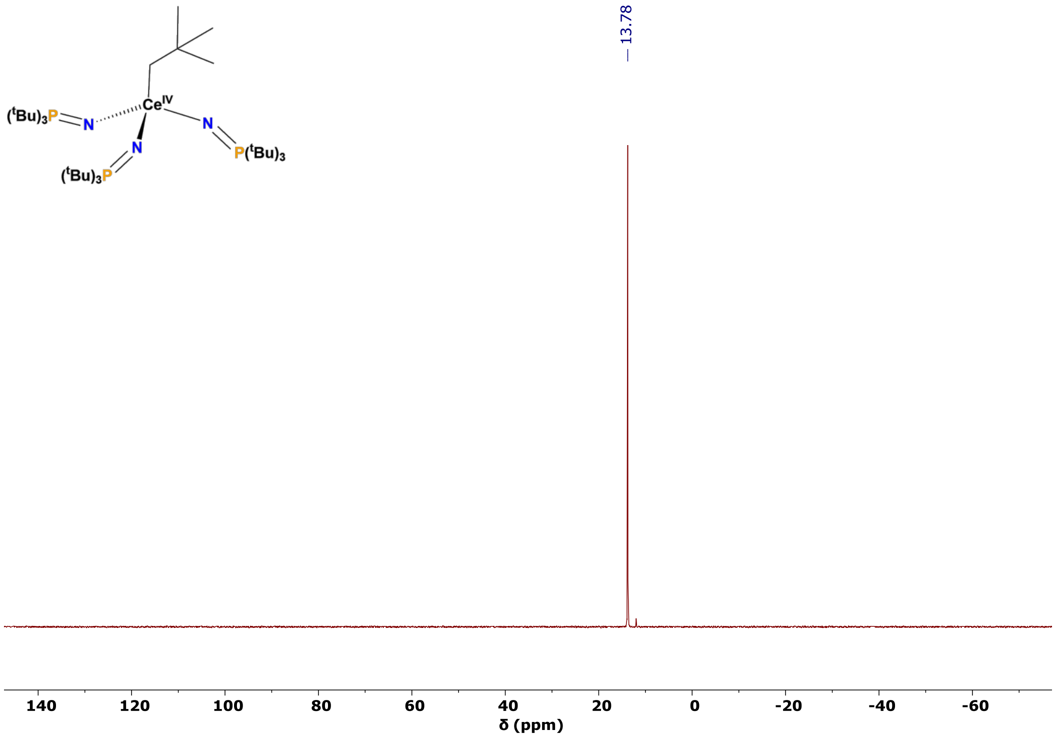

Figure S15. $^{31}P\{^{1}H\}$ NMR of **2-CeNpt** in C_7D_8 at 238 K.

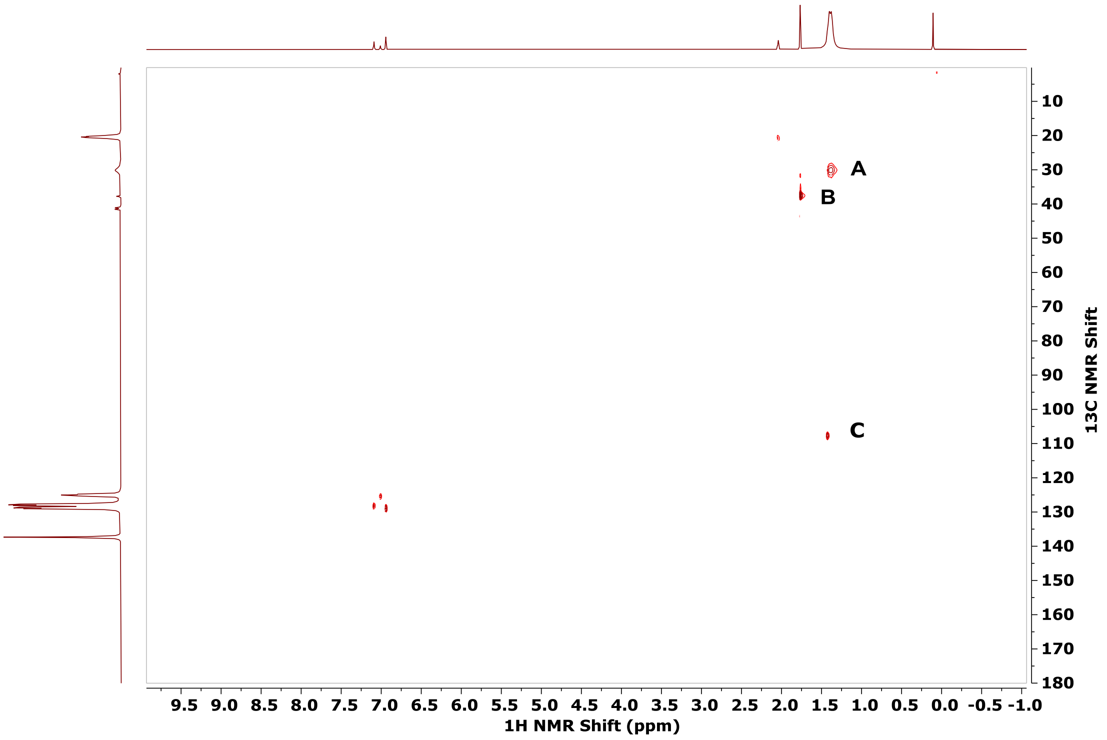

Figure S16. ^{1}H-^{13}C HSQC NMR of **2-CeNpt** in C_7D_8 at 238 K. Cross-peaks marked as A, B, C are assigned in the following figure (**Figure S17**). Residual solvent (C_7D_7H) is denoted as *, HMDSO is shown as ^.

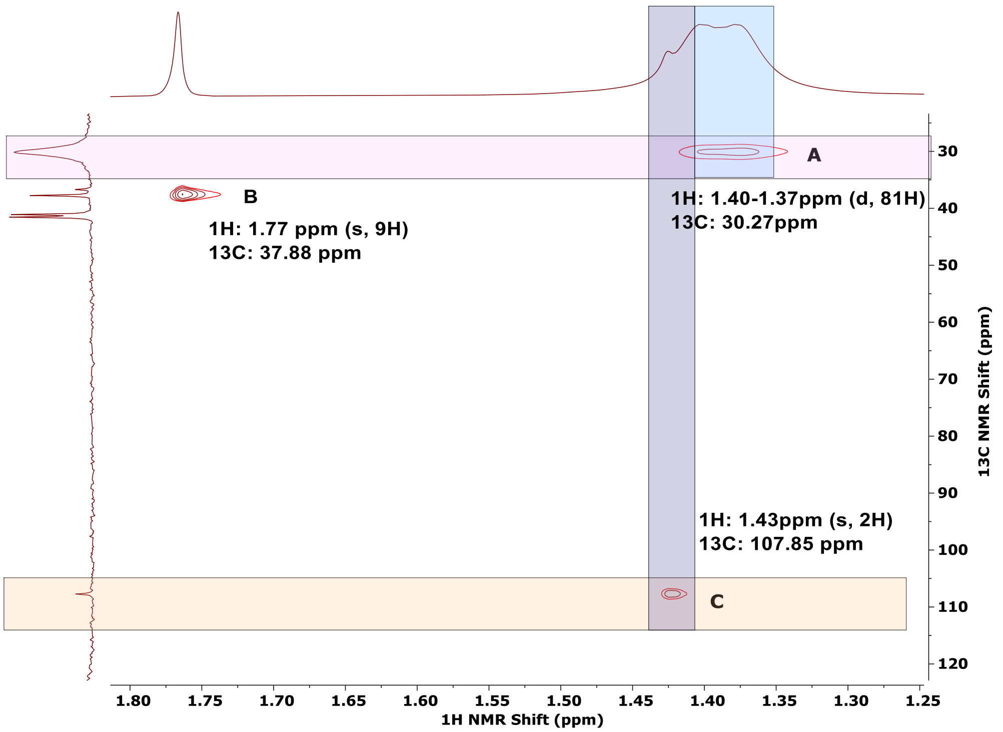

Figure S17. Magnified and annotated ^{1}H-^{13}C HSQC NMR spectrum in C_7D_8 (**Figure S16**) at 238 K, highlighting the coincident proton peaks from 1.43-1.37 ppm.

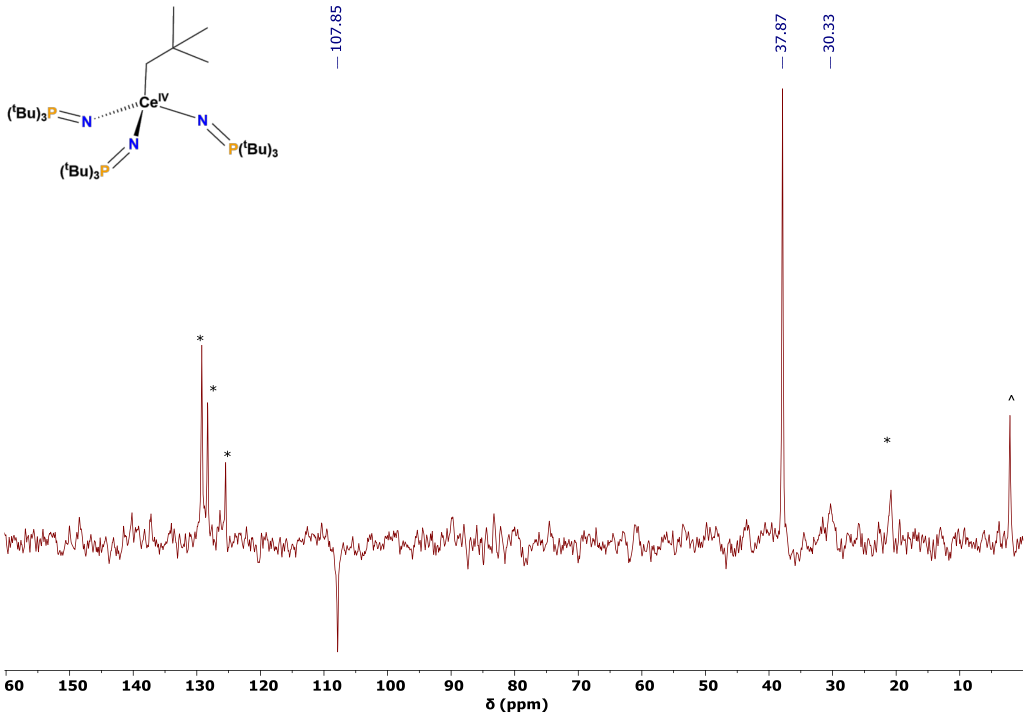

Figure S18. ^{13}C{^{1}H} DEPT-135 NMR of **2-CeNpt** at 238 K in C_7D_8. Residual solvent signals are shown as *, HMDSO is shown as ^.

Thermal Stability Study by NMR

1-CeI was dissolved in C_4D_8O and **2-CeBn** was dissolved in C_7D_8 and transferred to a NMR tube fitted with a J. Young tap. Then the NMR-tube was inserted into the spectrometer at room temperature and warmed to target temperatures incrementally over the temperature range of 30°C to 45°C(**1-CeI**) and 80°C(**2-CeBn**). Samples were held at target temperature for 10 mins, where the $^{31}P\{^1H\}$ and ^1H NMR spectra were collected. Temperatures were corrected using ethylene glycol calibration curve.

For **1-CeI** and **2-CeBn**, no noticeable decomposition was found at any temperature up to 45 °C (**2-CeI**) and 80 °C (**2-CeBn**). These are the temperature limits of VT-NMR studies under compatible solvent (C_4D_8O for **1-CeI**, C_7D_8 for **2-CeBn**) and the instrument. Therefore, the J-Young tubes with the samples were heated in an oil bath warmed to 60 °C (**1-CeI**) or 100 °C (**2-CeBn**) for 1 hr, cooled down to room temperature and NMR spectra were collected at ambient temperature.

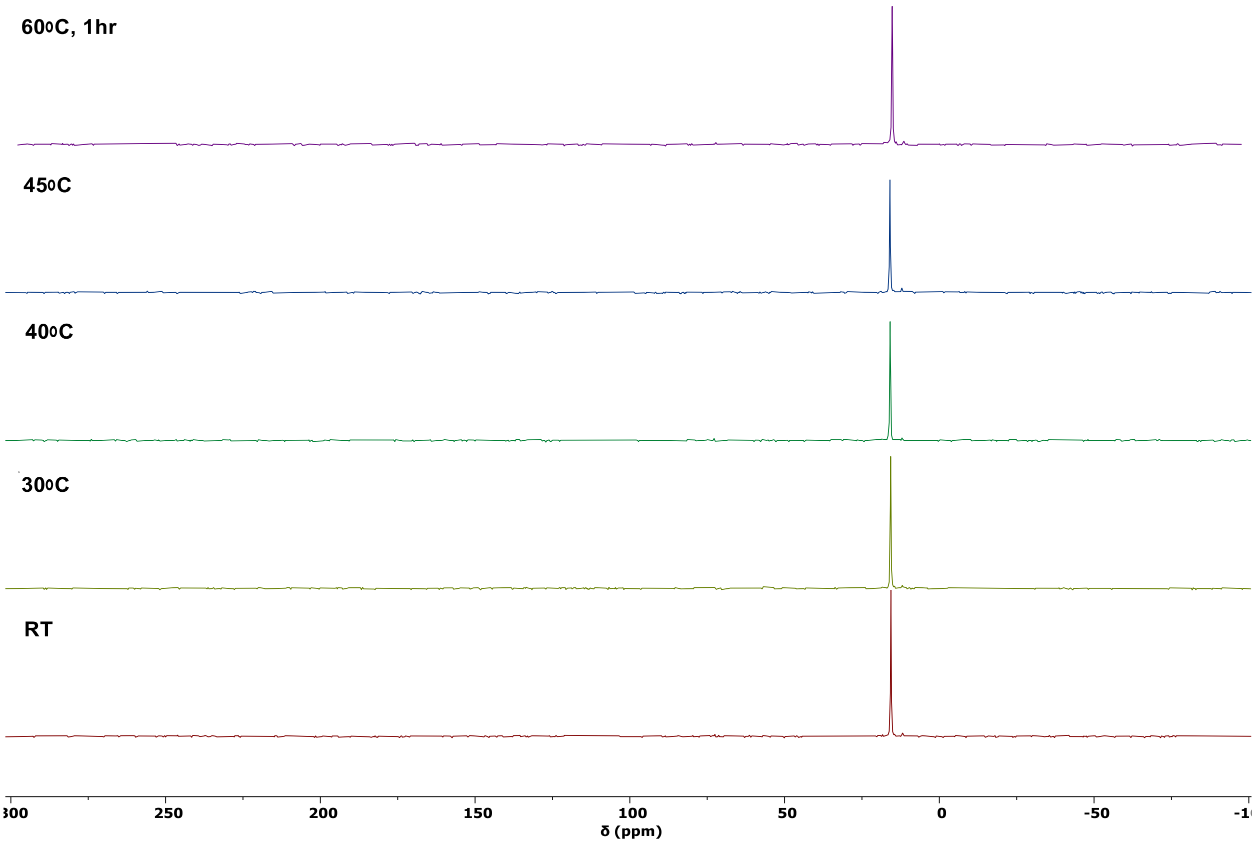

Figure S19. Variable-Temperature $^{31}P\{^1H\}$ NMR (202 MHz) of **1-CeI** in C_4D_8O at ambient temperature (RT, 25 °C), 30 °C, 40 °C, 45 °C overlayed with $^{31}P\{^1H\}$ NMR spectrum (162 MHz) obtained after heating of the sample at 60 °C for 1hr in an oil bath (spectrum acquired at 25 °C). No discernable decomposition of **1-CeI** was observed over this temperature range.

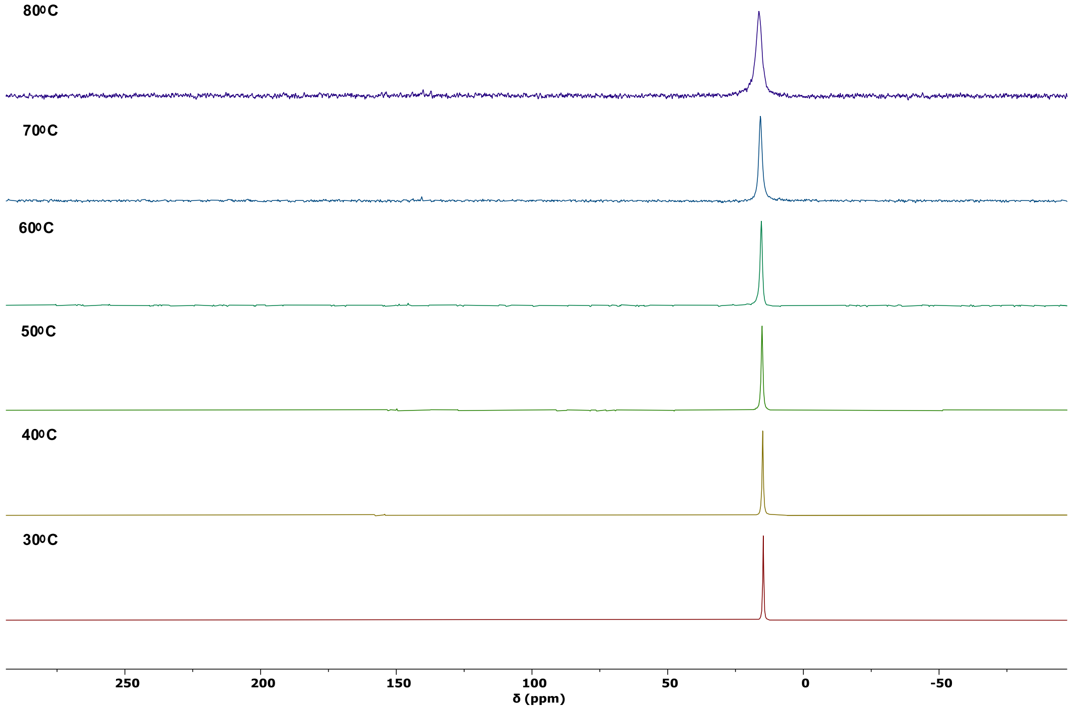

Figure S20. Variable-Temperature $^{31}P\{^{1}H\}$ NMR (202 MHz) of **2-CeBn** in C_7D_8 acquired between 30 °C – 80 °C in 10 °C increments. No discernable decomposition of **2-CeBn** was observed over this temperature range.

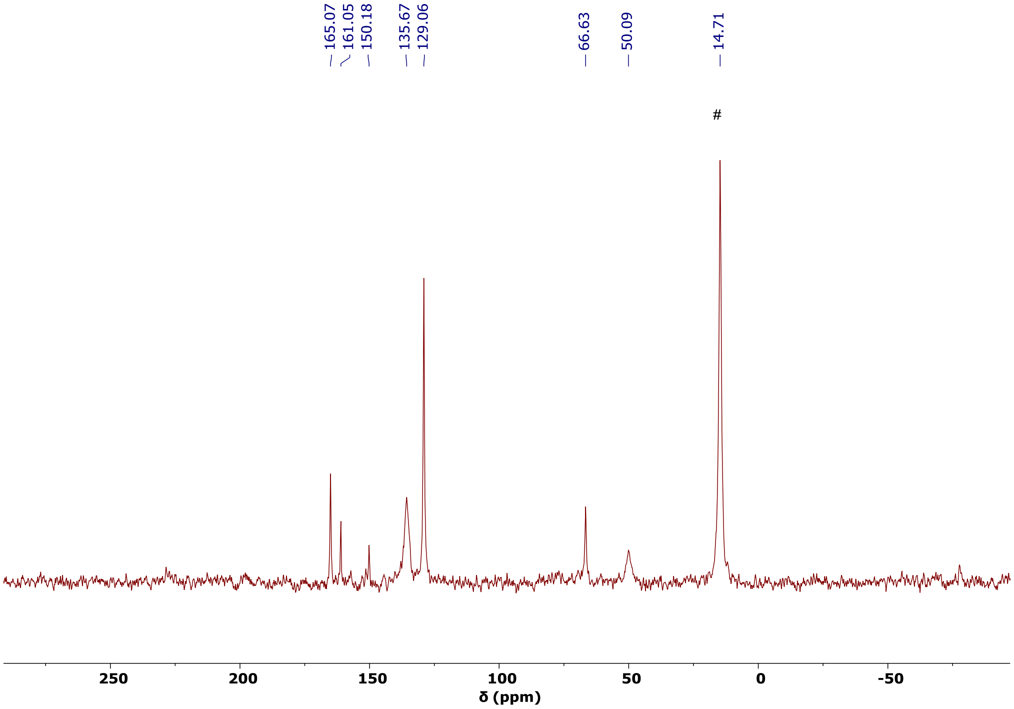

Figure S21. $^{31}P\{^{1}H\}$ NMR (202 MHz) of **2-CeBn** in C_7D_8 after heating in the oil bath at 100 °C for 1h. Spectrum was recorded at ambient temperature. # denotes the peak corresponding to **2-CeBn**. The spectrum shows partial decomposition of **2-CeBn** into 7 resonances which are paramagnetically shifted indicative of the formation of Ce^{3+} species.[8–11]

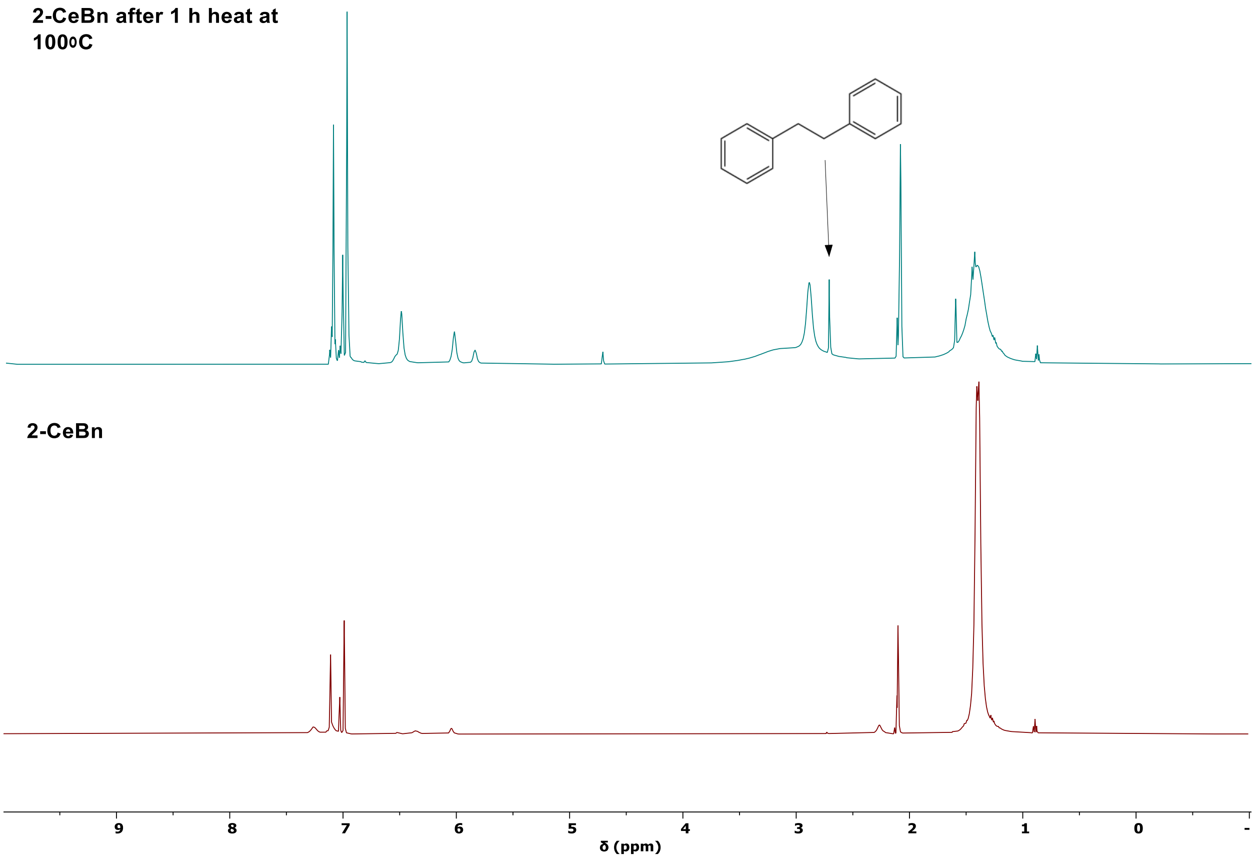

Figure S22. ^{1}H NMR (500 MHz) of **2-CeBn** heated in C_7D_8 at 100 °C for 1hr overlaid with **2-CeBn** (500 Hz) in C_7D_8 at 25°C. Upon heating, a peak corresponding to dibenzyl at 2.71 ppm[12] is observed.

Electrochemistry

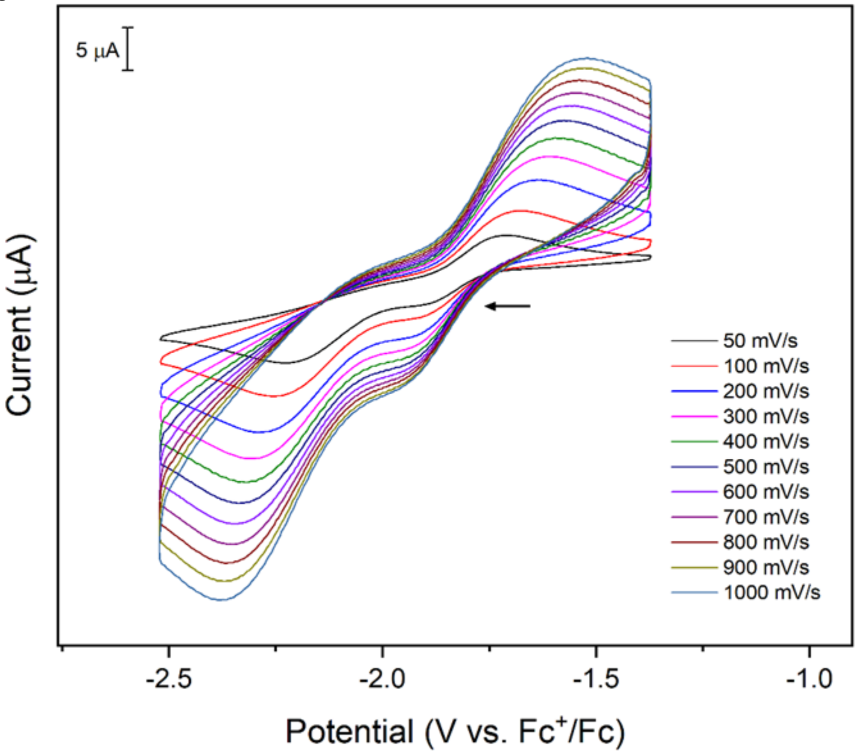

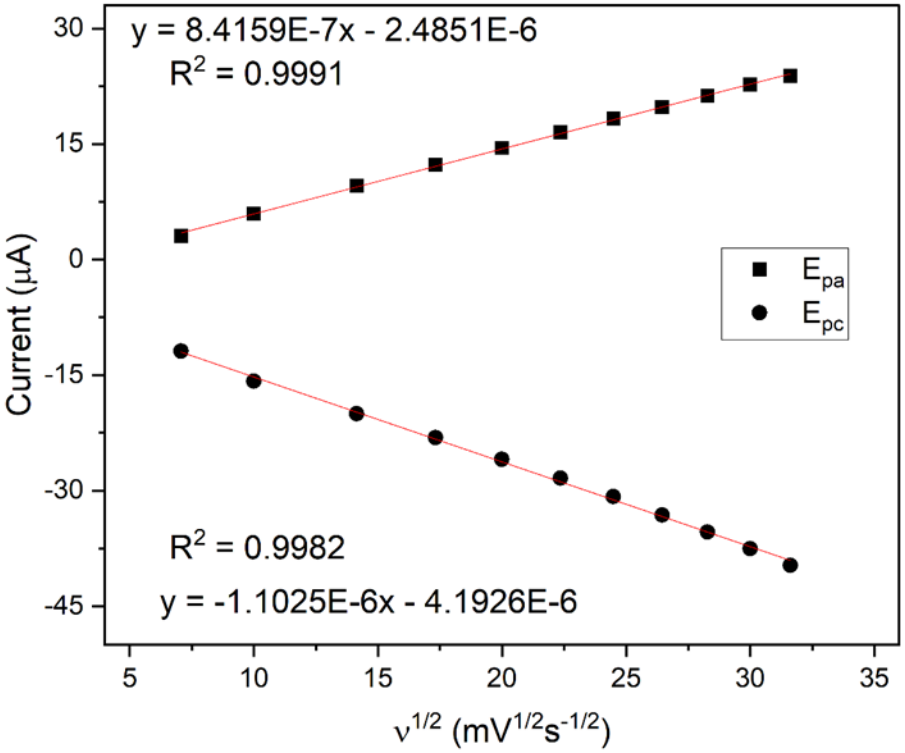

Figure S23. Scan-rate dependence of cyclic voltammogram of 3 mM **1-CeI** in 100 mM [^{n}Bu$_4$N][BPh$_4$] in THF (top) and Randles-Sevcik plot (bottom).

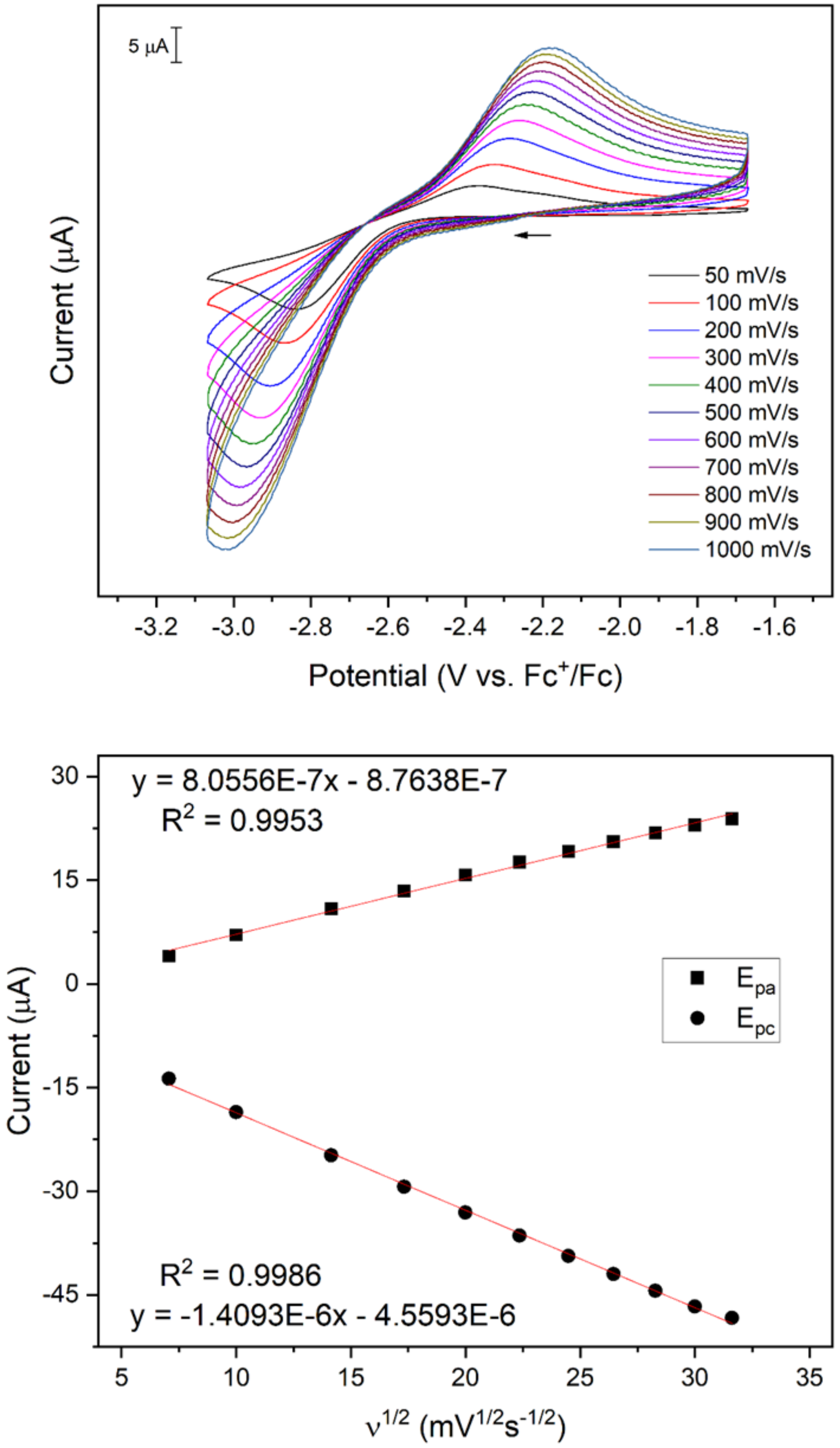

Figure S24. Scan-rate dependence of cyclic voltammogram of 3 mM **2-CeNpt** in 200 mM [^{n}Bu$_4$N][PF$_6$] in PhF (top) and Randles-Sevcik plot (bottom).

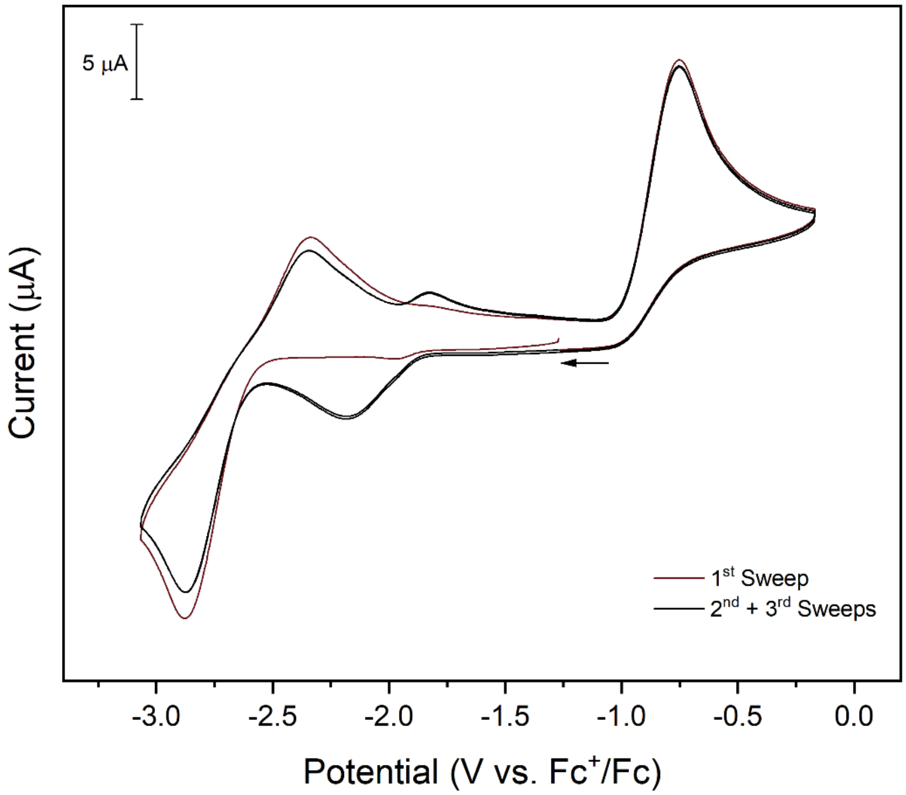

Figure S25. Consecutive sweeps of cyclic voltammogram of **2-CeNpt** in 200 mM [^{n}Bu$_4$N][PF$_6$] in PhF.

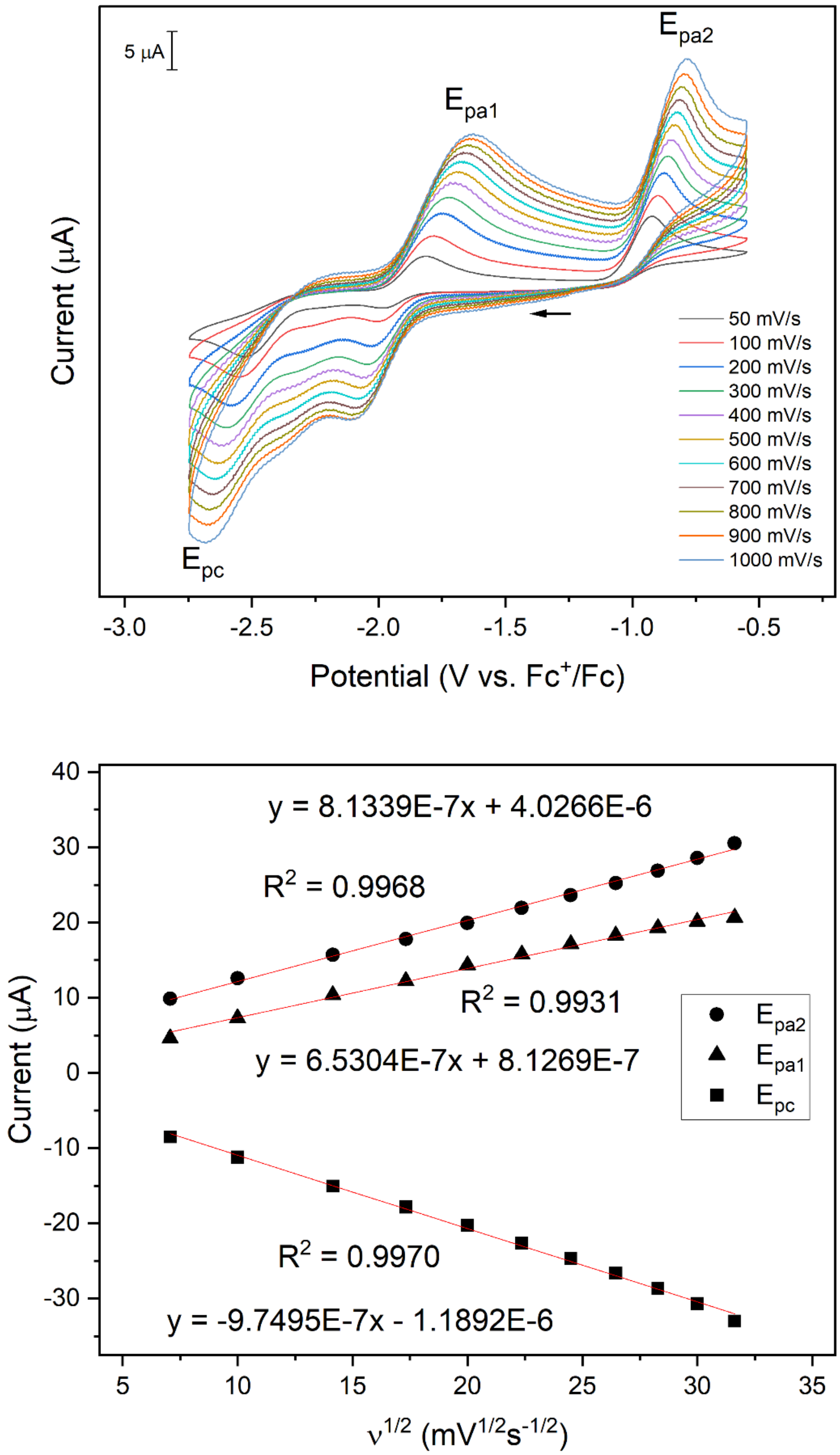

Figure S26. Scan-rate dependence of cyclic voltammogram of **2-CeBn** in 200 mM [$^n Bu_4N$][PF_6] in PhF (top) and Randles-Sevcik plot (bottom).

Cyclic Voltammetry Discussion:

The voltammograms of **1-CeI** and **2-CeBn** display minor, secondary reductive features around -2 V (**Figs. S23, S26**). The current response is higher in magnitude for **2-CeBn** than in **1-CeI**, but visually identifiable in both voltammograms. Bulk purity is confirmed by other means (NMR, elemental analysis), suggesting these features are not due to an impurity. There is no corresponding secondary oxidative feature within the scan window, which is more indicative of solution behavior/speciation and not simply an impurity or degradation. In contrast, the voltammogram of **2-CeNpt** (**Fig. S25**) is suggestive of compound degradation, with the appearance of both oxidative and reductive features after the first cycle. Of note, the features for **1-CeI** and **2-CeBn** appear where E_{pc} could be expected for a quasi-reversible event corresponding to E_{pa} (~200 mV), possibly indicating that a portion of the reduced species in solution is more structurally templated for reduction, and therefore occurs at a milder potential compared to the main E_{pa} features which occur at potentials closer to -2.5 V. The identity and speciation of the complexes in solution cannot be definitively identified, and could consist of mono- and/or dimeric species, with equilibria between them, yielding different reduction potentials. Another possible explanation is incomplete anion ejection upon electrochemical reduction. Ce imidophosphorane complexes often display wide peak separations due to significant structural rearrangement upon electrochemical oxidation/reduction,[8–10] and the large peak-to-peak separation observed herein is consistent to those of related homoleptic complexes. A secondary reduction is not observed in the voltammogram of **2-CeNpt**, however, **2-CeNpt** is not as robust under electrochemical conditions and, after the first scan, anodic and cathodic features appear around -1.8 and -2.3 V, respectively (**Fig. S25**). The enhanced stability of **2-CeBn** compared to **2-CeNpt** could be due to the η_2 coordination of the benzyl fragment. In sum, the stabilizing effect of the Npt^-, Bn^-, and I^- fragments is evaluated, and Npt^- appears to stabilize the tetravalent oxidation state more than Bn^- and I^-, based on the potentials of E_{pc}, with the voltammogram of **2-CeNpt** (**Fig. S25**) resembling homoleptic Ce^{4+} imidophosphorane complexes.[8–10]

UV-vis-NIR Electronic Absorption Spectra

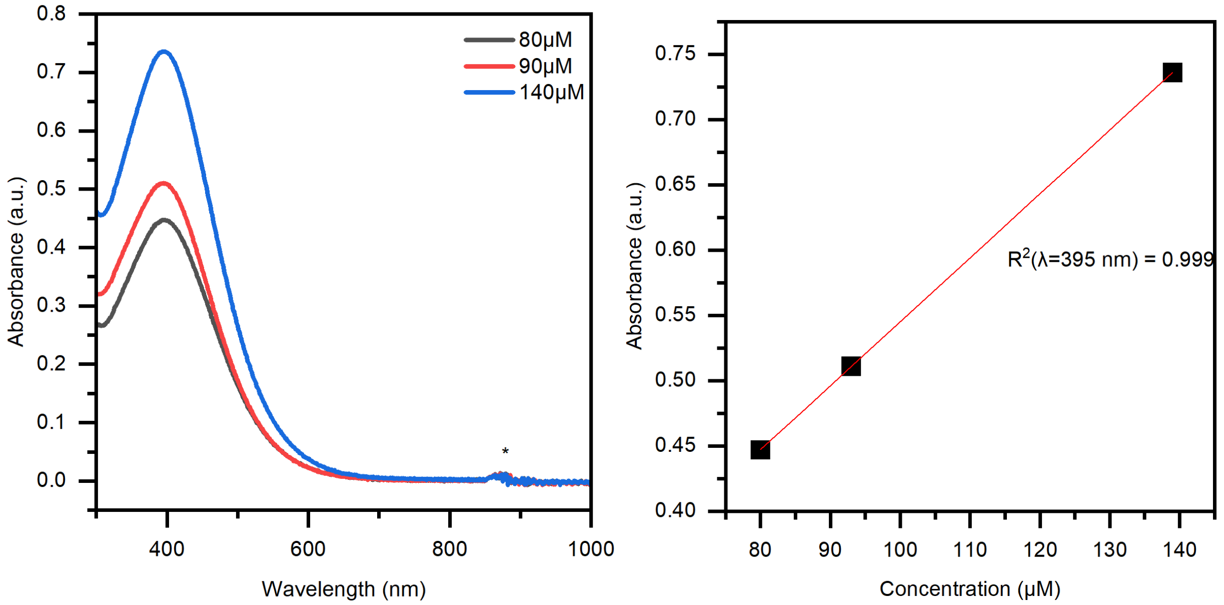

Figure S27. (Left) UV-vis-NIR spectra of **1-CeI** in toluene. (Right) Linear regression on absorbance at λ_{max}= 395 nm. * marks solvent artifact.

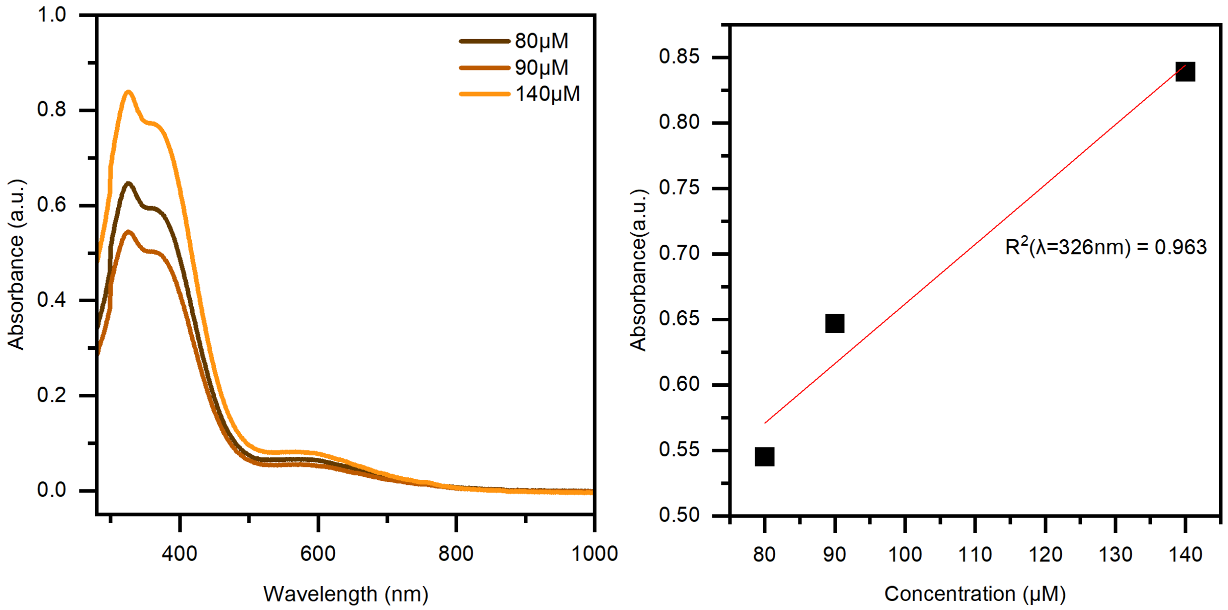

Figure S28. (Left)UV-vis-NIR spectra of **2-CeBn** in C_6H_6. (Right) Linear regression on absorbance at λ_{max}=326 nm.

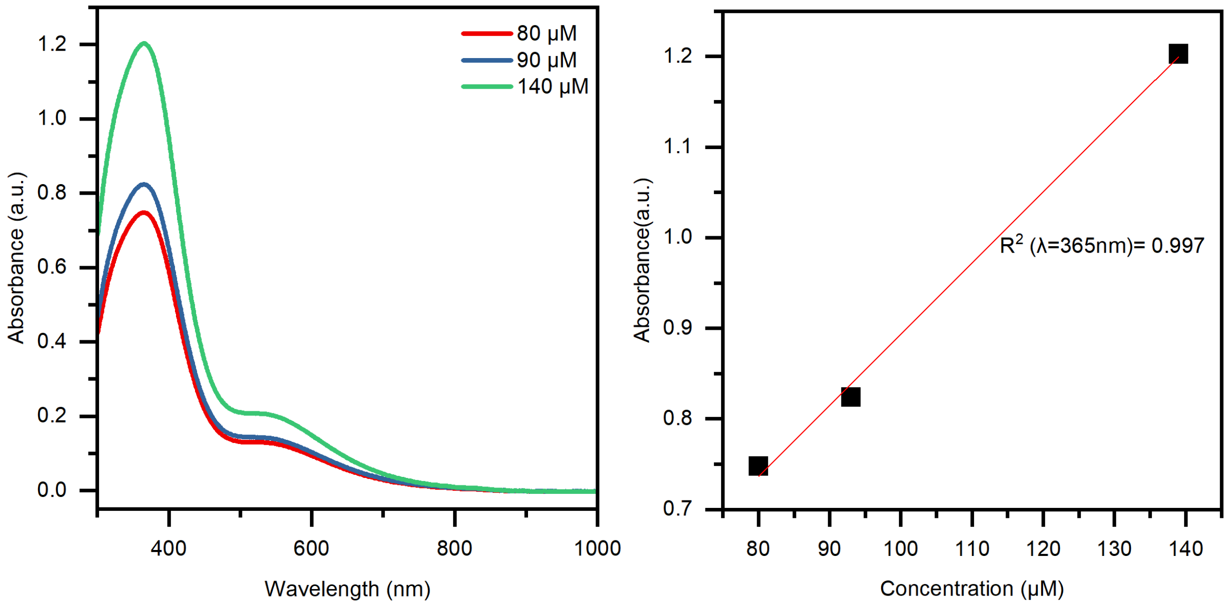

Figure S29. (Left)UV-vis-NIR spectra of **2-CeNpt** in Et_2O. (Right) Linear regression on absorbance at λ_{max}=365 nm. Secondary feature is observed at 545 nm.

Crystallographic Analyses

Crystals suitable for X-ray diffraction were covered with Cargille-NVH oil in a N_2 glovebox and transferred to the diffractometer in a capped 20mL vial. Crystals were mounted on a loop with Cargille-NVH oil on a Bruker D8 VENTURE diffractometer. Diffractometer was cooled to 100K prior to mounting crystals, and kept at 100(2)K throughout the duration of the collection.

Solution of **1-CeI**:

Scaling and absorption correction were done in Apex4 using TWINABS. The data were treated as a two-component twin, with two domains related by a 120° rotation about the *c* axis. The reflections largely overlap, and initially a suitable solution was found using a single domain, however, a much better solution is found after accounting for the twin components. Initial integration of the data yielded 14002 total unique reflections, of which, only 1960 and 1880 reflections could be uniquely assigned to domain 1 and domain 2, respectively. The structure was solved and refined in Olex 2, yielding a satisfactory solution. Atom C20's thermal parameters were restrained using an ISOR command, and a C level alert in the checkcif report notes the prolate shape, which could be due to unresolved disorder or twinning. The C-C bond precision (0.0166 Å) is slightly low, and unresolved disorder within the ligand (either rotational disorder within the ^{t}Bu groups, and/or positional disorder of the entire =P(^{t}Bu)$_3$ fragment) could be contributing to the low C-C bond precision. However, there is no significant residual density within the ligand that can be satisfactorily modeled. Ultimately a satisfactory model was achieved with acceptable R indices.

Table S1. Single-crystal X- ray Crystallography Data

	**1-CeI**	**2-CeBn**	**2-CeNpt**
Empirical Formula	$C_{36}H_{81}CeIN_3P_3$	$C_{43}H_{88}CeN_3P_3$	$C_{41}H_{92}CeN_3P_3$
Formula Weight	915.96	880.19	860.20
Temperature/K	100.0	100.00	100.00
Crystal system	triclinic	monoclinic	monoclinic
Space group	P-1	$P2_1/n$	$P2_1/c$
a/Å	13.220(6)	13.345(3)	18.889(5)
b/Å	13.306(7)	18.516(4)	13.8924(18)
c/Å	15.510(5)	19.445(7)	18.466(5)
α/°	99.15(2)	90	90
β/°	97.760(15)	91.908(12)	90.335(12)
γ/°	119.742(10)	90	90
Volume/Å^3	2264.9(17)	4802(2)	4845.8(18)
Z	2	4	4
ρ_{calc}g/cm^3	1.343	1.217	1.179
μ/mm^{-1}	1.818	1.078	1.066
F(000)	948.0	1880.0	1848.0
Crystal size/mm^3	0.348 × 0.212 × 0.126	0.223 × 0.171 × 0.137	0.211 x 0.143 x 0.118
Radiation	MoKα (λ = 0.71073)	MoKα (λ = 0.71073)	MoKα (λ = 0.71073)
2Θ range for data collection/°	3.994 to 52.744	4.258 to 51.362	4.312 to 51.36
Index ranges	-16 ≤ h ≤ 16, -16 ≤ k ≤ 16, 0 ≤ l ≤ 19	-16 ≤ h ≤ 16, -22 ≤ k ≤ 22, -23 ≤ l ≤ 23	-23 ≤ h ≤ 23, -16 ≤ h ≤ 16, -22 ≤ h ≤ 22
Reflections collected	91257	69911	113028
Independent reflections	9272 [R_{int} = 0.1062, R_{sigma} = 0.0616]	9108 [R_{int} = 0.0527, R_{sigma} = 0.0250]	9184 [R_{int} = 0.0583, R_{sigma} = 0.0233]
Data/restraints/parameters	9272/6/424	9108/243/605	9184/0/471
Goodness-of-fit on F^2	1.098	1.062	1.065
Final R indexes [I>=2σ (I)]	R_1 = 0.0737, wR_2 = 0.1568	R_1 = 0.0224, wR_2 = 0.0522	R_1 = 0.0230, wR_2 = 0.0568
Final R indexes [all data]	R_1 = 0.1091, wR_2 = 0.1750	R_1 = 0.0271, wR_2 = 0.0549	R_1 = 0.0291, wR_2 = 0.0615
Largest diff. peak/hole /e Å^{-3}	1.38/-2.49	1.32/-0.34	0.50/-0.38
CCDC Number	2323502	2323503	2323504

Table S2. Relevant averaged bond lengths and angles for **1-CeI**, **2-CeBz** and **2-CeNpt** compared to values for previously reported Ce^{4+} tetrahomoleptic imidophosphorance complexes.

	Average Distance (Å)		Average angle (°)
Complex	Ce-N	N-P	Ce-N-P
**1-CeI**	2.11(2)	1.58(2)	160(1)
**2-CeBn**	2.142(7)	1.57(1)	167(5)
**2-CeNpt**	2.147(6)	1.566(4)	162.1(7)
Previously reported Ce^{4+} imidophosphorane complexes			
**$Ce^{4+}(NPC)_4$** [10]	2.160(6)	1.539(1)	157.3(7)
**$Ce^{4+}[NP(pip)_3]_4$** [11]	2.20(2)	1.42(4)	166.9(1)

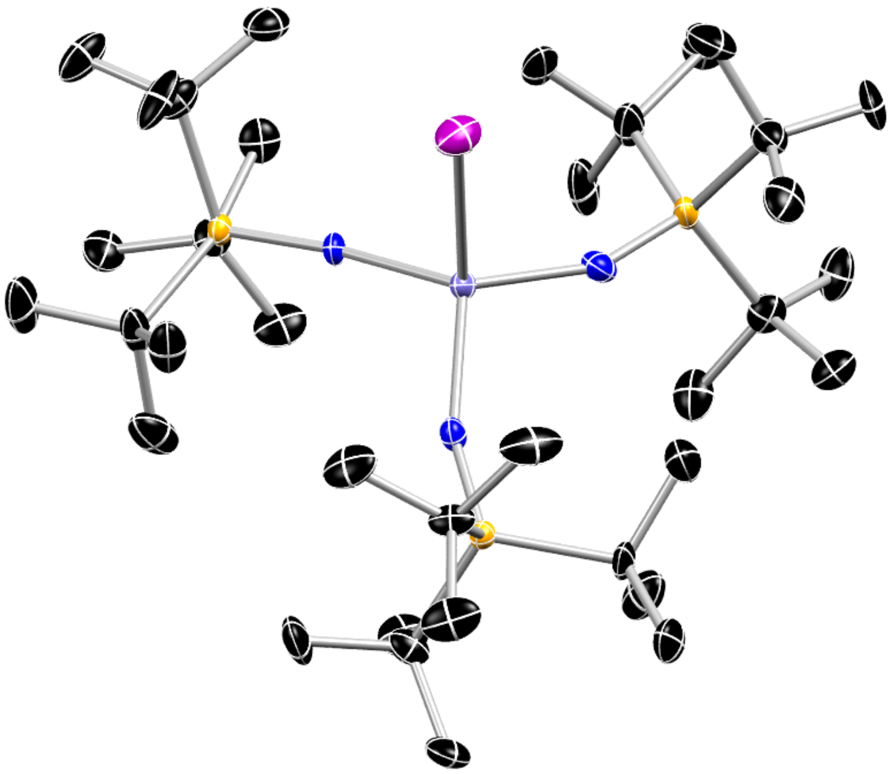

Figure S30. Molecular structure of **1-CeI** with thermal ellipsoids shown at 50% probability. H atoms are omitted for clarity. Ce shown in purple, C shown in black, I shown in magenta, N shown in blue and P shown in orange.

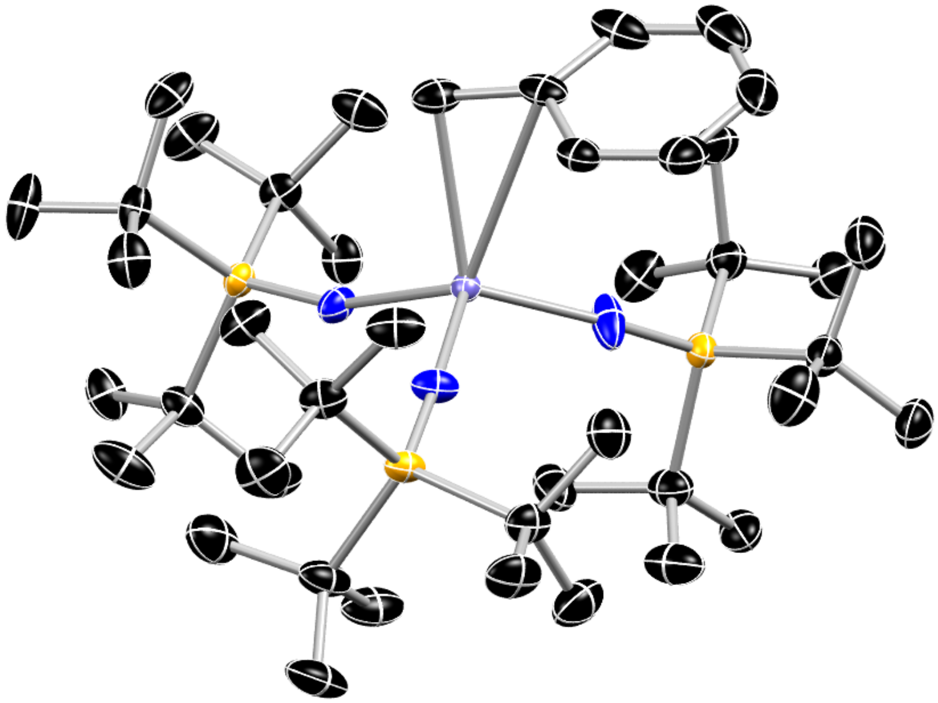

Figure S31. Molecular structure of **2-CeBn** with thermal ellipsoids shown at 50% probability. H atoms are omitted for clarity. Ce shown in purple, C shown in black, N shown in blue and P shown in orange.

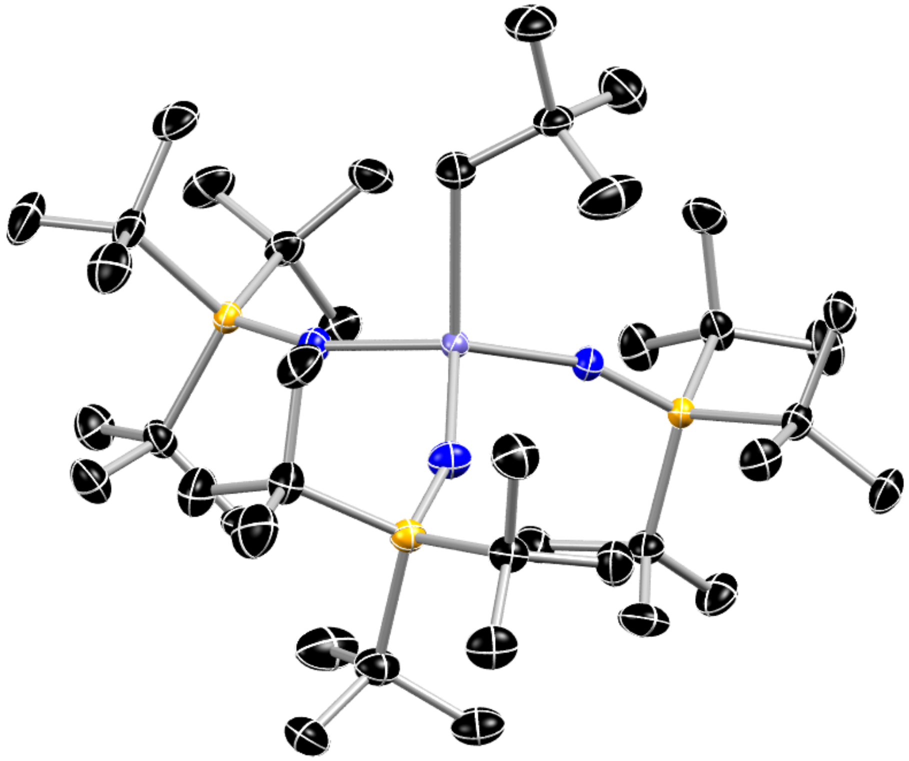

Figure S32. Molecular structure of **2-CeNpt** with thermal ellipsoids shown at 50% probability. H atoms are omitted for clarity. Ce shown in purple, C shown in black, N shown in blue and P shown in orange.

Table S3. Bond length for **1-CeI**.

Atom	Atom	Length/Å	Atom	Atom	Length/Å
Ce1	I1	3.0910(13)	C13	C16	1.537(15)
Ce1	N1	2.107(7)	C13	C15	1.531(14)
Ce1	N3	2.100(7)	C13	C14	1.534(14)
Ce1	N2	2.135(8)	C9	C11	1.540(13)
P1	N1	1.588(7)	C9	C12	1.540(14)
P1	C5	1.899(9)	C9	C10	1.514(13)
P1	C9	1.890(9)	C29	C31	1.536(16)
P1	C1	1.888(9)	C29	C30	1.552(14)
P3	N3	1.583(7)	C29	C32	1.523(14)
P3	C33	1.880(9)	C18	C17	1.531(15)
P3	C29	1.882(10)	C1	C4	1.518(14)
P3	C25	1.896(10)	C1	C3	1.536(13)
P2	N2	1.567(8)	C1	C2	1.558(14)
P2	C13	1.899(10)	C28	C25	1.536(14)
P2	C21	1.895(10)	C21	C24	1.532(13)
P2	C17	1.895(10)	C21	C23	1.540(14)
C36	C33	1.556(13)	C21	C22	1.548(13)
C33	C35	1.535(13)	C27	C25	1.539(12)
C33	C34	1.544(13)	C25	C26	1.551(13)
C5	C6	1.530(13)	C17	C19	1.545(16)
C5	C8	1.518(13)	C17	C20	1.556(14)
C5	C7	1.557(14)			

Table S4. Bond Angles **for 1-CeI.**

Atom	Atom	Atom	Angle/˚	Atom	Atom	Atom	Angle/˚
N1	Ce1	I1	107.80(19)	C15	C13	C16	109.9(9)
N1	Ce1	N2	110.7(3)	C15	C13	C14	109.0(8)
N3	Ce1	I1	105.2(2)	C14	C13	P2	109.8(7)
N3	Ce1	N1	111.3(3)	C14	C13	C16	104.4(9)

N3	Ce1	N2	110.5(3)
N2	Ce1	I1	111.2(2)
N1	P1	C5	109.3(4)
N1	P1	C9	109.9(4)
N1	P1	C1	110.6(4)
C9	P1	C5	108.8(4)
C1	P1	C5	109.2(4)
C1	P1	C9	109.0(4)
N3	P3	C33	108.7(4)
N3	P3	C29	109.8(4)
N3	P3	C25	110.0(4)
C33	P3	C29	109.3(5)
C33	P3	C25	109.3(4)
C29	P3	C25	109.7(5)
N2	P2	C13	109.5(4)
N2	P2	C21	109.1(4)
N2	P2	C17	109.6(4)
C21	P2	C13	109.6(4)
C17	P2	C13	109.5(5)
C17	P2	C21	109.5(5)
P1	N1	Ce1	160.1(4)
P3	N3	Ce1	161.7(5)
P2	N2	Ce1	159.7(5)
C36	C33	P3	109.5(6)
C35	C33	P3	108.7(6)
C35	C33	C36	104.5(8)
C35	C33	C34	109.5(8)
C34	C33	P3	116.1(7)
C34	C33	C36	107.9(8)
C6	C5	P1	109.6(7)
C6	C5	C7	104.2(9)
C8	C5	P1	116.2(7)
C11	C9	P1	108.0(6)
C12	C9	P1	109.5(7)
C12	C9	C11	104.0(8)
C10	C9	P1	117.1(6)
C10	C9	C11	109.8(8)
C10	C9	C12	107.7(8)
C31	C29	P3	110.4(7)
C31	C29	C30	104.3(8)
C30	C29	P3	109.0(7)
C32	C29	P3	114.7(7)
C32	C29	C31	109.3(9)
C32	C29	C30	108.6(9)
C4	C1	P1	109.1(6)
C4	C1	C3	109.8(8)
C4	C1	C2	104.7(8)
C3	C1	P1	115.8(6)
C3	C1	C2	108.7(8)
C2	C1	P1	108.2(7)
C24	C21	P2	110.8(7)
C24	C21	C23	104.9(8)
C24	C21	C22	107.6(8)
C23	C21	P2	107.7(7)
C23	C21	C22	109.8(8)
C22	C21	P2	115.6(7)
C28	C25	P3	108.3(7)
C28	C25	C27	109.2(8)
C28	C25	C26	105.0(9)
C27	C25	P3	114.9(7)
C27	C25	C26	109.3(8)
C26	C25	P3	109.5(7)
C18	C17	P2	109.8(8)
C18	C17	C19	105.8(9)

C8	C5	C6	110.0(8)	C18	C17	C20	109.0(9)
C8	C5	C7	108.0(8)	C19	C17	P2	110.3(7)
C7	C5	P1	108.2(6)	C19	C17	C20	107.5(10)
C16	C13	P2	108.1(6)	C20	C17	P2	114.2(7)
C15	C13	P2	115.1(7)				

Table S5. Bond Lengths for **2-CeBn**.

Atom	Atom	Length/Å	Atom	Atom	Length/Å
Ce1	N1	2.1350(17)	C19	C22	1.535(3)
Ce1	C1	2.562(2)	C23	C24	1.539(4)
Ce1	N2	2.1489(18)	C23	C25	1.541(3)
Ce1	N3	2.1413(19)	C23	C26	1.546(4)
P1	N1	1.5701(17)	C27	C28	1.537(3)
P1	C7	1.892(2)	C27	C29	1.540(3)
P1	C11	1.904(2)	C27	C30	1.543(3)
P1	C15	1.900(2)	P3A	C31A	1.892(6)
C1	C2	1.438(3)	P3A	C35A	1.899(7)
P2	N2	1.5641(19)	P3A	C44	1.891(11)
P2	C19	1.901(2)	C31A	C32A	1.544(6)
P2	C23	1.896(2)	C31A	C33A	1.533(7)
P2	C27	1.902(2)	C31A	C34A	1.553(7)
C2	C3	1.409(3)	C35A	C36A	1.554(7)
C2	C43	1.412(3)	C35A	C37A	1.512(8)
N3	P3A	1.574(6)	C35A	C38A	1.534(6)
N3	P3	1.587(5)	C39A	C44	1.536(9)
C3	C4	1.364(4)	C40A	C44	1.560(9)
C4	C5	1.385(5)	C41A	C44	1.547(10)
C5	C6	1.383(4)	P3	C31	1.901(6)
C6	C43	1.385(3)	P3	C35	1.887(8)
C7	C8	1.543(3)	P3	C39	1.907(9)
C7	C9	1.541(3)	C31	C32	1.545(6)
C7	C10	1.535(3)	C31	C33	1.529(7)

C11	C12	1.529(3)	C31	C34	1.549(6)
C11	C13	1.541(3)	C35	C36	1.550(7)
C11	C14	1.538(3)	C35	C37	1.551(9)
C15	C16	1.535(3)	C35	C38	1.520(8)
C15	C17	1.539(3)	C39	C40	1.545(8)
C15	C18	1.541(3)	C39	C41	1.542(9)
C19	C20	1.537(3)	C39	C42	1.528(8)
C19	C21	1.537(3)			

Table S6. Bond Angles for **2-CeBn**.

Atom	Atom	Atom	Angle/˚	Atom	Atom	Atom	Angle/˚
N1	Ce1	C1	99.74(7)	C25	C23	C26	109.7(2)
N1	Ce1	N2	106.97(7)	C26	C23	P2	107.73(18)
N1	Ce1	N3	108.72(7)	C28	C27	P2	116.29(18)
N2	Ce1	C1	107.42(7)	C28	C27	C29	108.9(2)
N3	Ce1	C1	117.45(8)	C28	C27	C30	109.18(19)
N3	Ce1	N2	115.03(8)	C29	C27	P2	108.75(15)
N1	P1	C7	109.88(10)	C29	C27	C30	105.2(2)
N1	P1	C11	109.74(10)	C30	C27	P2	107.95(16)
N1	P1	C15	110.19(10)	C6	C43	C2	121.5(2)
C7	P1	C11	108.42(10)	N3	P3A	C31A	118.2(3)
C7	P1	C15	108.92(10)	N3	P3A	C35A	105.5(3)
C15	P1	C11	109.67(11)	N3	P3A	C44	106.4(4)
P1	N1	Ce1	162.01(11)	C31A	P3A	C35A	108.7(4)
C2	C1	Ce1	90.43(13)	C44	P3A	C31A	108.6(4)
N2	P2	C19	110.03(10)	C44	P3A	C35A	109.1(4)
N2	P2	C23	110.33(10)	C32A	C31A	P3A	116.1(4)
N2	P2	C27	110.32(11)	C32A	C31A	C34A	110.0(4)
C19	P2	C27	108.58(10)	C33A	C31A	P3A	109.9(4)
C23	P2	C19	108.67(12)	C33A	C31A	C32A	107.5(4)
C23	P2	C27	108.87(11)	C33A	C31A	C34A	104.5(4)
P2	N2	Ce1	173.12(11)	C34A	C31A	P3A	108.2(4)

C3	C2	C1	123.0(2)	C36A	C35A	P3A	107.5(4)
C3	C2	C43	115.5(2)	C37A	C35A	P3A	108.8(4)
C43	C2	C1	121.1(2)	C37A	C35A	C36A	105.6(5)
P3A	N3	Ce1	170.9(3)	C37A	C35A	C38A	108.1(5)
P3	N3	Ce1	164.8(2)	C38A	C35A	P3A	117.4(4)
C4	C3	C2	122.5(3)	C38A	C35A	C36A	108.8(4)
C3	C4	C5	121.0(3)	C39A	C44	P3A	108.8(5)
C6	C5	C4	118.4(3)	C39A	C44	C40A	105.4(6)
C5	C6	C43	121.0(3)	C39A	C44	C41A	108.5(6)
C8	C7	P1	108.51(15)	C40A	C44	P3A	108.4(5)
C9	C7	P1	108.30(15)	C41A	C44	P3A	114.9(6)
C9	C7	C8	105.46(19)	C41A	C44	C40A	110.3(6)
C10	C7	P1	115.89(16)	N3	P3	C31	104.2(3)
C10	C7	C8	107.94(18)	N3	P3	C35	115.8(3)
C10	C7	C9	110.2(2)	N3	P3	C39	110.3(4)
C12	C11	P1	109.68(16)	C31	P3	C39	108.6(3)
C12	C11	C13	108.4(2)	C35	P3	C31	108.3(4)
C12	C11	C14	105.68(19)	C35	P3	C39	109.2(4)
C13	C11	P1	115.47(16)	C32	C31	P3	116.4(3)
C14	C11	P1	107.70(15)	C32	C31	C34	107.7(4)
C14	C11	C13	109.5(2)	C33	C31	P3	108.6(3)
C16	C15	P1	108.85(15)	C33	C31	C32	109.6(4)
C16	C15	C17	106.1(2)	C33	C31	C34	105.5(4)
C16	C15	C18	108.00(19)	C34	C31	P3	108.6(3)
C17	C15	P1	108.60(16)	C36	C35	P3	108.4(4)
C17	C15	C18	108.86(19)	C36	C35	C37	103.5(5)
C18	C15	P1	116.03(16)	C37	C35	P3	108.9(4)
C20	C19	P2	109.07(16)	C38	C35	P3	118.2(5)
C20	C19	C21	105.3(2)	C38	C35	C36	109.5(5)
C21	C19	P2	108.52(16)	C38	C35	C37	107.4(5)
C22	C19	P2	115.86(17)	C40	C39	P3	108.1(4)
C22	C19	C20	108.1(2)	C41	C39	P3	115.9(5)

C22	C19	C21	109.4(2)	C41	C39	C40	108.8(6)
C24	C23	P2	109.35(17)	C42	C39	P3	108.6(5)
C24	C23	C25	108.5(2)	C42	C39	C40	106.7(5)
C24	C23	C26	105.3(2)	C42	C39	C41	108.5(5)
C25	C23	P2	115.78(17)				

Table S7. Bond lengths for **2-CeNpt.**

Atom	Atom	Length/A	Atom	Atom	Length/A
Cel	N1	2.1476(18)	C9	C12	1.536(3)
Cel	N2	2.142(2)	C13	C14	1.538(4)
Cel	N3	2.1523(18)	C13	C15	1.532(4)
Cel	C37	2.508(2)	C13	C16	1.536(4)
P1	N1	1.5617(18)	C17	C18	1.540(4)
P1	C1	1.904(2)	C17	C19	1.537(4)
P1	C5	1.906(2)	C17	C20	1.546(3)
P1	C9	1.899(2)	C21	C22	1.537(3)
P2	N2	1.567(2)	C21	C23	1.533(3)
P2	C13	1.900(2)	C21	C24	1.539(3)
P2	C17	1.901(2)	C25	C26	1.542(3)
P2	C21	1.897(2)	C25	C27	1.537(4)
P3	N3	1.5687(18)	C25	C28	1.534(3)
P3	C25	1.901(2)	C29	C30	1.538(3)
P3	C29	1.899(2)	C29	C31	1.538(3)
P3	C33	1.898(2)	C29	C32	1.540(3)
C1	C2	1.537(3)	C33	C34	1.537(3)
C1	C3	1.551(3)	C33	C35	1.540(3)
C1	C4	1.532(3)	C33	C36	1.537(3)
C5	C6	1.542(3)	C37	C38	1.528(3)
C5	C7	1.538(3)	C38	C39	1.524(4)
C5	C8	1.532(4)	C38	C40	1.518(4)
C9	C10	1.534(3)	C38	C41	1.541(3)
C9	C11	1.543(3)			

Table S8. Bond angles for **2-CeNpt.**

Atom	Atom	Atom	Angle/°	Atom	Atom	Atom	Angle/°
N1	Ce1	N3	111.87(7)	C12	C9	C11	109.23(19)
N1	Ce1	C37	104.02(8)	C14	C13	P2	108.26(17)
N2	Ce1	N1	110.06(7)	C15	C13	P2	108.94(18)
N2	Ce1	N3	111.24(8)	C15	C13	C14	104.5(2)
N2	Ce1	C37	108.47(9)	C15	C13	C16	110.2(2)
N3	Ce1	C37	110.90(8)	C16	C13	P2	115.77(18)
N1	P1	C1	110.12(10)	C16	C13	C14	108.6(2)
N1	P1	C5	109.89(10)	C18	C17	P2	107.82(17)
N1	P1	C9	110.29(10)	C18	C17	C20	105.5(2)
C1	P1	C5	108.94(11)	C19	C17	P2	115.26(17)
C9	P1	C1	108.50(10)	C19	C17	C18	109.8(2)
C9	P1	C5	109.05(10)	C19	C17	C20	108.2(2)
N2	P2	C13	110.61(11)	C20	C17	P2	109.89(16)
N2	P2	C17	110.92(11)	C22	C21	P2	115.66(16)
N2	P2	C21	109.94(10)	C22	C21	C24	108.23(19)
C13	P2	C17	108.20(11)	C23	C21	P2	108.67(16)
C21	P2	C13	108.72(11)	C23	C21	C22	109.0(2)
C21	P2	C17	108.39(10)	C23	C21	C24	105.06(19)
N3	P3	C25	110.05(10)	C24	C21	P2	109.69(16)
N3	P3	C29	110.21(10)	C26	C25	P3	108.10(15)
N3	P3	C33	109.83(11)	C27	C25	P3	108.54(16)
C29	P3	C25	109.04(10)	C27	C25	C26	105.4(2)
C33	P3	C25	109.18(10)	C28	C25	P3	116.33(16)
C33	P3	C29	108.49(10)	C28	C25	C26	109.23(19)
P1	N1	Ce1	162.81(12)	C28	C25	C27	108.7(2)
P2	N2	Ce1	161.50(12)	C30	C29	P3	108.16(14)
P3	N3	Ce1	162.10(12)	C30	C29	C31	108.72(19)
C2	C1	P1	109.85(16)	C30	C29	C32	105.41(18)

C2	C1	C3	104.69(18)	C31	C29	P3	116.34(15)
C3	C1	P1	107.73(16)	C31	C29	C32	108.66(18)
C4	C1	P1	116.16(17)	C32	C29	P3	109.01(15)
C4	C1	C2	108.3(2)	C34	C33	P3	116.14(17)
C4	C1	C3	109.5(2)	C34	C33	C35	109.7(2)
C6	C5	P1	109.26(15)	C35	C33	P3	107.68(16)
C7	C5	P1	115.40(17)	C36	C33	P3	109.62(15)
C7	C5	C6	108.4(2)	C36	C33	C34	107.76(19)
C8	C5	P1	108.55(16)	C36	C33	C35	105.38(19)
C8	C5	C6	105.1(2)	C38	C37	Ce1	122.73(15)
C8	C5	C7	109.7(2)	C37	C38	C41	112.34(19)
C10	C9	P1	109.41(16)	C39	C38	C37	109.7(2)
C10	C9	C11	105.5(2)	C39	C38	C41	108.0(2)
C10	C9	C12	108.30(19)	C40	C38	C37	108.9(2)
C11	C9	P1	108.07(14)	C40	C38	C39	109.7(2)
C12	C9	P1	115.88(17)	C40	C38	C41	108.2(2)

Computational Details

DFT calculations along with Wiberg and Mayer bond analysis were performed using a developmental version of the Gaussian electronic structure software package. The cc-pVTZ-X2C basis set was used for Ce atoms[13–16]. The 6-311G basis set was used for C and P atoms[13–15,17,18]. The 6-311G** basis set was used for N atoms[13,15,17]. The STO-3G basis set was used for H atoms[19]. NMR chemical shift calculations were performed using the Amsterdam Density Functional electronic structure software package. Chemical shifts were computed for **2-CeNpt** and **2-CeBn** using DFT with the relativistic zeroth-order regular approximation (ZORA) of the Dirac equation, including spin-orbit coupling, and the PBE0 functional.[20–27] An all-electron ZORA-optimized triple-zeta Slater-type orbital basis set with additional polarization functions (ZORA/TZ2P) was used.[28] Tetramethylsilane was used as the reference. Table S11 shows the theoretical and experimental ^{1}H and ^{13}C NMR chemical shifts of **2-CeNpt** and **2-CeBn**.

Table S9. Ce contribution to Ce-X σ bond.

	**% Ce**	**% *s***	**% *d***	**% *f***
**σ Ce-Bn**	9.6	5.5	35.9	55.9
**σ Ce-Npt**	9.3	14.2	48.6	37.2

Table S10. The orbital contribution to the bonding orbital from the C directly bonding to Ce.

	**C atom**	**% C contribution**	**% *s***	**% *p***
**CeBn**	C1	45.58	8.36	91.64
**CeNpt**	C37	64.54	12.27	87.73

Table S11. Theoretical and experimental ^{1}H and ^{13}C NMR chemical shifts of **2-CeNpt** and **2-CeBn** in parts per million (ppm).

	**^{1}H (Theoretical)**	**^{1}H (Experiment)**	**^{13}C (Theoretical)**	**^{13}C (Experiment)**
**2-CeBn**	0.46	2.43	79.36	90.12
**2-CeNpt**	-1.51	1.43	101.07	107.85

Analysis on the contribution of % s character to the ^{13}C NMR chemical shift of 2-CeBn and 2-CeNpt:

As outlined in the main text, the degree *s* character in the carbon is one of the critical factors in determining the magnitude of the Spin Orbit-Heavy Atom on Light Atom(SO-HALA) effect. Based on the hybridization of the methylene in the benzyl (*sp*^{2}) and the neopentyl (*sp*^{3}), a first-order analysis would suggest that the benzyl complex would have greater C *s* character in the Ce^{4+}–C bond. However, as is clear in the main text **Fig. 3A**, the methylene carbon of **2-CeBn** has dominant *p* orbital contribution. This contribution to the bond is reflected in the calculated orbital contribution to the bond from the C atom in **2-CeBn** with % *s* of 8.36 and % *p* of 91.64 as shown in Table **S10**. In **2-CeNpt**, the calculated orbital contribution to the C bound to Ce^{4+} has % *s* of 12.27 and % *p* of 87.73.

Therefore, the observed shifts are the result of two antagonistic contributions to the SO-HALA effect. The smaller % *s* character in the Ce-C in the **2-CeBn** decreases the overall SO-HALA effect on the ^{13}C chemical shift. However, as the bond has greater Ce 4*f* contribution, it still experiences a greater downfield shift than **2-CeNpt**.

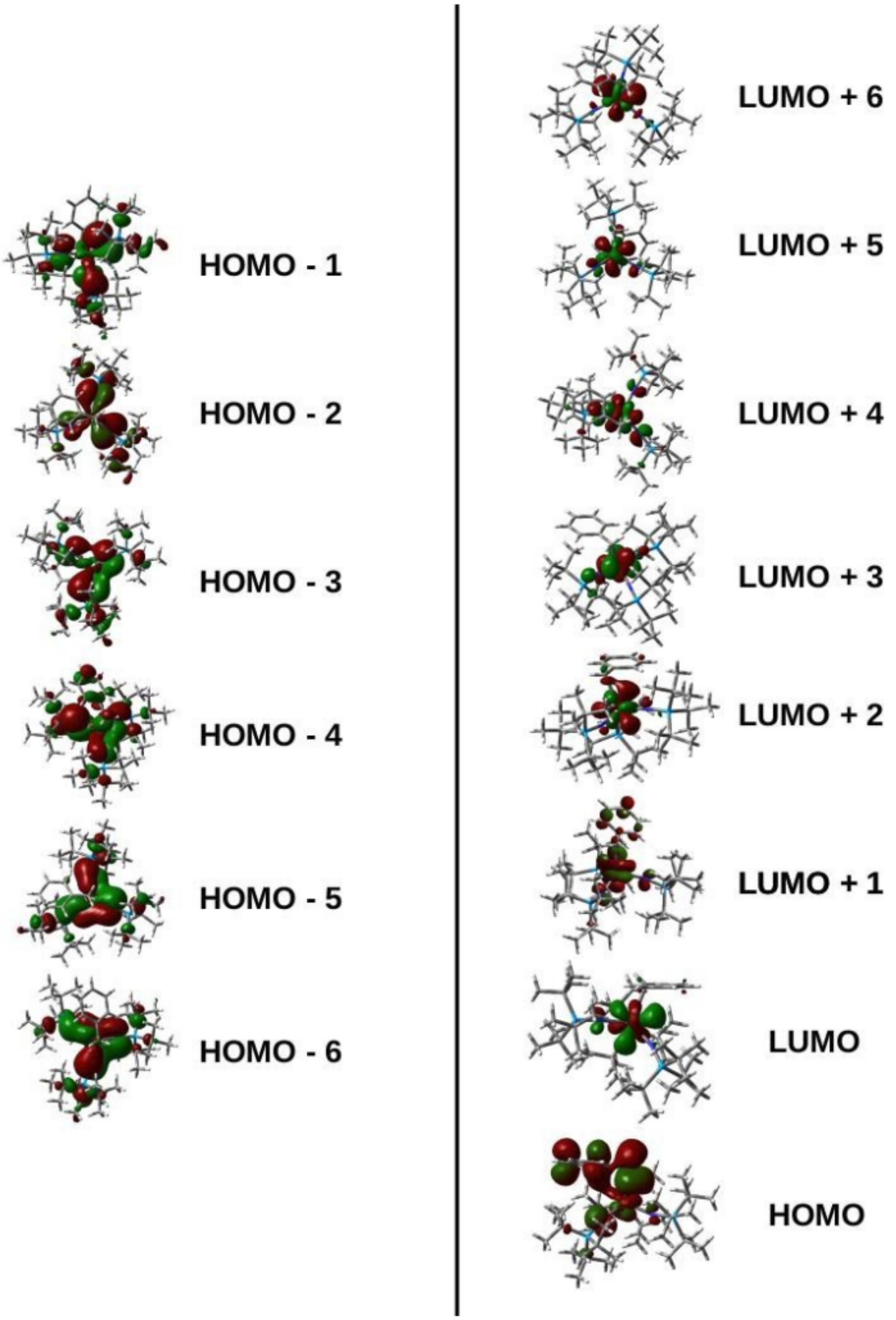

Figure S33. 2-CeBn Kohn-Sham orbitals

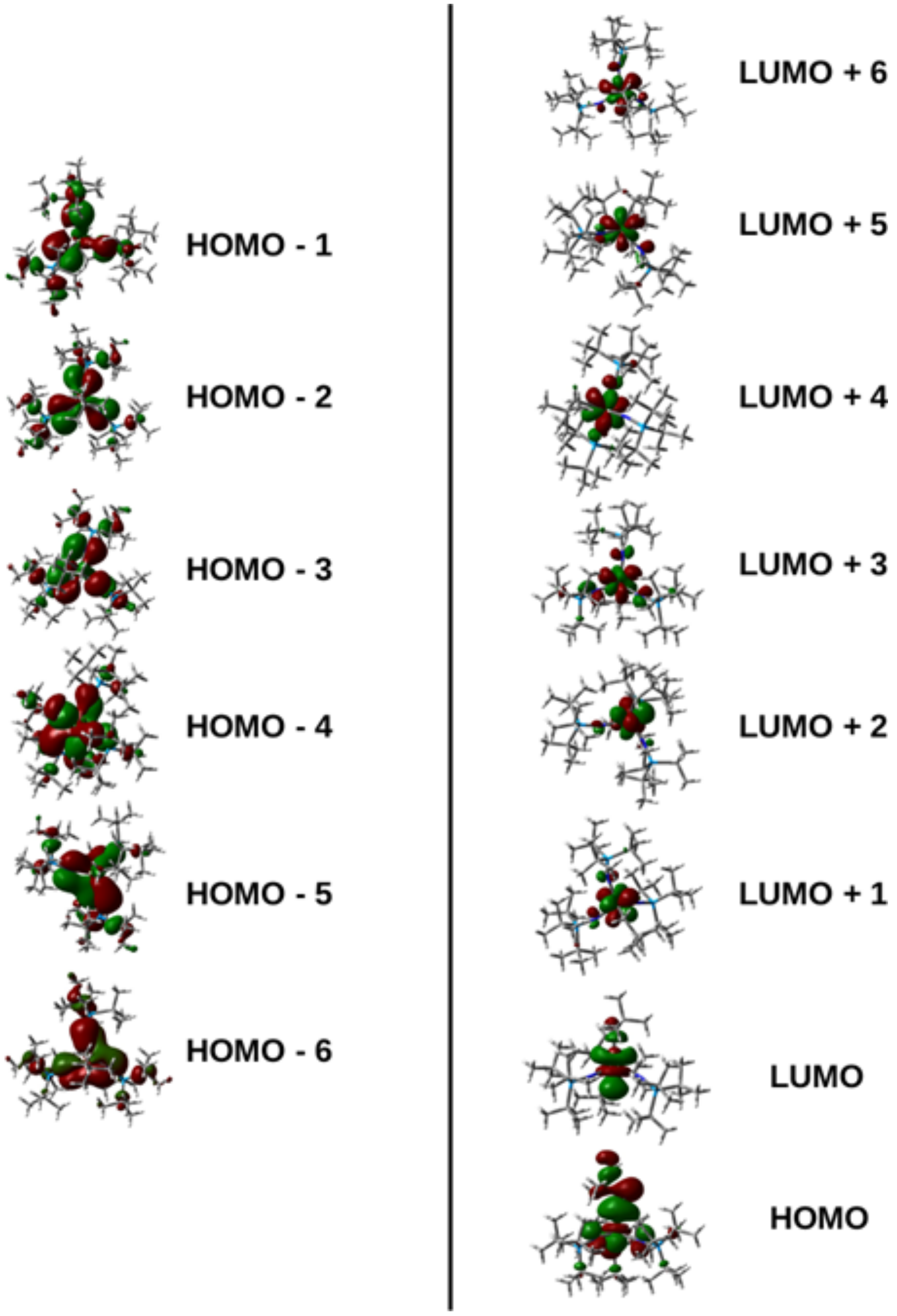

Figure S34. 2-CeNpt Kohn-Sham orbitals

Bond order analysis[29] is shown in **Table S12**.

**CeBn**	**WBO**	**MBO**	**DI-QTAIM**
**Ce-C1**	0.39	0.45	0.48
**Ce-C2**	0.05	0.00	0.08
**Ce-C3**	0.06	0.01	0.03
**Ce-C43**	0.10	0.05	0.08
**Ce-N***	0.74	1.19	0.96
**CeNpt**			
**Ce-C37**	0.46	0.53	0.60
**Ce-C38**	0.03	-0.07	0.02
**Ce-N***	0.69	1.17	0.95

*Averaged over the three Ce-N bonds

Table S12. Wiberg bond order (WBO), Mayer bond order (MBO), and delocalization index obtained from QTAIM analysis (DI-QTAIM).

2-CeBn

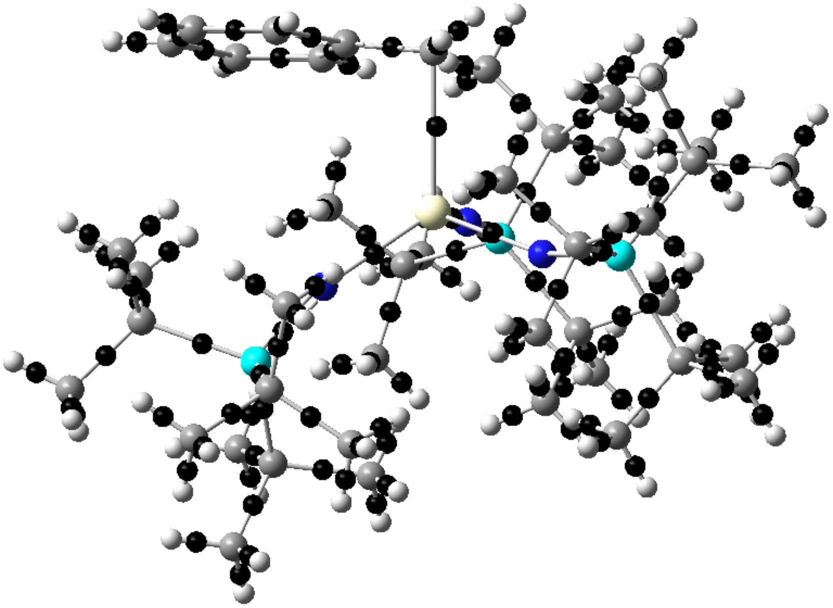

2-CeNpt

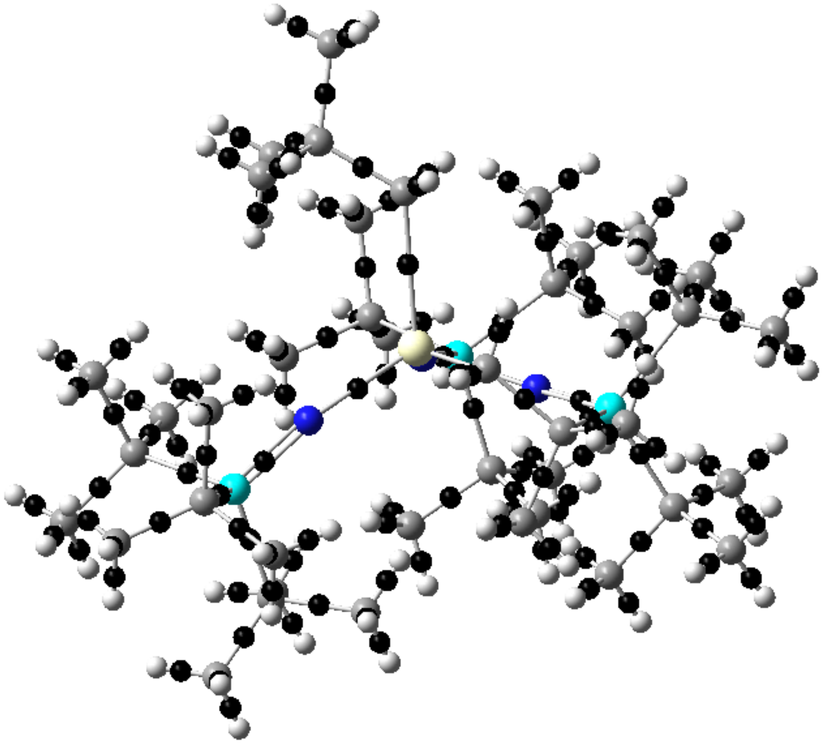

Figure S35. Bonding critical points (BCPs) of **2-CeBn** and **2-CeNpt** shown as black points.

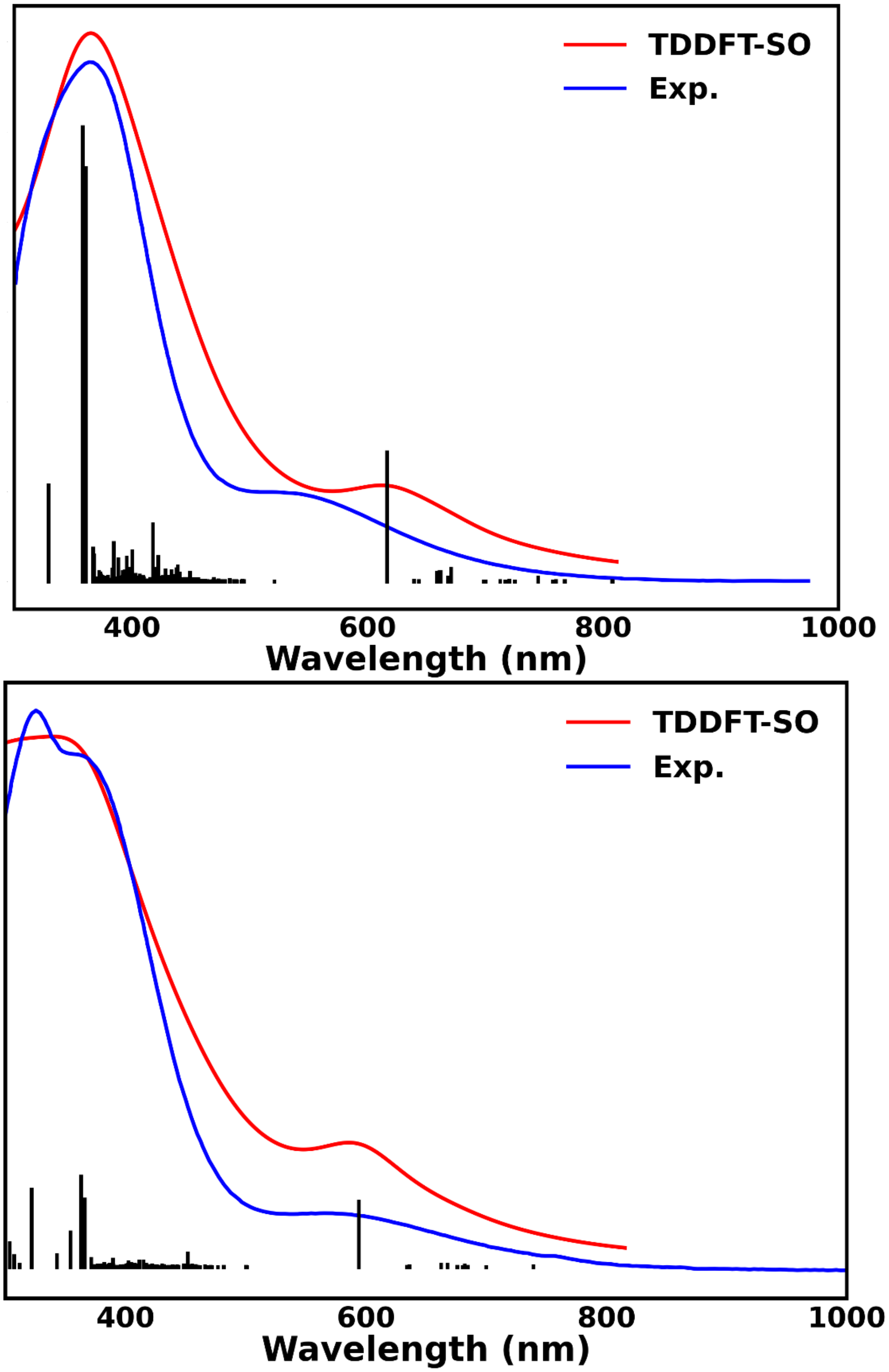

Figure S36. UV-Vis spectra of **2-CeBn** (top) and **2-CeNpt** (bottom), calculated with solvent [benzene (**2-CeBn**) and Et_2O(**2-CeNpt**)] included using a polarized continuum model. Lorentzian broadening was applied to the theoretical spectra with a broadening parameter of 100 nm. The theoretical **2-CeBn** and **2-CeNpt** spectra are red-shifted by 20 nm and 10 nm, respectively. Excited states responsible for the spectra are shown as black lines scaled by their respective oscillator strengths.

Table S13. BCP coordinates for **2-CeBn.**

BCP Number	x	y	z
1	3.259949	-5.38709	1.109841
2	1.253444	-5.06242	-0.29524
3	-3.90484	-4.61889	-1.40841
4	1.130293	-4.25666	1.908271
5	-2.12892	-4.28784	0.339521
6	3.858699	-4.9048	0.528038
7	3.769353	-4.64154	1.450141
8	-4.47528	-3.98744	-0.95477
9	1.871137	-4.73589	-0.95617
10	2.918486	-4.38048	0.790036
11	-2.71904	-3.68247	0.79783
12	1.928362	-4.245	0.033763
13	1.669351	-3.53478	2.251872
14	1.851747	-3.8156	1.201298
15	1.102918	-4.20236	-0.71477
16	-4.25462	-3.85665	-1.88239
17	-1.8201	-3.39486	0.561291
18	-1.53288	-4.10506	-1.91895
19	-3.43708	-3.63679	-1.15865
20	-2.55018	-3.47816	-0.26529
21	0.944386	-3.34646	1.63405
22	-4.83214	-2.36473	1.15948
23	-2.24856	-3.38102	-1.46782
24	4.185539	-4.01574	-1.97346
25	-1.88405	-3.43701	-2.51433
26	-1.2175	-3.19068	-1.85904
27	2.640604	-2.98018	0.273932
28	-5.22989	-1.6167	1.613027
29	-4.26326	-1.72156	1.609378
30	3.847836	-2.35366	2.723017
31	4.931091	-3.48726	-1.66918
32	-1.71029	-1.07071	4.245856
33	5.599022	-2.73137	0.972389
34	-2.9565	-2.21158	-0.91091
35	-5.96687	-1.81688	-0.99449
36	4.677379	-3.47134	-2.59944
37	-3.52635	-0.53105	4.482337
38	-4.73051	-1.39595	0.65916
39	2.086392	-2.9457	-2.69613

40	4.003748	-2.92188	-1.9042
41	3.28265	-1.56817	2.702102
42	4.216297	-1.4976	2.959896
43	-2.50616	-0.36248	4.168192
44	-6.44256	-1.15074	-0.48403
45	4.027673	-1.66557	1.88816
46	3.178621	-2.26609	-0.80805
47	-5.33075	-1.11651	-0.40949
48	3.50808	-1.9012	0.528168
49	6.000645	-1.93113	1.330632
50	0.579613	-0.66867	3.465944
51	4.930214	-1.84562	1.034674
52	2.367384	-1.79881	0.015969
53	-3.82298	-1.17732	-0.46787
54	5.81908	-2.00055	0.386164
55	2.919476	-2.37334	-2.25878
56	-3.63612	-1.96326	-3.74078
57	-1.37488	-0.13067	3.814684
58	-2.55554	-1.14416	-0.50014
59	-5.97507	-0.90056	-1.28878
60	2.528107	-2.32872	-3.28767
61	-4.43863	-1.5004	-3.47487
62	1.932385	-1.9901	-2.59879
63	-3.37698	0.458853	4.057515
64	-3.0744	-1.04449	-1.66081
65	-0.17204	0.075615	3.406079
66	-4.69485	-0.2193	0.221495
67	3.902398	-1.67358	-1.94483
68	0.900907	-0.67728	0.395892
69	-0.9854	-0.45831	0.333885
70	0.296847	-0.08902	2.090783
71	-3.49135	-1.05629	-3.13683
72	0.872104	0.26215	3.450409
73	4.264619	-0.76611	1.062316
74	-3.87628	-1.13097	-4.16648
75	-4.12317	1.239595	3.932644
76	-1.38678	-0.99325	-3.1956
77	-5.13974	0.600194	0.810863
78	-1.11525	0.934996	3.338857
79	-2.35287	-0.56898	-2.86109
80	-4.17306	0.641141	0.700492

81	4.756457	-0.99015	-1.7993
82	-3.1331	1.511184	3.580141
83	4.412073	-1.04227	-2.69608
84	-4.76839	0.846054	-0.04131
85	-3.38192	0.06133	-2.58974
86	3.751514	0.219189	1.059227
87	4.664693	0.206157	1.389286
88	-1.32758	-0.16371	-2.69396
89	3.915736	-0.5647	-2.01503
90	4.497634	0.114439	0.446818
91	-1.64582	-0.16211	-3.60942
92	-2.00475	1.751605	3.212956
93	-4.23739	0.7589	-2.46575
94	0.22851	1.077012	0.394555
95	-0.98584	1.927147	2.892664
96	-2.83387	2.454133	3.126379
97	-3.38697	1.087295	-2.14611
98	-3.56423	1.036302	-3.09639
99	1.085814	1.506778	-2.52518
100	2.556336	2.932404	1.615317
101	1.429975	1.875959	-3.35414
102	0.623971	2.833531	-0.24979
103	3.154242	2.249663	-1.39601
104	0.558649	2.138447	-3.04241
105	-1.85038	3.560933	0.385698
106	3.232537	3.207035	0.974268
107	1.415005	2.571264	-2.49645
108	3.143634	3.681058	1.807136
109	3.438719	2.532809	-2.27819
110	-1.53538	3.236033	-2.05016
111	2.465736	2.936572	-1.93724
112	1.218647	3.30607	-1.23526
113	2.360254	3.872033	1.057719
114	0.64382	4.420357	2.097315
115	3.473929	3.150548	-1.54185
116	1.274259	3.853732	0.060257
117	-1.59756	4.412085	0.783668
118	0.060291	3.871864	-0.66335
119	-2.31615	4.37929	0.144701
120	-1.29957	4.273993	-0.26768
121	-1.13287	4.109677	-1.48888

122	1.402586	4.621469	1.310605
123	-2.0055	4.073593	-2.1727
124	-1.14463	3.941309	-2.5815
125	1.315371	5.084905	2.312601
126	1.782277	3.789648	-2.56053
127	0.613932	5.294696	1.687718
128	2.230006	4.967589	0.436664
129	-0.69591	5.159322	-0.94726
130	2.161203	4.277696	-3.48578
131	1.284101	4.553166	-3.19462
132	2.983585	5.379753	-0.26324
133	2.065083	4.852556	-2.71734
134	2.907935	5.83492	0.583423
135	2.22387	5.944188	-0.08759
136	-0.41238	6.149686	-0.53556
137	-1.12308	6.156366	-1.18825
138	-0.21178	6.010392	-1.46706

Table S14. BCP coordinates for **2-CeNpt.**

BCP Number	x	y	z
1	-2.45767	-4.66868	0.031432
2	-4.90062	-4.39539	-0.44451
3	-3.89142	-3.86338	1.713852
4	3.440524	-5.21729	-0.64948
5	1.233594	-4.54524	0.315402
6	3.17907	-4.30121	1.625425
7	-2.24599	-4.1816	-0.76998
8	-4.66267	-3.93053	-1.25496
9	-1.8746	-3.89336	0.080543
10	-2.95355	-3.69485	-0.08595
11	-4.33053	-3.00837	1.673054
12	-5.25377	-3.51538	-0.619
13	-4.18375	-3.55375	-0.32799
14	-3.37829	-3.0495	1.837588
15	-3.71038	-3.26791	0.798099
16	3.254182	-4.6888	-1.43424
17	4.066453	-4.54273	-0.93858
18	-0.18704	-2.63962	4.111638
19	1.058478	-4.03977	-0.48476
20	3.137751	-4.16306	-0.46373
21	3.884813	-3.68045	1.420135

22	2.016301	-3.8323	0.015108
23	0.993488	-3.60745	0.38269
24	3.045319	-3.70145	0.704372
25	3.041683	-3.35031	1.760703
26	-0.94878	-2.17849	3.762223
27	-0.19043	-2.34651	3.201919
28	-0.01622	-1.05495	5.965136
29	-0.05238	-1.54501	3.965361
30	-3.35721	-2.28483	-0.25237
31	-2.87793	-3.03997	-2.91967
32	1.930766	-1.42832	4.488511
33	2.854933	-2.67613	-0.33756
34	1.474512	-3.13624	-2.74411
35	-0.77415	-0.56475	5.644794
36	-5.93934	-1.23861	0.573753
37	0.031662	-0.75363	4.885356
38	-2.02243	-2.60573	-2.8056
39	0.993204	-0.94216	4.159951
40	5.746977	-2.65666	-0.12635
41	1.938031	-1.09326	3.589327
42	-2.5408	-2.46071	-3.6103
43	0.057375	-0.11294	5.80789
44	0.914299	-2.41677	-2.42204
45	-4.07065	-0.55367	2.056302
46	-5.00961	-1.74122	-2.75149
47	-2.92224	-1.95457	-2.69669
48	-2.5636	-1.32879	-0.28394
49	-6.0251	-0.88097	-0.31614
50	3.90683	-2.77618	-3.20481
51	1.990348	-0.48726	4.335065
52	-3.22016	-1.41712	-1.34669
53	1.192116	-2.44524	-3.35053
54	5.778034	-2.35409	-1.03981
55	-3.743	-0.93654	-0.13106
56	0.015658	-0.32542	3.679383
57	1.977935	-2.16207	-2.62572
58	-5.14816	-0.52635	0.261429
59	-6.22489	-0.32594	0.446581
60	4.59874	-1.26608	1.628763
61	2.47597	-1.48566	-0.28163
62	2.783965	-1.80419	-1.44949

63	6.313288	-1.91439	-0.36961
64	-3.99589	-1.30382	-2.64256
65	3.711263	-1.56179	-0.41322
66	5.223034	-1.70122	-0.33559
67	-4.21607	-0.17155	1.033716
68	-4.6856	-1.24344	-3.51131
69	3.190547	-1.9866	-2.9023
70	-0.94428	-0.58488	0.332897
71	-3.46582	0.153724	1.781855
72	3.590222	-2.20548	-3.91451
73	-4.39151	0.352305	1.986597
74	-0.75448	0.309988	3.301192
75	-4.9706	-0.77956	-2.71513
76	4.659569	-0.99883	0.562319
77	0.983412	-0.53855	0.398115
78	-0.08707	0.086734	2.055401
79	4.228248	-1.871	-3.27354
80	-2.8712	-0.71356	-2.59123
81	5.194872	-0.54327	1.408123
82	4.235427	-0.39441	1.392643
83	0.070372	0.679691	3.391408
84	-4.34704	0.401994	-0.05167
85	2.435179	-1.00587	-2.63716
86	4.782969	-0.56137	-0.59428
87	-1.91781	-0.14302	-2.60004
88	-2.46107	-0.09009	-3.40118
89	-2.73627	0.38071	-2.60776
90	-4.49516	1.060675	-0.91982
91	1.826948	-0.09258	-2.45349
92	2.071223	-0.27074	-3.37528
93	-3.70261	1.239285	-0.39506
94	-4.58016	1.481366	-0.05924
95	4.95783	-0.04611	-1.55573
96	-0.03992	1.102264	0.352267
97	2.737826	0.047414	-2.75886
98	5.43078	0.313939	-0.79969
99	4.479318	0.466362	-0.89208
100	2.435484	2.437955	1.102573
101	-2.17336	3.413063	1.251843
102	2.231241	3.166911	1.711755
103	0.073793	2.925794	-0.12941

104	-0.04492	2.368279	-2.73132
105	3.104553	3.107707	1.308251
106	-0.12776	3.878237	2.54477
107	2.665694	2.809713	-1.30825
108	2.287758	3.44123	0.64063
109	-2.5017	4.273912	1.557558
110	-2.50189	4.070418	0.617363
111	0.749362	2.875438	-2.97032
112	-1.52142	4.288869	1.060012
113	1.04649	3.711663	-0.10764
114	-2.04005	3.598293	-1.9118
115	-0.55352	4.711427	2.803003
116	-0.26521	4.039006	0.336679
117	-0.06145	2.992023	-3.47492
118	-0.49207	4.523114	1.712696
119	3.419543	3.35277	-1.03462
120	-0.01421	3.777405	-1.03338
121	2.445652	3.619449	-0.57836
122	0.330043	4.721909	2.422049
123	-0.04901	3.451249	-2.47154
124	2.758608	3.714645	-1.63244
125	-1.04276	4.067445	-2.05198
126	-1.94395	4.173365	-2.68482
127	-2.00006	4.557611	-1.8046
128	2.238306	4.614663	0.151727
129	-0.85471	5.340525	0.827314
130	0.023766	4.695208	-2.24305
131	2.191455	5.52995	0.772926
132	3.023291	5.382195	0.306851
133	-1.17514	6.369522	1.101459
134	-1.21484	6.152673	0.162698
135	2.241894	5.670807	-0.17817
136	-0.36348	6.305814	0.584594
137	0.901531	5.31208	-2.52324
138	0.099177	5.471273	-3.03402
139	0.165049	5.788842	-2.1261

Table S15. Geometry optimized coordinates for **2-CeBn.**

Atom	x	y	z
Ce	0.011395	-0.01577	0.751128
P	2.860435	-2.18854	0.0012

N	1.629203	-1.21488	0.042042
C	0.462717	-0.125	3.270787
H	0.664233	-1.03749	3.597655
H	1.149032	0.518827	3.578277
P	0.774748	3.391337	-0.49233
N	0.398801	1.999768	0.114647
C	-0.88488	0.298232	3.541584
N	-1.82726	-0.85228	0.040452
C	-1.86174	-0.55578	4.089386
H	-1.60634	-1.4312	4.354862
C	-3.16085	-0.17384	4.253433
H	-3.78101	-0.77969	4.641836
C	-3.59018	1.083785	3.862441
H	-4.49507	1.348622	3.982111
C	-2.66977	1.944942	3.293274
H	-2.94849	2.808105	3.010871
C	2.341544	-3.90757	0.597903
C	1.514739	-4.58363	-0.51576
H	0.836183	-3.95767	-0.84463
H	1.07552	-5.38283	-0.15708
H	2.107534	-4.83974	-1.25297
C	1.370949	-3.73588	1.783187
H	1.863426	-3.40245	2.562512
H	0.964215	-4.60076	1.999935
H	0.669807	-3.09603	1.541441
C	3.485166	-4.85743	0.980751
H	4.108499	-4.93802	0.228905
H	3.118153	-5.74004	1.196844
H	3.958798	-4.50066	1.760994
C	4.231278	-1.54015	1.153244
C	3.834875	-1.77835	2.610225
H	2.929945	-1.43445	2.762945
H	4.464666	-1.31407	3.200305
H	3.855737	-2.73929	2.801598
C	5.630101	-2.14513	0.92463
H	5.580958	-3.12071	1.003614
H	6.251652	-1.79391	1.595872
H	5.94712	-1.90557	0.028769
C	4.299508	-0.01309	0.985292
H	4.629639	0.202514	0.088241
H	4.907344	0.362403	1.656138

H	3.404954	0.368926	1.10483
C	3.527968	-2.32739	-1.77174
C	4.269761	-1.03078	-2.12554
H	3.688718	-0.26235	-1.94459
H	4.510878	-1.04109	-3.07537
H	5.082502	-0.96067	-1.58276
C	2.334561	-2.41346	-2.74042
H	1.919105	-3.29821	-2.66912
H	2.648035	-2.27283	-3.65819
H	1.676901	-1.72426	-2.51058
C	4.47836	-3.50958	-2.04583
H	5.232089	-3.47456	-1.42044
H	4.813545	-3.4529	-2.96493
H	3.993669	-4.35291	-1.92629
C	1.852009	4.367047	0.733547
C	0.969159	4.871713	1.886345
H	0.375179	5.578488	1.557613
H	1.537332	5.228486	2.600727
H	0.432395	4.129941	2.235506
C	2.853498	3.397785	1.38264
H	2.370058	2.635113	1.763385
H	3.343344	3.861805	2.093449
H	3.485423	3.07877	0.705043
C	2.60937	5.567719	0.150671
H	3.232959	5.257128	-0.53849
H	3.108246	6.017011	0.864382
H	1.970012	6.195614	-0.24633
C	1.744517	3.165771	-2.10619
C	3.176184	2.704638	-1.78075
H	3.675713	3.447384	-1.38198
H	3.621547	2.416219	-2.60458
H	3.142482	1.956712	-1.14843
C	1.825901	4.405451	-3.01777
H	0.925511	4.653045	-3.31351
H	2.381949	4.200451	-3.79835
H	2.223428	5.150546	-2.52039
C	1.105068	1.989825	-2.87955
H	1.071513	1.198889	-2.30249
H	1.641477	1.791873	-3.67527
H	0.195416	2.234508	-3.1517
C	-0.79658	4.400383	-0.85474

C	-0.60589	5.914349	-1.04032
H	-0.28442	6.308162	-0.20246
H	-1.46174	6.323646	-1.28637
H	0.049832	6.076382	-1.75022
C	-1.47568	3.824603	-2.11102
H	-0.92499	4.018574	-2.89805
H	-2.35922	4.234114	-2.2216
H	-1.57529	2.854573	-2.01306
C	-1.79601	4.152462	0.293808
H	-1.88581	3.18856	0.446357
H	-2.66899	4.527895	0.053973
H	-1.46839	4.583345	1.110689
C	-1.34745	1.567309	3.129102
H	-0.73725	2.176037	2.72957
P	-3.04032	-1.34601	-0.85626
C	-2.81197	-3.23004	-0.95334
C	-4.05593	-4.05121	-1.36403
H	-4.75475	-3.94699	-0.6848
H	-3.8121	-4.99706	-1.43968
H	-4.3889	-3.72902	-2.22739
C	-2.29131	-3.72745	0.394513
H	-3.00069	-3.65371	1.067193
H	-1.52319	-3.18528	0.668951
H	-2.01652	-4.66544	0.312927
C	-1.68774	-3.54021	-1.97146
H	-2.01284	-3.36896	-2.8802
H	-1.42312	-4.48026	-1.89107
H	-0.91398	-2.96657	-1.78854
C	-3.09309	-0.66333	-2.61476
C	-1.63651	-0.46578	-3.10671
H	-1.21942	-1.34001	-3.25523
H	-1.12659	0.028765	-2.43129
H	-1.64107	0.040328	-3.94663
C	-3.66003	0.780367	-2.57524
H	-3.50535	1.215175	-3.43963
H	-3.21102	1.288585	-1.86831
H	-4.62258	0.747616	-2.39361
C	-3.88263	-1.43386	-3.66015
H	-3.47427	-2.31377	-3.79544
H	-3.87676	-0.93698	-4.50506
H	-4.80701	-1.54564	-3.35392

C	-4.69806	-0.97556	0.010149
C	-4.69738	0.509872	0.434607
H	-3.84529	0.725574	0.866409
H	-5.43261	0.670123	1.062417
H	-4.81542	1.075198	-0.35743
C	-5.96821	-1.25402	-0.81883
H	-6.76114	-1.08463	-0.26882
H	-5.96807	-2.18957	-1.11217
H	-5.98208	-0.66629	-1.60262
C	-4.7688	-1.79816	1.295688
H	-4.87478	-2.74661	1.071544
H	-5.53471	-1.50223	1.831219
H	-3.94376	-1.67413	1.809146

Table S16. Geometry optimized coordinates for **2-CeNpt**.

Atoms	x	y	z
Ce	-0.00918	-0.0065	0.720423
P	0.193859	3.532868	-0.2231
P	-3.14312	-1.47251	-0.47732
P	2.9015	-1.83651	-0.58229
N	-0.10685	2.018682	0.012645
N	-1.69633	-1.11593	0.007218
N	1.840115	-0.96001	0.170036
C	2.048821	3.879574	0.032246
C	2.847672	3.360143	-1.17361
H	2.549404	2.453739	-1.39698
H	3.80187	3.344474	-0.951
H	2.700961	3.950875	-1.94162
C	2.531243	3.014347	1.22604
H	2.034819	3.265102	2.032915
H	3.488685	3.163685	1.371856
H	2.374822	2.067478	1.02741
C	2.436011	5.34261	0.270079
H	2.113807	5.891135	-0.47509
H	3.41123	5.416283	0.332501
H	2.030724	5.655889	1.105676
C	-0.8081	4.592019	1.004662
C	-0.19344	4.456915	2.412697
H	-0.08462	3.507871	2.631892
H	-0.78633	4.877869	3.069738
H	0.680934	4.898808	2.431228

C	-0.90534	6.087375	0.659082
H	-1.35283	6.562855	1.390058
H	-1.42049	6.198903	-0.16686
H	-0.00477	6.45295	0.534695
C	-2.21535	3.997439	1.125897
H	-2.69484	4.117705	0.279836
H	-2.70156	4.452896	1.844663
H	-2.14939	3.041396	1.331068
C	-0.27379	4.029349	-1.99564
C	-1.80144	4.102623	-2.11853
H	-2.19665	3.272677	-1.77865
H	-2.04824	4.220454	-3.05958
H	-2.13525	4.861304	-1.59577
C	0.161051	2.890869	-2.94196
H	1.139481	2.862638	-2.99108
H	-0.2064	3.050977	-3.83616
H	-0.17351	2.035742	-2.59952
C	0.314593	5.356511	-2.49738
H	-0.0392	5.55401	-3.38974
H	1.290896	5.285128	-2.54149
H	0.06717	6.077204	-1.88111
C	-4.41595	-0.2879	0.288717
C	-4.02719	-0.04674	1.756952
H	-4.0986	-0.88732	2.256301
H	-4.63021	0.618369	2.149965
H	-3.10503	0.281785	1.801337
C	-4.27943	1.085954	-0.37482
H	-3.33347	1.339987	-0.40933
H	-4.77757	1.751174	0.144313
H	-4.63983	1.046295	-1.28579
C	-5.87838	-0.7564	0.240625
H	-6.46049	-0.04568	0.5815
H	-5.98293	-1.55822	0.79399
H	-6.12431	-0.96406	-0.68522
C	-3.27591	-1.34396	-2.36962
C	-2.47498	-0.10599	-2.81531
H	-2.907	0.703758	-2.47187
H	-2.44611	-0.07066	-3.79431
H	-1.5621	-0.16351	-2.46354
C	-4.70801	-1.26468	-2.92247
H	-5.21171	-2.05631	-2.63954

H	-4.6787	-1.22895	-3.90145
H	-5.14665	-0.45847	-2.57918
C	-2.57057	-2.55146	-3.02981
H	-1.66686	-2.64291	-2.66196
H	-2.51643	-2.40888	-3.99781
H	-3.08146	-3.3677	-2.84784
C	-3.57775	-3.24659	0.033834
C	-4.78645	-3.86827	-0.68278
H	-5.56517	-3.28252	-0.57784
H	-4.98244	-4.74529	-0.29196
H	-4.5829	-3.97331	-1.63564
C	-2.35023	-4.13739	-0.19279
H	-2.17423	-4.21263	-1.15391
H	-2.51999	-5.0281	0.178958
H	-1.57225	-3.74067	0.251776
C	-3.83668	-3.30072	1.549975
H	-3.08487	-2.88872	2.024632
H	-3.92914	-4.23456	1.83242
H	-4.66103	-2.81336	1.758085
C	2.609459	-1.74624	-2.45896
C	2.258499	-0.28878	-2.81957
H	1.553021	0.034946	-2.22117
H	1.944319	-0.24821	-3.74687
H	3.054933	0.273381	-2.71952
C	1.353732	-2.56436	-2.79774
H	1.552731	-3.51993	-2.70957
H	1.076811	-2.37199	-3.71791
H	0.630606	-2.32323	-2.18182
C	3.762904	-2.22394	-3.35112
H	4.538333	-1.63824	-3.22395
H	3.482946	-2.1959	-4.28986
H	4.004101	-3.1424	-3.10936
C	2.774007	-3.64671	-0.0216
C	1.283811	-4.01982	0.059079
H	0.806647	-3.34627	0.587397
H	1.18935	-4.89684	0.48605
H	0.906165	-4.05392	-0.8445
C	3.496086	-4.68494	-0.89741
H	3.095256	-4.69349	-1.79171
H	3.40817	-5.57258	-0.49158
H	4.445041	-4.45021	-0.96716

C	3.30395	-3.76408	1.419601
H	4.273964	-3.62514	1.42333
H	3.100249	-4.65593	1.770974
H	2.874628	-3.08569	1.981717
C	4.64951	-1.20713	-0.19224
C	5.800555	-2.18518	-0.47635
H	5.713634	-2.96964	0.104724
H	6.656742	-1.74202	-0.30087
H	5.765084	-2.46785	-1.41403
C	4.676157	-0.78904	1.290066
H	3.95721	-0.14536	1.460864
H	5.540345	-0.37633	1.497578
H	4.547657	-1.57968	1.854758
C	4.922875	0.081163	-0.98384
H	4.98193	-0.12885	-1.93923
H	5.767762	0.476223	-0.6833
H	4.192968	0.717777	-0.83389
C	-0.19695	0.210942	3.211928
H	-1.15	0.387584	3.364348
H	0.257016	1.016796	3.519514
C	0.246367	-0.89152	4.173375
C	-0.36472	-2.22052	3.746191
H	-1.34155	-2.15439	3.771909
H	-0.06855	-2.92726	4.357704
H	-0.07495	-2.43509	2.834943
C	1.760477	-0.99104	4.143643
H	2.058486	-1.16033	3.225171
H	2.051248	-1.72616	4.722111
H	2.149387	-0.15003	4.462916
C	-0.18951	-0.61301	5.624703
H	0.220412	0.221435	5.934419
H	0.099032	-1.35202	6.199955
H	-1.16541	-0.53134	5.66268

Hapticity Assignment of 2-Bn

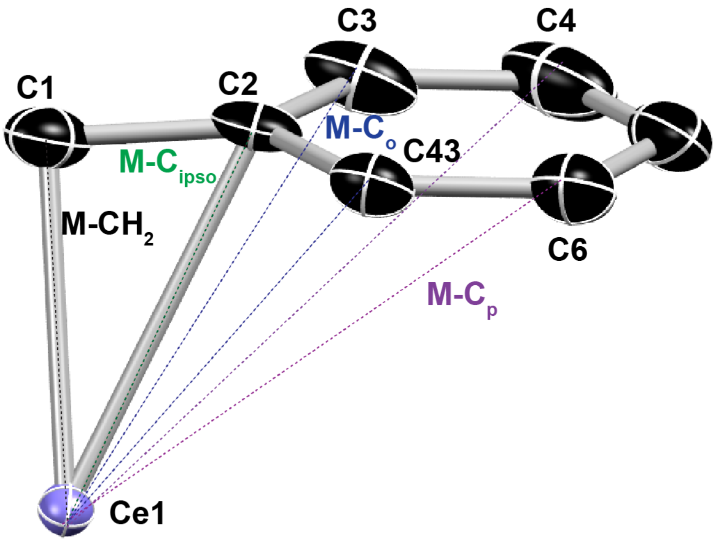

Figure S37. Corresponding M-C distances for hapticity assignment of **2-CeBn**.

Previous studies on *d* and *f* block systems have established that the mode of coordination of benzyl ligands varies from η^1-η^7(Zr)[30], η^2-(Ln(III))[31], η^3-η^4(Th)[32–34]. In the organoactinides Andersen and Zalkin have established another criteria in assigning hapticity. However, this model is only powerful in assigning in assigning hapticity of η^3 and η^4 complexes.[32–34] Therefore, in this study, classifications based on Parkin's work described for $Zr(CH_2Ph)_4$[30] has been used as a criteria, assigning hapticity based on metal-carbon distance as well as M-CH_2-Ph bond angle.

The M-CH_2 distance of 2.562 Å was subtracted from M-C_{ipso}, M-C_o and M-C_p distances, yielding δ_{ipso}, δ_o, and δ_p values used for comparison. These derived values are tabulated in **Table S17**.

M-CH_2-Ph angle of **2-CeBn** is 90.4 °, which satisfies the criteria of ≤97° for η^2 and η^3 complexes. Similarly, δ_{ipso} distance of less than 0.5 Å applies to both η^2 and η^3 complexes. In distinguishing between the two coordination mode of η^2 and η^3 is the δ_o(Å) value at the shorter ortho carbon. In **2-CeBn**, this corresponds to the value derived from Ce1-C43, where δ_o is 0.601 Å. This is larger than 0.5 Å which is the cutoff for classification as η^3 coordination, which designates **2-CeBn** as an η^2 complex.

Table S17. Hapticity assignment of **2-CeBn**

	Angle (°)	M-CH_2 (Å)	M-C_{ipso} (Å)	M-C_o (Å)	M-$C_{o'}$ (Å)	M-C_p (Å)	M-$C_{p'}$ (Å)
**2-CeBn**	90.4	2.562	2.948	3.163	3.866	4.183	4.728
			δ_{ipso} (Å)	δ_o (Å)	$\delta_{o'}$ (Å)	δ_p (Å)	$\delta_{p'}$ (Å)
			0.386	0.601	1.304	1.621	2.166

Comparison of Group 4 (Zr, Hf) and Th Metal-Carbon Distances in Neopentyl Complexes

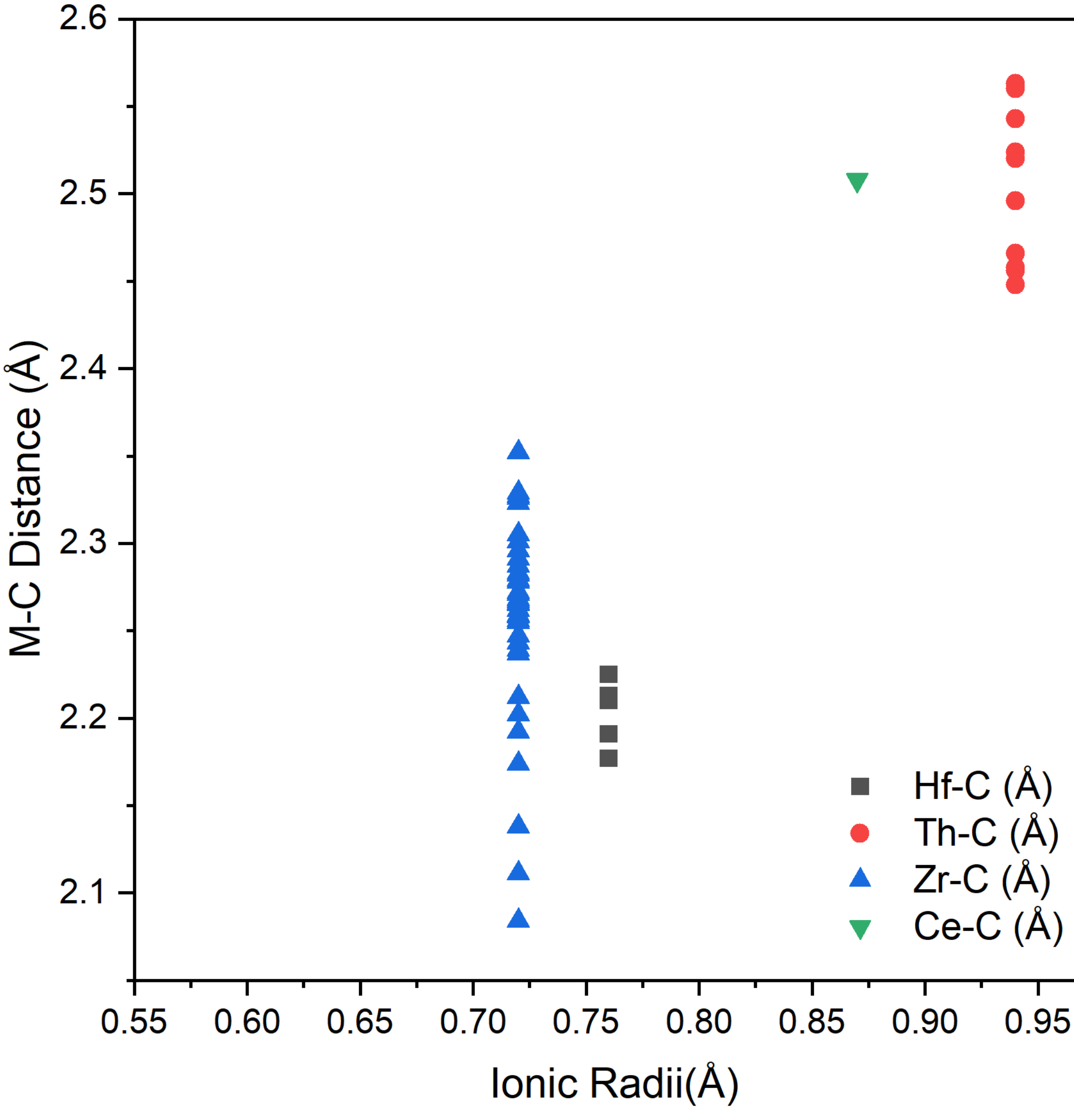

Figure S38. Plot of metal-carbon distances of tetravalent Group 4 (Zr, Hf) and Th complexes with neopentyl ligands versus ionic radii.[35] Metal-carbon distances were tabulated from a CSD search using ConQuest on 12/15/2023 where following number of complexes and independently measured bond distances were found: Zr (23 complexes, 45 bond distances), Hf (2 complexes, 5 distances), Ce (**2-CeNpt**), Th (2 complexes, 11 distances) . For the ionic radii, the six-coordinate radii of the tetravalent oxidation state were used: Zr - 0.72 Å, Hf – 0.71 Å, Ce – 0.87 Å, Th – 0.94 Å.[35]

References

(1) Gompa, T. P.; Rice, N. T.; Russo, D. R.; Aguirre Quintana, L. M.; Yik, B. J.; Bacsa, J.; La Pierre, H. S. Diethyl Ether Adducts of Trivalent Lanthanide Iodides. *Dalton Trans.* **2019**, *48* (23), 8030–8033.

(2) Buchner, W.; Wolfsberger, W. 31P- Und 13C-Kernresonanzuntersuchungen an N-Trimethylsilyl-Triorganophosphiniminen / Phosphorus-31 and Carbon-13 Nuclear Magnetic Resonance Investigations of N-Trimethylsilyl Triorganophosphine Imines. *Z. Für Naturforschung B* **1974**, *29* (5–6), 328–334.

(3) Wolfsberger, W. Tri(Tert-Butyl)Phosphinimin / Tri(Tert-Butyl)Phosphine Imine. *Z. Für Naturforschung B* **1978**, *33* (12), 1452–1456.

(4) Johnson, S. A.; Kiernicki, J. J.; Fanwick, P. E.; Bart, S. C. New Benzylpotassium Reagents and Their Utility for the Synthesis of Homoleptic Uranium(IV) Benzyl Derivatives. *Organometallics* **2015**, *34* (12), 2889–2895.

(5) Schrock, R. R.; Fellmann, J. D. Multiple Metal-Carbon Bonds. 8. Preparation, Characterization, and Mechanism of Formation of the Tantalum and Niobium Neopentylidene Complexes, $M(CH_2CMe)_3(CHCMe_3)$. *J. Am. Chem. Soc.* **1978**, *100* (11), 3359–3370.

(6) Brammer, L.; Connelly, N. G.; Edwin, J.; Geiger, W. E.; Orpen, A. G.; Sheridan, J. B. Structural Consequences of Electron Transfer Reactions. Pt. 17. One-Electron Oxidation of (η^4-Cot)RhCp: Rearrangements of the Radical Cation and of the Resulting Dimerization Product. *Organometallics* **1988**, *7* (6), 1259–1265.

(7) Wedal, J. C.; Barlow, J. M.; Ziller, J. W.; Yang, J. Y.; Evans, W. J. Electrochemical Studies of Tris(Cyclopentadienyl) Thorium and Uranium Complexes in the +2, +3, and +4 Oxidation States. *Chem. Sci.* **2021**.

(8) Boggiano, A. C.; Studvick, C. M.; Steiner, A.; Bacsa, J.; Popov, I. A.; La Pierre, H. S. Structural Distortion by Alkali Metal Cations Modulates the Redox and Electronic Properties of Ce^{3+} Imidophosphorane Complexes. *Chem. Sci.* **2023**, *14* (42), 11708–11717.

(9) Rice, N. T.; Popov, I. A.; Russo, D. R.; Gompa, T. P.; Ramanathan, A.; Bacsa, J.; Batista, E. R.; Yang, P.; La Pierre, H. S. Comparison of Tetravalent Cerium and Terbium Ions in a Conserved, Homoleptic Imidophosphorane Ligand Field. *Chem. Sci.* **2020**, *11* (24), 6149–6159.

(10) Otte, K. S.; Niklas, J. E.; Studvick, C. M.; Boggiano, A. C.; Bacsa, J.; Popov, I. A.; La Pierre, H. S. Divergent Stabilities of Tetravalent Cerium, Uranium, and Neptunium Imidophosphorane Complexes**. *Angew. Chem. Int. Ed.* **2023**, *62* (34), e202306580.

(11) Rice, N. T.; Su, J.; Gompa, T. P.; Russo, D. R.; Telser, J.; Palatinus, L.; Bacsa, J.; Yang, P.; Batista, E. R.; La Pierre, H. S. Homoleptic Imidophosphorane Stabilization of Tetravalent Cerium. *Inorg. Chem.* **2019**, *58* (8), 5289–5304.

(12) Karunananda, M. K.; Mankad, N. P. E-Selective Semi-Hydrogenation of Alkynes by Heterobimetallic Catalysis. *J. Am. Chem. Soc.* **2015**, *137* (46), 14598–14601.

(13) Feller, D. The Role of Databases in Support of Computational Chemistry Calculations. *J. Comput. Chem.* **1996**, *17* (13), 1571–1586.

(14) Schuchardt, K. L.; Didier, B. T.; Elsethagen, T.; Sun, L.; Gurumoorthi, V.; Chase, J.; Li, J.; Windus, T. L. Basis Set Exchange: A Community Database for Computational Sciences. *J. Chem. Inf. Model.* **2007**, *47* (3), 1045–1052.

(15) Pritchard, B. P.; Altarawy, D.; Didier, B.; Gibson, T. D.; Windus, T. L. New Basis Set Exchange: An Open, Up-to-Date Resource for the Molecular Sciences Community. *J. Chem. Inf. Model.* **2019**, *59* (11), 4814–4820.

(16) Lu, Q.; Peterson, K. A. Correlation Consistent Basis Sets for Lanthanides: The Atoms La–Lu. *J. Chem. Phys.* **2016**, *145* (5), 054111.

(17) Krishnan, R.; Binkley, J. S.; Seeger, R.; Pople, J. A. Self-consistent Molecular Orbital Methods. XX. A Basis Set for Correlated Wave Functions. *J. Chem. Phys.* **1980**, *72* (1), 650–654.

(18) McLean, A. D.; Chandler, G. S. Contracted Gaussian Basis Sets for Molecular Calculations. I. Second Row Atoms,*Z*=11–18. *J. Chem. Phys.* **2008**, *72* (10), 5639–5648.

(19) Hehre, W. J.; Stewart, R. F.; Pople, J. A. Self-Consistent Molecular-Orbital Methods. I. Use of Gaussian Expansions of Slater-Type Atomic Orbitals. *J. Chem. Phys.* **2003**, *51* (6), 2657–2664.

(20) van Lenthe, E.; Baerends, E. J.; Snijders, J. G. Relativistic Regular Two-component Hamiltonians. *J. Chem. Phys.* **1993**, *99* (6), 4597–4610.

(21) van Lenthe, E.; Baerends, E. J.; Snijders, J. G. Relativistic Total Energy Using Regular Approximations. *J. Chem. Phys.* **1994**, *101* (11), 9783–9792.
(22) Schreckenbach, G.; Ziegler, T. Calculation of NMR Shielding Tensors Using Gauge-Including Atomic Orbitals and Modern Density Functional Theory. *J. Phys. Chem.* **1995**, *99* (2), 606–611.
(23) Wolff, S. K.; Ziegler, T. Calculation of DFT-GIAO NMR Shifts with the Inclusion of Spin-Orbit Coupling. *J. Chem. Phys.* **1998**, *109* (3), 895–905.
(24) Wolff, S. K.; Ziegler, T.; van Lenthe, E.; Baerends, E. J. Density Functional Calculations of Nuclear Magnetic Shieldings Using the Zeroth-Order Regular Approximation (ZORA) for Relativistic Effects: ZORA Nuclear Magnetic Resonance. *J. Chem. Phys.* **1999**, *110* (16), 7689–7698.
(25) Adamo, C.; Barone, V. Toward Reliable Density Functional Methods without Adjustable Parameters: The PBE0 Model. *J. Chem. Phys.* **1999**, *110* (13), 6158–6170.
(26) te Velde, G.; Bickelhaupt, F. M.; Baerends, E. J.; Fonseca Guerra, C.; van Gisbergen, S. J. A.; Snijders, J. G.; Ziegler, T. Chemistry with ADF. *J. Comput. Chem.* **2001**, *22* (9), 931–967.
(27) Krykunov, M.; Ziegler, T.; van Lenthe, E. Hybrid density functional calculations of nuclear magnetic shieldings using Slater-type orbitals and the zeroth-order regular approximation. *Int. J. Quantum Chem.* **2009**, *109* (8), 1676–1683.
(28) Van Lenthe, E.; Baerends, E. J. Optimized Slater-Type Basis Sets for the Elements 1–118. *J. Comput. Chem.* **2003**, *24* (9), 1142–1156.
(29) Lu, T.; Chen, F. Multiwfn: A Multifunctional Wavefunction Analyzer. *J. Comput. Chem.* **2012**, *33* (5), 580–592.
(30) Rong, Y.; Al-Harbi, A.; Parkin, G. Highly Variable Zr–CH_2–Ph Bond Angles in Tetrabenzylzirconium: Analysis of Benzyl Ligand Coordination Modes. *Organometallics* **2012**, *31* (23), 8208–8217.
(31) J. Wooles, A.; P. Mills, D.; Lewis, W.; J. Blake, A.; T. Liddle, S. Lanthanide Tri- Benzyl Complexes: Structural Variations and Useful Precursors to Phosphorus-Stabilised Lanthanide Carbenes. *Dalton Trans.* **2010**, *39* (2), 500–510.
(32) Rupasinghe, D. M. R. Y. P.; Baxter, M. R.; Zeller, M.; Bart, S. C. Isolation and Characterization of Elusive Tetrabenzylthorium Complexes. *Organometallics* **2023**, *42* (15), 2079–2086.
(33) Edwards, P. G.; Andersen, R. A.; Zalkin, A. Preparation of Tetraalkyl Phosphine Complexes of the F-Block Metals. Crystal Structure of $Th(CH_2Ph)_4(Me_2PCH_2CH_2PMe_2)$ and $U(CH_2Ph)_3Me(Me_2PCH_2CH_2PMe_2)$. *Organometallics* **1984**, *3* (2), 293–298.
(34) Zalkin, A.; Brennan, J. G.; Andersen, R. A. Dibenzyl[1,2-Bis(Dimethylphosphino)Ethane]Bis(Cyclopentadienyl)Thorium(IV). *Acta Crystallogr. C* **1987**, *43* (3), 421–423.
(35) Shannon, R. D. Revised Effective Ionic Radii and Systematic Studies of Interatomic Distances in Halides and Chalcogenides. *Acta Crystallogr. Sect. A* **1976**, *32* (5), 751–767.
